# Synergistic action of specialized metabolites from divergent biosynthesis in the human oral microbiome

**DOI:** 10.1073/pnas.2504492122

**Published:** 2025-08-19

**Authors:** McKenna Loop Yao, Nicholas A. Zill, Colin Charles Barber, Yongle Du, Peijun Lin, Rui Zhai, Eunice Yoon, Dunya Al Marzooqi, Wenjun Zhang

**Affiliations:** ^a^Department of Chemical and Biomolecular Engineering, University of California Berkeley, Berkeley, CA 94720; ^b^Department of Plant and Microbial Biology, University of California Berkeley, Berkeley, CA 94720; ^c^College of Computing, Data Science, and Society, University of California Berkeley, Berkeley, CA 94720

**Keywords:** natural products, oral microbiome, dental caries, *Streptococcus mutans*

## Abstract

Despite extensive efforts, our understanding of the virulence factors contributing to oral biofilm formation—a hallmark of dental caries—remains incomplete. We present evidence that the specialized metabolism of the oral microbiome is a critical yet underexplored factor in oral biofilm formation. Through microbiome analysis, we identified a hybrid nonribosomal peptide synthetase (NRPS) and polyketide synthase (PKS) encoding biosynthetic gene cluster that correlates with dental caries and is widely represented in oral pathogens, including *Streptococcus mutans*. This gene cluster produces two major mutanoclumpin metabolites, MC-584 and MC-586, which feature molecular scaffolds differing in a C–C macrocyclic linkage. Both metabolites synergistically promote robust biofilm formation of *S. mutans* through a rare dual-metabolite mode of action. Further, each metabolite binds uniquely to the *S. mutans* cell surface, resulting in distinct multicellular morphologies. The biosynthesis of mutanoclumpins employs a unique chemical logic that produces two major products, rare within PKS-NRPS assembly lines. This study underscores the importance of characterizing genes implicated in human diseases through microbiome analysis and lays the foundation for exploring strategies to inhibit streptococci-induced dental caries.

## Significance

Dental cavities, or caries, are one of the most common health issues worldwide, yet the factors that drive harmful bacteria to slime our teeth are not fully understood. Our research reveals that the oral bacteria responsible for cavities produce specialized compounds that play a key role in forming decay-causing biofilms. Using computational microbiome analysis to correlate dental caries with specialized genes from oral bacterial samples, we identified a unique set of bacterial genes that create two molecules, called mutanoclumpins, which work together to promote biofilm formation. By understanding the synthesis and function of these molecules, we provide insights into the hidden chemistry of the oral microbiome, which could lead to innovative ways to prevent or treat cavities.

The human body is inhabited by rich and diverse microbial communities, which are intimately linked to the health and disease of the human host (1, 2). The oral cavity harbors one of the most diverse microbial populations in the human body (3–5), with many oral species also existing as hundreds of genetically distinct strains containing individual “accessory genomes” within their pangenome (6, 7). These unique genes can affect whether a strain of the same species acts as a commensal or pathogen, which can undermine traditional microbiome sequencing analysis techniques that correlate species composition with healthy and diseased states (6–10). Thus, a gene-level correlation with oral health status may reveal previously unknown factors related to oral diseases, such as dental caries, which affects over half of the world’s population and contributes more than $350 billion (USD) to global healthcare costs annually (11, 12).

Among oral microbes, *Streptococcus mutans* is a well-known example of a bacterial species with a large pangenome, containing hundreds of accessory genes identified from over 400 sequenced strains (13–15). Although generally considered a key etiological agent of dental caries in humans (16), the cariogenic potential of various *S. mutans* strains varies widely, driven in part by their ability to form biofilms, produce acid, and thrive in acidic conditions (17, 18). While it is conceivable that accessory genomes lead to cariogenic differences, the roles of most accessory genes remain poorly understood in *S. mutans* (17, 19). Functional characterization of accessory genomes, particularly genes correlated with dental caries, could facilitate the development of new preventive or therapeutic approaches for tooth decay.

Accessory genomes often contain biosynthetic gene clusters (BGCs) that produce specialized metabolites with important biological functions, such as antibiotics, signaling molecules, stress tolerance molecules, or virulence factors (15, 20, 21). BGCs encoding megasynthases, such as polyketide synthases (PKSs) and nonribosomal peptide synthetases (NRPSs), are particularly significant due to their large sizes and high metabolic burden (22). The previously discovered PK/NRP metabolites from the human microbiome have demonstrated remarkable bioactivities, including the well-known examples of genotoxin colibactin (23–26) and antibiotic lugdunin (21, 27). Interestingly, in various *S. mutans* strains, large PK/NRP BGCs are often mutually exclusive, suggesting evolutionarily distinct origins of these BGCs and diverse biological functions, as supported by the study of a few known metabolites including mutanobactin, mutanocyclin, and mutanofactin (28–34). This prompted our continued interest in functional characterization of large unknown PK/NRP BGCs from *S. mutans*. In this study, we used gene-level microbiome analysis to prioritize unknown PK/NRP BGCs and identified one PK-NRP hybrid BGC that is strongly associated with dental caries. In-depth study of this BGC revealed two major metabolites with different molecular scaffolds generated via divergent biosynthesis. Both metabolites work synergistically to promote biofilm formation, which is required for *S. mutans’* virulence. Further mode of action studies suggest distinct physicochemical mechanisms for each metabolite in biofilm formation, which provide deeper insights into complex mechanisms of biofilm formation that have previously been overlooked.

## Microbiome Analysis Correlates *mcg* BGC with Dental Caries.

**Fig. 1.**
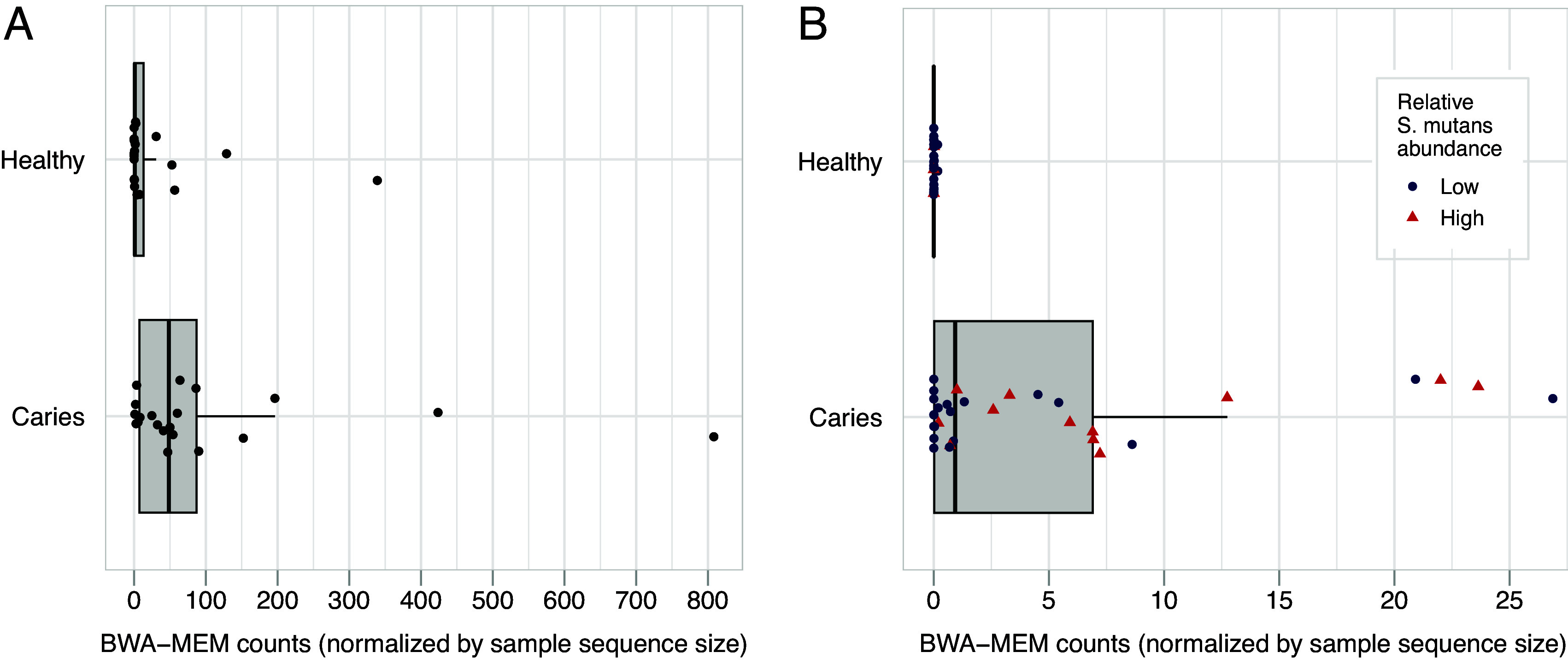
Microbiome analysis of *mcg* in metagenomic and metatranscriptomic datasets. Individual datapoints represent the number of BWA-MEM alignments found for *mcg* in each sample, normalized by the total number of reads in the respective datafile. Only BWA-MEM alignments with scores >50 were included. (A greater genetic presence of *mcg* corresponds to a higher number of BWA-MEM alignment counts). *P*-values were calculated using a two-sided Wilcoxon rank-sum test with a continuity adjustment of 0.5. (The Wilcoxon rank-sum test is a nonparametric method that does not assume normality of the data.) The test statistic (W) represents the sum of ranks for each group. (*A*) Comparison of *mcg* presence in metagenomic samples from supragingival plaque samples of 20 healthy and 20 caries-harboring dentures from children aged 13 to 14 y. (*B*) Comparison of *mcg* expression in metatranscriptomic samples from supragingival plaque samples of 31 healthy and 27 caries-harboring dentures from adults. Relative *S. mutans* abundance was calculated via taxonomic analysis by Mann et al. and classified as high (>5) or low (<5) abundance (41). Samples with high *S. mutans* abundance are marked with red triangles and low are marked with blue circles.

We previously identified three large modular PKS/NRPS encoding hybrid BGCs without an associated product from 57 *S. mutans* clinical isolates from individuals of known dental caries status worldwide (29). To further prioritize these BGCs for functional analysis, we performed metagenomic analysis to determine whether they were associated with disease status. We screened three previously published metagenomic datasets of caries and caries-free plaque samples for sequence similarity matches with the BGCs via BWA-MEM (35) and DESeq2 (36). The three independently sourced datasets consisted of 1) 19 healthy and 25 caries-harboring samples from preschoolers aged 3 to 5 y old (37), 2) 31 healthy and 57 caries-harboring twins aged 10 to 11 y old (38), and 3) 20 healthy and 20 caries-harboring samples from children aged 13 to 14 y old (39). We found that in all three datasets, these BGCs were correlated with dental caries to varying degrees (Fig. 1*A* and *SI Appendix*, Fig. S2), consistent with their patient-origin status. Particularly, one PK-NRP hybrid BGC (BGC5), hereafter referred to as *mcg*, showed a consistently significant log2 fold change between caries and noncaries samples. *Mcg* was further found in 117/435 of the sequenced *S. mutans* genomes published on NCBI, making it the most prevalent unknown PK/NRP BGC in *S. mutans’* pangenome. Genome mining via cblaster (40) revealed that BGCs homologous to *mcg* are present in other Streptococci, Ruminococci, and *Clostridium* genomes (*SI Appendix*, Fig. S3), suggesting a crucial ecological role.

To further explore *mcg’*s role in caries state, we performed additional microbiome analysis on a high-resolution metataxonomic and metatranscriptomic dataset with 31 caries-free and 27active dentin cavity teeth (41). *Mcg* expression was normalized by each sample’s total sequenced reads, and a Wilcoxon rank-sum test indicated that *mcg*’s expression levels differed significantly between the healthy and caries groups [W = 107.5, *P*-value = 7e^−7^ (continuity adjusted by 0.5), Fig. 1*B*]. Notably, minimal *mcg* expression was observed in all caries-free samples regardless of the *S. mutans* abundance, and relatively high *mcg* expression was observed in some caries samples even with a relatively low *S. mutans* abundance (Fig. 1*B* and *SI Appendix*, Fig. S2), suggesting a key role of *mcg* expression in caries development.

## *Mcg* Production of Two Major Metabolites.

**Fig. 2.**
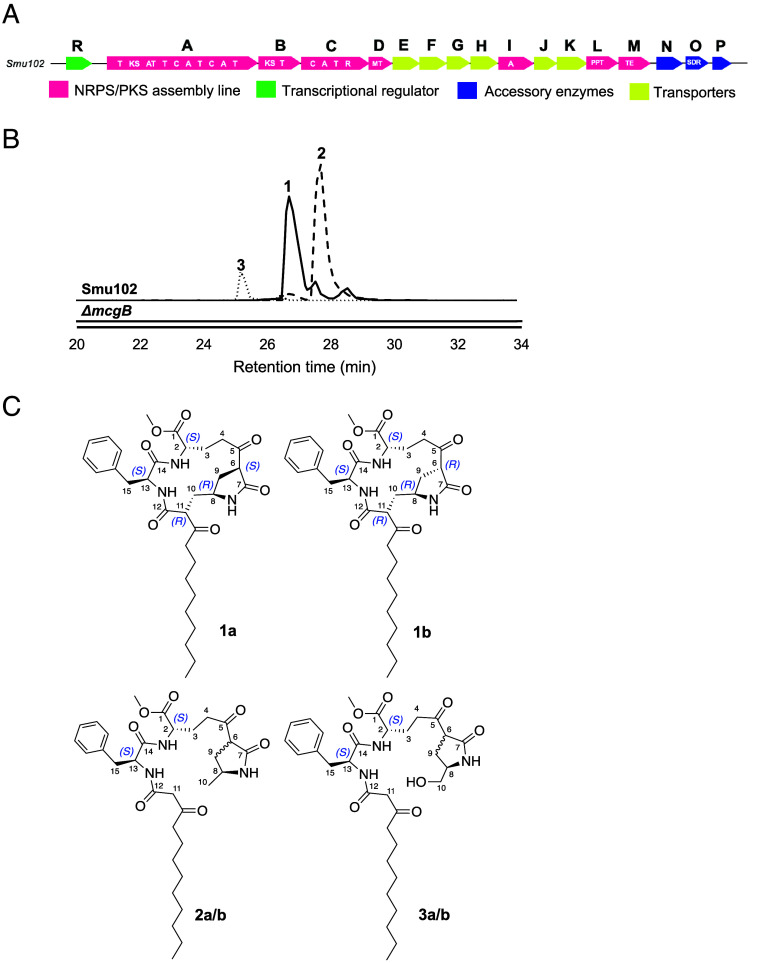
Identified mutanoclumpins from mcg. (*A*) Organization of the mutanoclumpin (mcg) biosynthetic gene cluster in Smu102. Genes encoding NRPS or PKS are shown in pink, accessory enzymes are shown in blue, the transcriptional regulator is shown in green, and transporters are shown in yellow. A, adenylation; C, condensation; KS, ketosynthase; AT, acyltransferase; T, thiolation domain; R, reductase; MT; methyltransferase; PPT, phosphopantetheinyltransferase; TE, thioesterase; SDR, short-chain dehydrogenase/reductase. (*B*) Total ion chromatograms (TICs) from LC-HRMS analysis of culture extracts of *S. mutans* Smu102 and *ΔmcgB*. Deletion of *mcgB* eliminates several peaks visible on the TIC. (*C*) Proposed structures of mutanoclumpins **1a**, **1b**, **2a/b**, and **3a/b**.

To enable further study of the *mcg* BGC, we developed genetic tools for multiple *mcg*-containing *S. mutans* isolates and generated Δ*mcgB*, in which a core PKS-encoding gene was disrupted (Fig. 2*A* and *SI Appendix*, Fig. S4 and Tables S1 and S2). We then performed high-resolution mass spectrometry (HRMS)-based comparative metabolomics using the wild-type (WT) Smu102 and Δ*mcgB*. This revealed two major product masses (*m/z* [M+H]^+^: 584.3347 and *m/z* [M+H]^+^: 586.3504) present in culture extracts of the WT but absent in Δ*mcgB* (Fig. 2*B* and *SI Appendix*, Fig. S4*B*). We additionally found several minor metabolites (including *m/z* [M+H]^+^: 602.3436), whose relationship to the two major metabolites was supported through molecular networking analysis (Fig. 2 and *SI Appendix*, Fig. S4 *B* and *C*). These compounds, collectively termed mutanoclumpins (MCs), were also detected in cultures of other *mcg*^+^ strains, including Smu80, Smu61, and Smu69, but not in their respective *ΔmcgB* strains, strongly supporting the association of MCs with *mcg* (*SI Appendix*, Fig. S5).

The two major mutanoclumpins, MC-584 (**1**) and MC-586 (**2**), and minor metabolite MC-602 (**3**) (hereafter referred to by their *m/z* values), were then targeted for large-scale purification and structural elucidation by NMR spectroscopy. We first purified ~2 mg of **1** as an amorphous white solid from a 10-L fermentation culture of Smu102, and then managed to further separate **1** into two isomers (**1a** and **1b**) at a ~1:1 ratio with the same predicted molecular formula of C_32_H_45_N_3_O_7_. Subsequently, a series of one-dimensional (1D) (^1^H and ^13^C) and two-dimensional (2D) [correlation spectroscopy (COSY), heteronuclear single quantum coherence (HSQC), and heteronuclear multiple-bond coherence (HMBC)] NMR spectra of **1** were acquired, allowing assignment of the carbons and protons for **1** (Fig. 2 and *SI Appendix*, Figs. S6 and S7, Tables S3–S7, and *Note S1*). Briefly, **1** was elucidated as a unique macrocyclic hybrid PK-NRP metabolite that contains a fatty acyl tail, a phenylalanine fragment, a glutamyl methyl ester, and a 2-pyrrolidone moiety. **1a** and **1b** were predicted to be stereoisomers, but the assignment of their absolute configurations was challenging since NOESY and ROESY NMR spectra did not yield useful correlations for multiple stereocenters. We tentatively assigned stereocenters C-2 and C-13 as *S,* considering that these two residues are most likely derived from natural amino acids (l-Glu and l-Phe, respectively) based on the biosynthetic logic discussed below, and the remaining chiral centers as 6*S*, 8*R*, 11*R* in **1a** and 6*R*, 8*R*, 11*R* in **1b** via comparison between the experimental and computational NMR data.

Similarly, ~1.5 mg of **2** and ~1 mg of **3** were purified as amorphous while solids from 10-L and 20-L cultures, respectively. **2** and **3** have predicted molecular formulas of C_32_H_47_N_3_O_7_ and C_32_H_47_N_3_O_8_, respectively, based on HRMS analysis (*SI Appendix*, Fig. S4). These data suggested that **3** might be a hydroxylated product of **2,** which was confirmed by NMR spectra, particularly at C-10 (*SI Appendix*, Figs. S8 and S9, Tables S7–S9, and *Notes S2* and *S3*). The comparison of NMR spectra of **2/3** to **1** indicated that the C–C bond between C-10 and C-11 is cleaved in **2** and **3**. Both **2** and **3** were purified as stereoisomer mixtures, which remained inseparable after multiple trials; thus, we assigned their absolute configurations to be the same as **1a/b**. Additionally, the chemical structures of **1-3** were further confirmed by MS*^2^* data (*SI Appendix*, Fig. S10).

## Divergent Biosynthesis Diversifies Scaffolds of Mutanoclumpins.

**Fig. 3.**
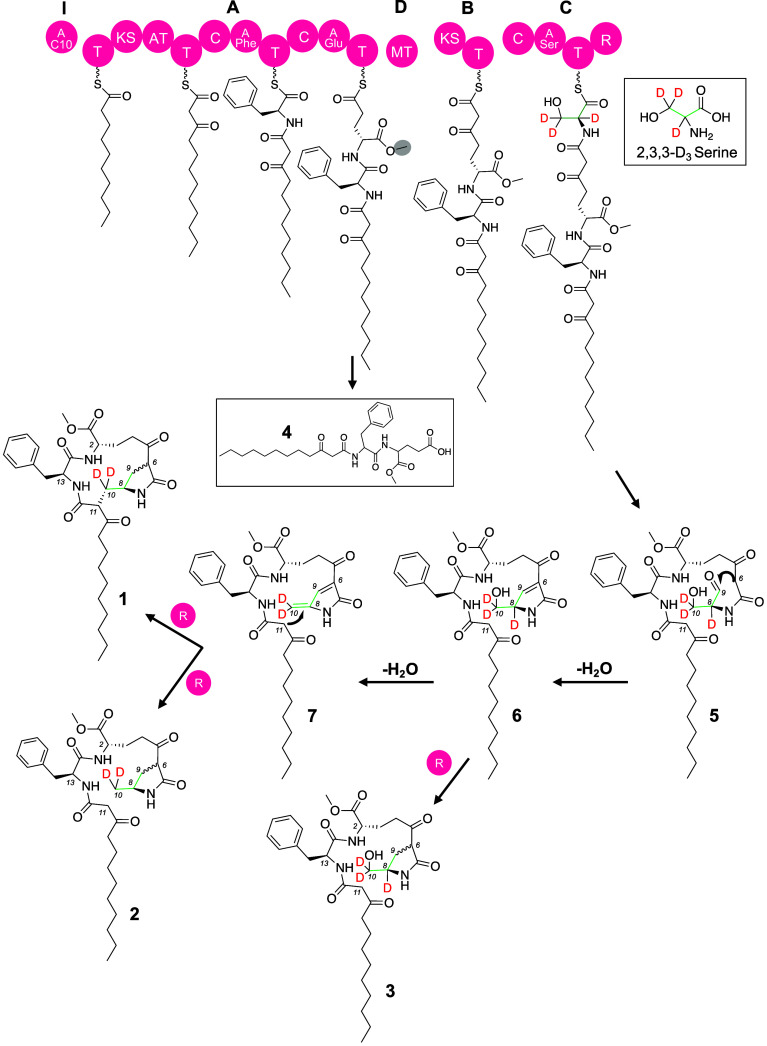
Biosynthesis of mutanoclumpins. Proposed chemical logic for mutanoclumpins’ biosynthesis is mainly revealed using labeled precursor feeding studies.

Bioinformatic analysis of the *mcg* gene cluster allowed us to propose most of the biosynthetic pathway of the mutanoclumpins using the encoded hybrid PKS-NRPS assembly line (Fig. 3). Specifically, a fatty acyl-AMP ligase, McgI, was predicted to activate and load decanoic acid onto McgA to initiate chain assembly. McgA, a three-module PKS-NRPS, extends a ketide unit from malonyl-CoA, followed by condensation with l-Phe and l-Glu via amide linkage. Notably, the γ-carboxylic acid group of Glu, instead of the typical α-carboxylic acid, seemed to be activated and form a thioester bond on McgA. The α-carboxylic acid is likely methylated by the methyltransferase, McgD, as a mass peak corresponding to the expected shunt metabolite **4** was identified from the culture of Smu102 and Δ*mcgB* (*SI Appendix*, Fig. S11). The biosynthesis of mutanoclumpins finishes with McgB-promoted ketide extension and McgC-promoted terminal amino acid incorporation, followed by reductive release and cyclization, although how cyclization differs in MC-584 (**1**) and MC-586 (**2**) remains elusive. To probe if additional biosynthetic enzymes are required for mutanoclumpin biosynthesis, we systematically disrupted putative biosynthetic genes within or in proximity to *mcg.* **1** and **2** were produced in all mutants (*SI Appendix*, Fig. S12), suggesting that the identified set of PKS/NRPS enzymes was sufficient to generate different scaffolds of mutanoclumpins, likely via the activity of the terminal NRPS module encoded by *mcgC*.

It is rare for the same set of PKS-NRPS enzymes to produce two major products with drastically different structures. In this case, an apparent C–C linkage differentiates the “linear” **2** and the “macrocyclic” **1**, raising an intriguing question of how the macrocycle is closed. Considering the observed methyl- vs. hydroxymethyl-modification of the terminal pyrrolidone in **2** and **3**, respectively, we initially hypothesized that the A domain of McgC was flexible in activating Ser for **1** and **3** biosynthesis and Ala for 2 (*SI Appendix*, Fig. S13*A*). However, ^13^C-labeled Ser and Ala feeding experiments showed that only Ser was incorporated into all MCs, including 2 (*SI Appendix*, Fig. S13*B*), indicating that the A domain of McgC was specific for Ser, consistent with bioinformatic prediction (42, 43). Subsequent labeled precursor feeding studies using 1-^13^C-Ser, 3,3-D_2_-Ser, and 2,3,3-D_3_-Ser followed by HRMS and ^13^C NMR analysis enabled us to propose a plausible chemical transformation cascade in synthesizing all observed MCs (Fig. 2). After the terminal Ser incorporation, the terminal reductase (R) domain promotes a two-electron reductive release to yield an aldehyde (44) (**5**), followed by a facile aldol condensation to form a five-membered lactam ring in **6**. This step was supported by the significant enrichment of the C-9 but not C-10 in **1** upon 1-^13^C-Ser incorporation based on NMR analysis (*SI Appendix*, Fig. S14). Dehydration of **6** yields **7**, and the main pathway diverges from **7** in generating two major products, **1** and **2**. Specifically, macrocyclization via Michael addition followed by two-electron reduction yields **1**, while a total of four-electron reduction without Michael addition affords **2**. Consistently, the deuterium at C-2 of Ser was lost (during dehydration of **6**), but the two deuterium atoms at C-3 of Ser were retained in both **1** and **2** based on the 3,3-D_2_-Ser and 2,3,3-D_3_-Ser feeding studies. The production of the minor product **3** was due to the premature two-electron reduction of **6** before dehydration, consistent with the observation that all three deuterium atoms retained in **3** upon feeding of the 2,3,3-D_3_-Ser precursor (*SI Appendix*, Fig. S15).

## MC-584 and MC-586 Synergistically Promote Biofilm Formation.

**Fig. 4.**
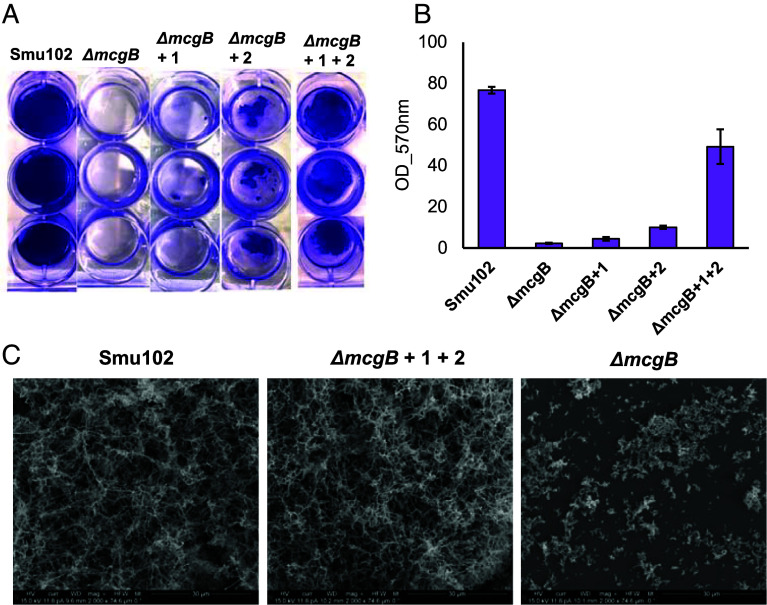
Biofilm formation by Smu102 and *ΔmcgB* with mutanoclumpins. (*A*) Biological triplicates of washed and crystal violet–stained biofilms of Smu102, *ΔmcgB,* and *ΔmcgB* chemically complemented with compounds **1**, **2**, and both. (*B*) Crystal violet-stained biofilms dissolved in ethanol and quantified at OD 570 nm. Error bars represent SD of three biological replicates. (*C*) SEM images of Smu102, chemically complemented *ΔmcgB,* and unwashed *ΔmcgB* biofilms grown on glass coverslips, treated with osmium tetroxide, and imaged at 2,000× magnification with a current of 11.8pA and a voltage of 15.0 kV.

Because the *mcg* BGC was correlated with dental caries, we next probed the direct role of *mcg* in pathogenic traits of *S. mutans* via various phenotypic assays between the WT Smu102 and Δ*mcgB.* No statistically significant growth differences between the two strains were observed when challenged by acids (45), metals (46), and common antibiotics (*SI Appendix*, Fig. S16), suggesting that *mcg* might not affect *S. mutans’* ability to cope with stress. However, we observed WT cellular aggregation in CDM liquid medium with a variety of carbon sources, while *ΔmcgB* dispersed planktonically throughout the culture medium, particularly with glucose, lactose, mannitol, sorbitol, and galactose (*SI Appendix*, Fig. S17). Additionally, in an optimized glucose-containing medium where the WT formed a durable biofilm that resisted washing, *ΔmcgB* exhibited abrogation of biofilm (Fig. 4). While a slight biofilm appeared on the plate surface of the knockout, it was easily sloughed during the washing steps. These results indicated that *mcg* plays a vital role in biofilm formation and maintenance, one of the key pathogenic traits of *S. mutans*.

To directly probe the roles of mutanoclumpins in biofilm formation, chemical complementation was performed using MC-584 (**1**) and MC-586 (**2**), the two major products of *mcg*. Considering significant challenges in separating **1a** and **1b** (which are typically produced in a 1:1 ratio), the purified mixed stereoisomers of **1** were used in the following activity assays. The biofilm phenotype was restored by chemical complementation of *ΔmcgB* with addition of both **1** and **2** at estimated physiological levels based on WT cultures (~8 μM) (Fig. 4). Scanning Electron Microscopy (SEM) further revealed similar cellular arrangement patterns and structures in WT and chemically complemented *ΔmcgB* biofilms (Fig. 4). Neither **1** nor **2** alone could significantly complement biofilm formation, even at elevated twofold concentrations. We further observed a relatively stable production ratio of **1** to **2** from the WT Smu102 in different culture conditions with varying oxidative stress and CO_2_ levels (*SI Appendix*, Fig. S18), suggesting that the biosynthesis of **1** and **2** might be robust to environmental fluctuations or governed by inherent pathway constraints. These results demonstrated a unique ability of *mcg* to produce two structurally unique molecules (“macrocyclic” and “linear”) to act synergistically for successful biofilm formation.

To investigate whether mutanoclumpins exert broader ecological effects within the oral microbiome, we tested mutanoclumpins’ effect on biofilm formation of common neighboring oral species. We supplemented cultures of *Streptococcus sanguinis* SK36*, Streptococcus gordonii* DL-1*, Streptococcus oralis* SK139, and *Lactobacillus casei* ATCC 4646 with estimated physiological levels of both mutanoclumpins (~8 μM) and compared biofilm biomass with and without mutanoclumpin treatment (*SI Appendix*, Fig. S19). Mutanoclumpin addition significantly reduced biofilm formation in *S. sanguinis* and enhanced biofilm formation in *S. oralis. L. casei* exhibited a modest, nonsignificant increase in biofilm biomass, while *S. gordonii* remained unaffected. These results suggest that mutanoclumpins may contribute to niche establishment by promoting *S. mutans* colonization while selectively inhibiting or facilitating the biofilm development of co-occurring species.

## MOA Study of MC-584 and MC-586.

**Fig. 5.**
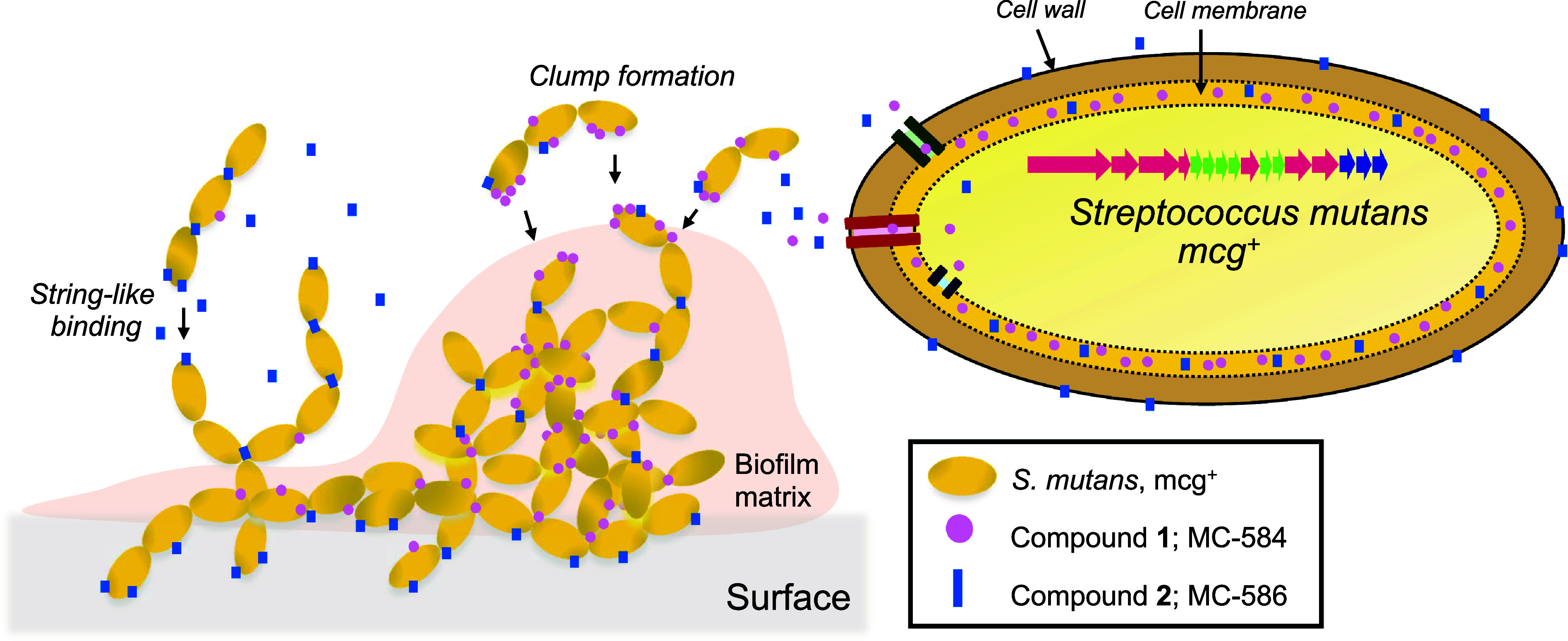
Binding phenotype and localization of mutanoclumpins. (*A*) SEM images of planktonic Smu102, *ΔmcgB,* and chemically complemented planktonic *ΔmcgB* with **1**, **2**, and both, imaged at various magnifications for image clarity with a current of 93.3pA and a voltage of 10.0 kV. (Scale bars are 5 μm.) (*B*) Extraction of **1** and **2** from subcellular fractionation of *ΔmcgB* with **1** and **2** readded independently (*Top*) and natural producer Smu102 (*Bottom*). 2 μg/mL of **1** or **2** was added to *ΔmcgB* in separate experiments. **1** and **2** were extracted simultaneously from Smu102. Error bars represent SD of three biological replicates.

To better understand the mode of action of mutanoclumpins, we next probed the binding behavior of MC-584 (**1**) and MC-586 (**2**) to cells and visualized their subsequent effects on cellular morphology with imaging analysis. The binding data of **2** were like those of previously studied surfactants and small molecules on bacterial surfaces, such as mutanofactin (*SI Appendix*, Fig. S20) (29, 47, 48). In contrast, binding of **1** to *ΔmcgB* was fast and strong, being able to withstand multiple vigorous washing cycles (*SI Appendix*, Fig. S20). Upon binding, **1** mainly localized within the cell membrane, while **2** was found in both the cell wall and membrane, which is also consistent with their natural subcellular locations in the WT (Fig. 5). Notably, both compounds could be recovered using organic solvent extraction, suggesting that they bind to cells noncovalently. We then imaged multiple planktonic cell growth samples using SEM and confocal scanning laser microscopy (Fig. 5 and *SI Appendix*, Fig. S21). We found that most *ΔmcgB* cells were arranged as single cells or short cell-chains that did not aggregate, while WT cells were arranged mostly as cell-chains that formed complex aggregates and three-dimensional clumps around 10 µm in height. The addition of **1** to *ΔmcgB* caused cells to conglomerate in large circular clumps, while **2** caused a “string-like” effect where cells strung together to make longer chains (Fig. 5). When both **1** and **2** were added to *ΔmcgB*, both the clumping and string-like phenotypes were observed. These results showed that both compounds uniquely bound to cells and affected cell physiology.

To probe possible roles of mutanoclumpins as signaling molecules that regulate gene expression of Smu102, we performed RNA-seq analysis of the WT and Δ*mcgB* (*SI Appendix*, Fig. S22). We found that no major *S. mutans* biofilm formation pathways were significantly affected in *ΔmcgB*, including sucrose-dependent biofilm formation factors *gtfB* and *gbpA*, and sucrose-independent biofilm formation factors *spaP*, *atlA*, *rgpG*, and *lytR*. Further, the master regulators *brpA*, *vicR*, and *luxS* were not significantly differentially expressed in *ΔmcgB* (*SI Appendix*, Fig. S23) (16, 18, 49–55). However, we found several lesser-known genes had been significantly differentially expressed in *ΔmcgB*; however, their direct roles associated with biofilm formation remain unknown. Some upregulated pathways in *ΔmcgB* included bacteriocin production and various cellular membrane repair pathways, while some downregulated pathways included genes that might regulate malic acid degradation and virulence factors (*SI Appendix*, Fig. S24). In conclusion, no significant differences in genes known to control the biofilm-forming trait of Smu102 WT and *ΔmcgB* were found across all loci. These results argued against the possibility of mutanoclumpins acting as signaling molecules via regulation of expression of known surface proteins in promoting biofilm formation.

## Discussion

**Fig. 6.**
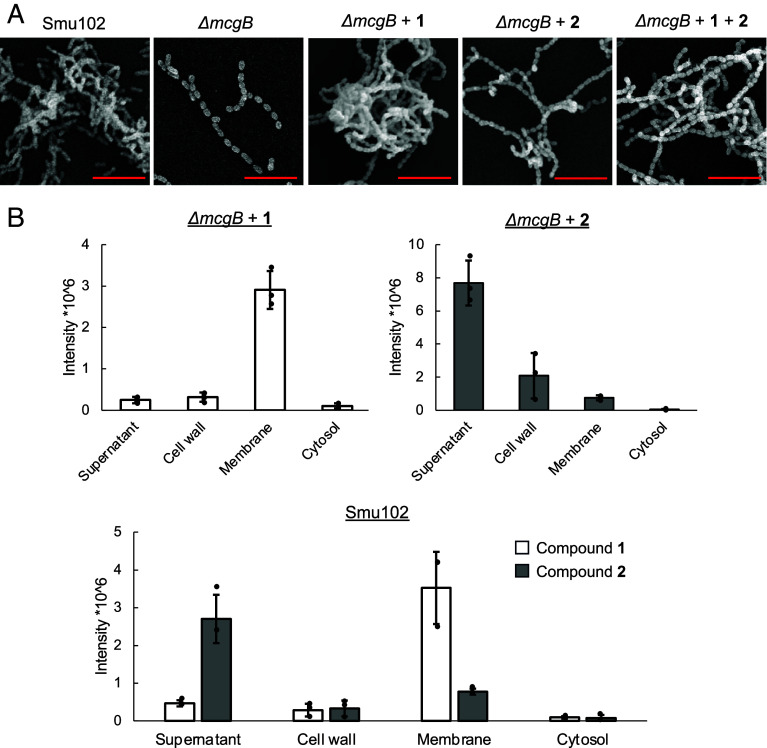
Cartoon depicting prediction of mutanoclumpin mode of action. Pictogram on the left displays *S. mutans* cells forming chains and clumps. Purple circles represent **1** and conglomerate around cells to form clumps. Blue rectangles represent **2** and conglomerate near the edges of cells to form longer chains or bind to the enamel surface. The pictogram on the right displays an enlarged *S. mutans* cell with several membrane transporters, compound **1** mostly within the cell membrane, and compound **2** within the cell membrane and cell wall.

Biofilm formation plays a central role in the development and progression of oral disease, making it a keystone pathogenic trait of *S. mutans* (16, 19). Specifically, the acidification of the *S. mutans’* biofilms inhibits growth of early colonizers like *S. gordonii* and *S. sanguinis* (56–58) while providing a niche for aciduric and acidogenic microbes like *Lactobacillus*, Scardovia, and Bifidobacterium spp. and lactic acid consumers like Veillonella spp (59, 60). Untreated cariogenic biofilms demineralize enamel, leading to the formation of carious lesions and can trigger serious complications like degradation of dentin and periodontal disease (11). Although the macromolecular factors underlying the strong biofilm-forming ability of *S. mutans* are well explained, the role of specialized metabolism is much less understood (15, 19). We recently discovered a linear lipopeptide, mutanofactin, that plays a direct role in *S. mutans* biofilm formation by promoting initial adhesion through a physicochemical mechanism (29). However, the occurrence of mutanofactin in *S. mutans* isolates is low (11/435 of the published *S. mutans* genomes from NCBI), raising the question of whether other specialized metabolites are employed by *S. mutans* to fulfill a similar role. Here, we identified mutanoclumpins as a family of lipopeptides that seems to be much more commonly used by *S. mutans* (117/435 of the published *S. mutans* genomes) to facilitate strong biofilm formation (Fig. 6). Intriguingly, the activity of mutanoclumpins requires two structurally unique lipopeptides, “macrocyclic” MC-584 (**1**) and “linear” MC-586 (**2**). We further revealed that **2** binds in the cell wall and membrane and causes cells to form long-chain strings, whereas **1** mainly binds in the cell membrane and is primarily responsible for the formation of three-dimensional cell clumps. Both compounds are required to act synergistically to form strong biofilms via this rare dual-metabolite mechanism. The BGCs for mutanofactin and mutanoclumpins are mutually exclusive in *S. mutans* strains (29), suggesting evolutionarily distinct origins of these specialized metabolites to fulfill this critical ecological role. Future work will explore direct visualization of mutanoclumpin localization to further elucidate the molecular mode of action of mutanoclumins.

Our preliminary experiments on the effect of mutanoclumpins on neighboring oral species suggest that mutanoclumpins may play a unique role within the broader oral microbiome, potentially influencing interspecies interactions and community biofilm architecture. For example, recent in situ studies in the oral cavity have shown that *S. mutans* can form densely packed, highly acidic cellular microcolonies surrounded by other early oral colonizing pathogens to drive tooth demineralization (61, 62). This observation could be linked to MC-584’s clumping phenotype, which promotes tighter cell aggregation and denser cell clusters. Similarly, chain-like structures, like those promoted by MC-586, are commonly found in biofilm structures and could facilitate biofilm mechanical stability (63). Future studies could investigate how mutanoclumpins modulate multispecies biofilm spatial organization and balance clumping and stringing phenotypes. This could reveal insights into mutanoclumpin’s contributions to cariogenic niche formation and broader ecological function.

The observed synergistic activity of mutanoclumpins raises the question of whether the *mcg* cluster produces the two structurally distinct lipopeptides through a tightly regulated biosynthetic mechanism. We propose that the divergent biosynthesis to generate **1** and **2** could be fine-tuned by the terminal R domain in the PKS-NRPS assembly line. In addition to the two-electron reductive release of an aldehyde, the R domain possibly promotes dehydration of **6**, macrocyclization via Michael addition, and all additional reductions in generating **1-3**. The 8*R* and 11*R* configurations of **1** suggest the R domain has stereo-control in the Michael addition reaction. Notably, the chiral center of C-8 in **3** shall retain from l-Ser based on the proposed biosynthetic pathway, which is consistent with 8*R* in **3**. However, the reduction of the double bond between C-6 and C-9, which appears to be the last step of the reaction promoted by the R domain, has no stereo-control and generates 6*S/R* mixtures in **1-3**. Terminal R domains in NRPSs have been known to catalyze two- and four-electron reductive release to yield an aldehyde or alcohol, which can contribute to important intermediates in a variety of natural products, particularly due to the reactive aldehyde (44). However, the proposed complex reaction cascade in mutanoclumpin biosynthesis is unprecedented, especially the possible control of Michael addition vs. reduction to generate **1** and **2** in a constant ratio. Future investigations are needed to further confirm a biosynthetically regulated mechanism via the R domain in McgC.

In summary, we identified a PK-NRP hybrid BGC, *mcg*, which is widespread in streptococci and other oral pathogens and correlated with dental caries in metagenomics and metatranscriptomics samples. Different from typical natural product BGCs that generate one dominant bioactive metabolite, *mcg* employs a unique chemical logic to produce two major types of a mutanoclumpin-family of lipopeptides, the “macrocyclic” MC-584 and “linear” MC-586. Both compounds are required and act synergistically to directly contribute to strong biofilm formation, a major pathogenic trait of *S. mutans* and the hallmark of caries progression. This work thus highlights the critical role of specialized metabolism in biofilm formation, showcases a rare dual-metabolite mode of action, and reveals an unprecedented complex releasing mechanism in PK-NRP biosynthesis. We further underscore the importance of microbiome analysis to motivate and prioritize the study of specialized metabolism to uncover molecular mechanisms underlying microbial pathogenesis and disease development.

## Methods

### Omics Computational Analysis.

Double-paired metagenomic and metatranscriptomic sequences were first downloaded from NCBI via NIH’s sra-toolkit (Bioprojects PRJNA766357, PRJNA383868, PRJNA380711, and PRJEB60355). Low-quality sequences were then trimmed from the raw Illumina reads with Trimmomatic (64). Then, a BGC sequence database was created and indexed with BWA-MEM (35) for BGC2, BGC5, BGC6, and NZ_LR134491.1 (control BGC for control expression). BGC prevalence was then counted in each sample for all sequence alignments with a BWA-MEM score greater than 50 with default BWA-MEM parameters. These counts were then statistically analyzed via a two-sided Wilcoxon rank-sum test with a continuity adjustment of 0.5. (The Wilcoxon rank-sum test is a nonparametric method that does not assume normality of the data.) The computational pipeline was performed on University of California, Berkeley’s Savio Cluster.

### Annotation of *mcg* Genes.

Genes in *mcg* were characterized in two ways. First, *mcg* was annotated using antiSMASH v6.0 (43). Second, putative gene functions were validated using the NCBI Conserved Domain Database (65). Homologs were identified by querying *mcg* amino acid sequences against the NCBI RefSeq Select database or Protein Data Bank (66).

### Determination of *mcg* Boundaries.

Comparison of *mcg* across *mcg^+^ S. mutans* strains, including LAR01 (RefSeq accession: NZ_CP023477.1), determined the conserved bounds of *mcg*. In Smu102, *mcgN* is found on contig74 and *mcgO* and *mcgP* are found on contig 93; however, in LAR01 and other mcg^+^ strains, they are found on the same contig. PCR was used to resolve contig gaps and confirm colocalization of suspected *mcg* genes in Smu102 (*SI Appendix*, Fig. S25).

### Bacterial Strains and Growth Conditions.

All strains used in this study are listed in *SI Appendix*, Table S1 and were acquired from R. Burne and L. Zeng from University of Florida College of Dentistry. All strains were stored in 20% glycerol at −80 °C. For each experiment, strains were first streaked out on Todd Hewitt Broth (THB) agar plates and grown for 2 d at 37 °C. For *S. mutans,* single colonies were then inoculated into Chemically Defined Medium (CDMB) with 4% (v/v) BD™ Difco™ Brain Heart Infusion at 37 °C in a standing incubator (CDMB). CDM components were prepared as stock solutions as previously described (67, 68). CDM was sterilized by vacuum filtration through a 0.22 μm bottle-top filter (Corning, NY) into a bottle sterilized by autoclaving. BHI and THB were sterilized by autoclaving. Growth of *S. mutans* in liquid was carried out at 37 °C without shaking. Unless specified, cultures were grown with 1 M Dextrose (D-glucose). For experiments, cultures were inoculated with 1% v/v of overnight culture that had been grown for 16 to 18 h.

### Comparative Metabolomic Analysis.

To carry out comparative metabolomic analysis, strains were grown in 5 mL CDM for 16 h. Cultures were extracted with 1 volume of 95:5 ethyl acetate:methanol. Mixtures were vortexed and centrifuged (16,000×g, 5 min). The organic layer was pipetted into a new tube and dried under N_2_, then resuspended in methanol. 100 μL cell culture equivalent was analyzed on an Agilent Technologies 6545 Accurate-Mass QTOF LC-MS instrument after separation on an Agilent Eclipse Plus C18 column (4.6 × 100 mm). Samples were separated using a linear gradient of 2 to 98% acetonitrile (v/v) over 30 min in H_2_O with 0.1% formic acid (v/v) at a flow rate of 0.5 mL/min. MS data were collected at a fragmentation voltage of 110 V. MS/MS data were collected throughout the run method using three fragmentation voltages (10, 30, and 50 V). Data analysis was performed using MassHunter software and MS-DIAL (69).

### Knockout Mutant Construction.

Synthetic oligonucleotides were provided by IDT (Coralville, IA). Oligonucleotides used in this study are presented in *SI Appendix*, Table S2. Phusion polymerase (New England Biolabs, Ipswich, MA) was used for all PCRs. Genetic knockouts were created using a PCR ligation mutagenesis approach to replace knockout regions with either an erythromycin or spectinomycin resistance cassette. pDL278 and pFLAG-NpEm served as templates for the spectinomycin and erythromycin resistance markers. Briefly, linear knockout constructs were synthesized using a three-piece overlap extension PCR (OE-PCR) strategy. 500-bp upstream and downstream homologous regions were amplified from *S. mutans* Smu102 gDNA. DNA uptake was stimulated via XIP according to previously published protocol (70, 71). Synthetic XIP (sXIP; amino acid sequence, GLDWWSL) was synthesized and purified to 96% homogeneity by NeoBioScience. For the selection of antibiotic-resistant colonies after genetic transformation, either erythromycin (50 μg mL^−1^) or spectinomycin (1 mg mL^−1^) was added to the medium and agar plates when needed.

### Large-Scale Purification.

*S. mutans* Smu102 was cultured in 20L of CDMB for 18 h and extracted with an equivalent volume of 95:5 ethyl acetate: methanol. After drying down, all three compounds were purified using a preparative reverse-phase HPLC protocol with an in-house optimized water:ACN method (40 min gradient of 50%:50% to 40%:60%, then hold at 40%:60% for 10 min). The HPLC column was a Luna 5um C18 100A.

### Biofilm Growth Assays of Smu102.

Biofilm formation was measured in a surface-treated sterile tissue culture plate (12-well) from overnight cultures that were subcultured 1:50 into 3 mL of CDMB at 37 °C for 24 h without agitation. After 24 h, culture media were removed via aspiration, and wells were gently washed with 1 mL of PBS. Plates were then stained with 1 mL of 0.1% (w/v) crystal violet solution and incubated at room temperature for 30 min, followed by removal of fluid by aspiration. Wells were washed via immersion into a 4L bucket of DI water four times and then air dried. The plates were photographed, and the wells were destained with 2 mL ethanol for 30 min at room temperature. The destained ethanol was then quantified with OD_570_.

### Treatment of *ΔmcgB* and Other Oral Commensals with **1** and **2**.

*ΔMcgB* was grown from a single colony for 16 h at 37 °C and then reinoculated in fresh CDMB to reach early stationary phase at OD 1.0. Cells were then inoculated at 0.8% v/v into 12-well plates according to the biofilm growth protocol described previously. They were supplemented to a final concentration of 8 μM **1** and 8 μM **2**, or 16 μM of either **1** or **2**. Biofilms were grown for 24 h and quantified via crystal violet assay.

*S. sanguinis* SK36*, S. gordonii* DL-1*, S. oralis* SK139, and *L. casei* ATCC4646 were grown from a single colony for 16 h in BHI medium at 37 °C and then reinoculated at 5% v/v in fresh BHI to reach early exponential phase after 8 h of growth. Cells were then inoculated at 5% v/v into 12-well plates in their respective biofilm formation media [Biofilm medium, BHI + 1% sucrose, Tryptic Soy Broth, and de Man, Rogosa, and Sharpe medium (MRS), respectively]. Biofilm media were prepared according to Li et al. (29) For the mutanoclumpin condition, cultures were supplemented to a final concentration of 8 μM **1** and 8 μM **2**. Biofilms were grown for 24 h and quantified via crystal violet assay.

### SEM.

Biofilm samples were grown as described but with the addition of a pre-ethanol sterilized microglass cover slide submerged in each well. Biofilms grown on slides were washed with 0.85% saline solution and fixated to the slides for 30 min with 2% glutaraldehyde in 0.1 M sodium cacodylate buffer. The samples were washed 3× with 0.1 M sodium cacodylate buffer, treated with 1% osmium tetroxide for 30 min, and washed with buffer again. Next, samples were dehydrated with a series of ethanol dilutions (35%, 50%, 70%, 80%, 95%, and 100%) for 5 min each. Finally, samples were critically point dried with CO_2_ and the Tousimis AutoSamdri 931 Critical Point Dryer and then sputter coated with 5 nm of gold palladium with a Modified CommonWealth Magnetron Sputtering Deposition System. Samples were imaged on a dual-beam FEI Quanta machine. Planktonic samples were prepared from overnight cultures diluted to OD_600_ = 0.6 and fixated for 30 min with 2% glutaraldehyde in 0.1 M sodium cacodylate buffer on poly-L-lysin-treated glass cover slips. Samples were then dehydrated via sequential ethanol dilutions, critically point dried, and sputter coated as described above.

### Subcellular Fractionation.

Subcellular fractionation was performed as previously described with modifications (72). Smu102 and *ΔmcgB* were grown to OD_600_ = 0.5 in CDMB with glucose at 37 °C. For *ΔmcgB* cultures, 2 μg/mL of compounds **1** and **2** were added and incubated at 37 °C for 1 h. After incubation, cultures were centrifuged at 14,000 g for 3 min at 4 °C, and a fraction of the supernatant was removed for compound quantification. Then, cells were washed with sterile PBS and resuspended with protoplasting buffer (40% sucrose in 0.1 M KPO_4_ [pH 6.2] containing the protease inhibitors 0.029 mM pepstatin A, 0.05 mM phenyl-methylsulfonyl fluoride (PMSF), 1.25 mM iodoacetic acid, and 1.25 mM benzamidine). Cell walls were solubilized by the addition of 170 U of mutanolysin (sourced from Thomas Scientific; mutanolysin from *Streptomyces globisporu*) from a stock solution of 3,400 U/mL and digested for 3 h at 37 °C. The protoplasts were centrifuged at 14,000 g for 3 min at 4 °C, and a fraction of the supernatant was removed for compound quantification (cell wall fraction). The cell pellets were then lysed by resuspension in 1 mL of osmotic lysis buffer (50 mM Tris [pH = 7.5], 10 mM MgSO4, 0.8 M NaCl) and lysed for 10 min with water bath sonication and several freeze/thaw cycles. The lysed cells were then ultracentrifuged at 400,000 g at 4 °C for 30 min, and the supernatant was collected as the cytosol fraction. The cell membrane fraction remained in the tube. All fractions were extracted with an equal volume of 95:5 ethyl acetate:methanol and analyzed via single quadrupole LC-MS.

## Supplementary Material

Appendix 01 (PDF)

## Data Availability

The authors declare that all the data supporting the findings of the present study are available within the manuscript, the *SI Appendix*, and/or in the following public data repositories. The MS raw data used for metabolomics have been deposited at the NIH Common Fund’s National Metabolomics Data Repository (NMDR website, the Metabolomics Workbench) (73), where it has been assigned Study ID ST004051 (Project DOI: https://doi.org/10.21228/M86828) (74). The raw RNA-sequencing data have been deposited to the National Center for Biotechnology Information’s Sequence Read Archive database with the BioProject ID PRJNA1183987 (75). The NMR data for the following compounds have been deposited in the Natural Products Magnetic Resonance Database (NP-MRD www.np-mrd.org and can be found at NP0351324 (MC-584a) (76), NP0351325 (MC-584b) (77), NP0351326 (MC-586) (78), and NP0351327 (MC-602) (79). Previously published data were used for this work [NCBI Bioprojects PRJNA766357 (80), PRJNA383868 (81), PRJNA380711 (82), and PRJEB60355 (83)].
