# Supplementary material for: Synergistic action of specialized metabolites from divergent biosynthesis in the human oral microbiome
Source: Proc Natl Acad Sci U S A. 2025 Aug 19;122(34):e2504492122. doi: 10.1073/pnas.2504492122 (PMC12403116; doi:10.1073/pnas.2504492122)
Supplement: Supplementary file 1 — Appendix 01 (PDF) [file pnas.2504492122.sapp.pdf]

Supplementary Information

Synergistic action of specialized metabolites from divergent biosynthesis in the human oral microbiome

McKenna Loop Yao[a,1], Nicholas A. Zill[a,1], Colin Charles Barber[b,1], Yongle Du[a], Peijun Lin[c,2], Rui Zhai[a], Eunice Yoon[a], Dunya Al Marzooqi[a], and Wenjun Zhang[a,3]

[a]Department of Chemical and Biomolecular Engineering, University of California Berkeley, Berkeley, CA 94720
[b]Department of Plant and Microbial Biology, University of California Berkeley, Berkeley, CA 94720
[c]College of Computing, Data Science, and Society, University of California Berkeley, Berkeley, CA 94720

[1]M.L.Y., N.A.Z., and C.C.B. contributed equally to this work.
[2]Peijun Lin conducted this research while she was a visiting student at the University of California, Berkeley.
[3]To whom correspondence may be addressed. Email: wjzhang@berkeley.edu.

Supplementary Tables P2

Supplementary Schemes P12

Supplementary Figures P14

Supplementary Notes P62

Supplementary Methods P64

Supplementary References P67

Supplementary Tables

Table S1: Strains used in this study.

Strain name	Genotype	Characteristics	Source
Smu102 (S1B)	wild-type	= Smu102	Cornejo et al. (2013)[1]
	ΔmcgB	spectinomycin-resistant	this study
	ΔmcgD	spectinomycin-resistant	this study
	ΔmcgM	spectinomycin-resistant	this study
	ΔmcgN	spectinomycin-resistant	this study
	ΔmcgO	spectinomycin-resistant	this study
	Δ1341	erythromycin-resistant	this study
	Δ1346	erythromycin-resistant	this study
	Δ1351	erythromycin-resistant	this study
	Δ1356	erythromycin-resistant	this study
	Δffs	erythromycin-resistant	this study
Smu61 (G123)	wild-type	= Smu61	Cornejo et al. (2013)
	ΔmcgB	spectinomycin-resistant	this study
Smu80 (SF1)	wild-type	= Smu80	Cornejo et al. (2013)
	ΔmcgB	spectinomycin-resistant	this study
Smu69 (NLML4)	wild-type	= Smu69	Cornejo et al. (2013)
	ΔmcgB	spectinomycin-resistant	this study
Smu86 (U2A)	wild-type	= Smu86	Cornejo et al. (2013)
Streptococcus sanguinis SK36	wild-type	= SK36	Xu et al. (2007)[2]
Streptococcus gordonii DL-1	wild-type	= DL-1	UF College of Dentistry
Streptococcus oralis SK139	wild-type	= SK139	UF College of Dentistry
Lactobacillus casei ATCC4646	wild-type	= ATCC4646	UF College of Dentistry

Table S2: Synthetic oligonucleotides used in this study.

Primer	Target	Purpose	Sequence[1]
CB125f	pDL278 *specR*	Amplify spectinomycin resistance casette	ggaggcagattgccttgaat
CB125r	pDL278 *specR*	Amplify spectinomycin resistance casette	ctcaagaattgggagttagtaggc
CB126f	*gyrB*	PCR positive control	agcacaagagtacgatgcca
CB126r	*gyrB*	PCR positive control	cggattcatggttgtttccc
CB137a	*mcgD* UHR	*mcgD* knockout construct with *specR* marker	agagtagggcgaattactgg
CB137b	*mcgD* UHR	*mcgD* knockout construct with *specR* marker	attcaaggcaatctgcctccccacctttcttattacctacaattaattg
CB137c	*mcgD* DHR	*mcgD* knockout construct with *specR* marker	cctactaactcccaattcttgagatgctcgcctttatacagttc
CB137d	*mcgD* DHR	*mcgD* knockout construct with *specR* marker	actagctgcgcatcaatc
CB138a	*mcgM* UHR	*mcgM* knockout construct with *specR* marker	gagaacggtgtctacttgc
CB138b	*mcgM* UHR	*mcgM* knockout construct with *specR* marker	attcaaggcaatctgcctccttcattaatcttcctcctaaaaagac
CB138c	*mcgM* DHR	*mcgM* knockout construct with *specR* marker	cctactaactcccaattcttgaggcctgatggtataagttatgaaaatc
CB138d	*mcgM* DHR	*mcgM* knockout construct with *specR* marker	tgagaccttgatctcctgc
CB139a	*mcgB* UHR	*mcgB* knockout construct with *specR* marker	ctacagatactgagtttgatgagc
CB139b	*mcgB* UHR	*mcgB* knockout construct with *specR* marker	attcaaggcaatctgcctcccattttccattctcatctcccttt c
CB139c	*mcgB* DHR	*mcgB* knockout construct with *specR* marker	cctactaactcccaattcttgaggggatagtctatagtaaaggaggg
CB139d	*mcgB* DHR	*mcgB* knockout construct with *specR* marker	aggagaatcattctccgtatctg
CB140a	*mcgO* UHR	*mcgO* knockout construct with *specR* marker	tggtaaggatgcaactaagg
CB140b	*mcgO* UHR	*mcgO* knockout construct with *specR* marker	attcaaggcaatctgcctccctcctttatttatgatagaattttctgag
CB140c	*mcgO* DHR	*mcgO* knockout construct with *specR* marker	cctactaactcccaattcttgaggatattgttaaccaaatagggtagtc
CB140d	*mcgO* DHR	*mcgO* knockout construct with *specR* marker	caattaactcaagctgatagcc
CB143a	*mcgN* UHR	*mcgN* knockout construct with *specR* marker	cgttcacctttcaccaaac
CB143b	*mcgN* UHR	*mcgN* knockout construct with *specR* marker	attcaaggcaatctgcctcctctgacataaaatgtccccc
CB143c	*mcgN* DHR	*mcgN* knockout construct with *specR* marker	cctactaactcccaattcttgagagaagcggattttatcatttgag
CB143d	*mcgN* DHR	*mcgN* knockout construct with *specR* marker	actcgttgactgttagcaatc
CB227a	Smu102_1356 UHR	Smu102_1356 knockout construct with *eryR* marker	tttaccagcaacgtcttctg
CB227b	Smu102_1356 UHR	Smu102_1356 knockout construct with *eryR* marker	gccatttattatttccttcctcttttaaacatctccttatttttattacac

CB227c	Smu102_1356 DHR	Smu102_1356 knockout construct with *eryR* marker	atattttactggatgaattgttttagtagacataagctggttttt tattatcc
CB227d	Smu102_1356 DHR	Smu102_1356 knockout construct with *eryR* marker	gaccagttgtacgctcc
CB228a	Smu102_1351 UHR	Smu102_1351 knockout construct with *eryR* marker	ctcgcaagtcaaattgattg
CB228b	Smu102_1351 UHR	Smu102_1351 knockout construct with *eryR* marker	gccatttattatttccttcctcttttaaaaaacaccttctttttt agtta
CB228c	Smu102_1351 DHR	Smu102_1351 knockout construct with *eryR* marker	atattttactggatgaattgttttagtagaatggcaggtttga ctcag
CB228d	Smu102_1351 DHR	Smu102_1351 knockout construct with *eryR* marker	actagcagaccaacaaagag
CB229a	Smu102_1346 UHR	Smu102_1346 knockout construct with *eryR* marker	ccgatttacggtcatgacc
CB229b	Smu102_1346 UHR	Smu102_1346 knockout construct with *eryR* marker	gccatttattatttccttcctcttttatcagcagttaccgatttg c
CB229c	Smu102_1346 DHR	Smu102_1346 knockout construct with *eryR* marker	atattttactggatgaattgttttagtagaatgaatacaatca ccaagcca
CB229d	Smu102_1346 DHR	Smu102_1346 knockout construct with *eryR* marker	aggcaaatcatcaaaccaac
CB230a	Smu102_1341 UHR	Smu102_1341 knockout construct with *eryR* marker	gtggaattggctacacctc
CB230b	Smu102_1341 UHR	Smu102_1341 knockout construct with *eryR* marker	gccatttattatttccttcctcttttatcattttgttctcctgtatc taa
CB230c	Smu102_1341 DHR	Smu102_1341 knockout construct with *eryR* marker	atattttactggatgaattgttttagtagagttggcagttttttt actttatg
CB230d	Smu102_1341 DHR	Smu102_1341 knockout construct with *eryR* marker	gagctaagcgttcagaatc
CB231a	ffs sRNA UHR	ffs sRNA knockout construct with *eryR* marker	caggaatggctattatcagc
CB231b	ffs sRNA UHR	ffs sRNA knockout construct with *eryR* marker	gccatttattatttccttcctcttttaatagcataaagtaaaaa aactgcc
CB231c	ffs sRNA DHR	ffs sRNA knockout construct with *eryR* marker	atattttactggatgaattgttttagtagagtgtaacagtaag gtaaatagataac
CB231d	ffs sRNA DHR	ffs sRNA knockout construct with *eryR* marker	gaatagcatagtactttgcc
CB144f	Gap between contig 7 and 74	Confirm continuity of *mcg* on Smu102	ggggcaaacgataaaaacattatatg
CB144r	Gap between contig 7 and 74	Confirm continuity of *mcg* on Smu102	cagcagctacatgggcg
CB145f	Gap between contig 74 and 93	Confirm continuity of *mcg* on Smu102	gatgcttgatctgaaacgtcc
CB145r	Gap between contig 74 and 93	Confirm continuity of *mcg* on Smu102	cccacaacaacattatattggtcg

[1]Underlined sequences are complementary to the marker.

Table S3: NMR Data of Compound 1a in DMSO-*d*₆. For positions, refer to Scheme S1A.

Position	δ_H (J in Hz)	δ_C (C type)	Position	δ_H (J in Hz)	δ_C (C type)
1		171.9, C	16		137.3, C
2	4.24 td 8.1, 4.1	49.8, CH	17/21	7.22 m	129.2, CH
3	2.17 m	22.9, CH_2	18/20	7.24 m	128.1, CH
	1.66 m		19	7.17 t 7.3	126.3, CH
4	2.54 m	34.0, CH_2	22	3.59 s	52.1, CH_3
	2.37 dt 18.6, 4.3		23		204.6, C
5		205.1, C	24	2.21 m	39.9, CH_2
6	3.07 d 10.6	57.5, CH		1.94 m	
7		172.4, C	25	1.29 m	22.9, CH_2
8	3.48 m	51.8, CH	26	1.23 m	28.7, CH_2
9	1.98 dd 13.1, 6.4	31.0, CH_2	27	1.23 m	28.8, CH_2
	1.84 m		28	1.23 m	28.9, CH_2
10	2.24 m	32.1, CH_2	29	1.23 m	28.9, CH_2
	1.28 m		30	1.23 m	31.4, CH_2
11	3.59 m	54.7, CH	31	1.26 m	22.2, CH_2
12		167.5, C	32	0.86 t 6.8	14.0, CH_3
13	4.74 td 9.2, 6.4	53.2, CH	2-NH-	8.97 br	
14		171.4, C	7-NH-	8.27 s	
15	2.97 dd 13.9, 6.4	37.6, CH_2	12-NH-	9.29 br	
	2.81 dd 13.9, 9.2				

Table S4: Predicted ^{1}H NMR Data of Compound 1a in DMSO-*d*$_6$. For positions, refer to Scheme S1A. For stereocenter schematics, refer to Scheme S2.

Position	**1a**	6*R*, 8*R*, 11*R*	6*R*, 8*R*, 11*S*	6*R*, 8*S*, 11*R*	6*R*, 8*S*, 11*S*	6*S*, 8*R*, 11*R*	6*S*, 8*R*, 11*S*	6*S*, 8*S*, 11*R*	6*S*, 8*S*, 11*S*
2	4.24	4.32	4.41	4.36	5.18	4.00	4.04	4.54	4.44
3a	2.17	2.42	2.27	2.46	2.71	2.33	2.32	2.22	2.54
3b	1.66	1.59	1.56	2.01	2.17	1.83	1.72	1.73	2.25
4a	2.54	2.67	2.91	3.12	2.91	3.08	3.57	2.41	3.01
4b	2.37	2.62	2.35	3.07	2.19	2.89	2.84	2.33	2.40
6	3.07	3.01	3.05	3.05	3.52	3.17	3.26	3.60	2.78
8	3.48	3.75	4.19	3.47	4.40	3.94	4.07	3.55	4.06
9a	1.98	2.05	2.39	2.94	2.33	2.11	2.36	2.36	2.01
9b	1.84	2.03	1.97	2.16	2.06	1.50	2.32	1.94	1.97
10a	2.24	1.85	2.09	2.69	2.23	2.57	2.49	2.11	2.27
10b	1.28	1.80	1.88	0.74	2.11	1.82	1.73	2.10	1.88
11	3.59	3.77	3.52	3.26	3.04	3.39	3.25	3.05	3.52
13	4.74	4.62	4.49	4.53	3.86	4.71	3.74	4.53	4.64
15a	2.97	3.18	3.39	3.33	3.56	3.13	3.36	3.37	3.30
15b	2.81	2.89	2.95	2.88	3.30	2.46	3.40	2.96	2.86
17	7.22	7.58	7.53	7.66	7.60	7.57	7.55	7.64	7.47
18	7.24	7.66	7.61	7.71	7.67	7.60	7.65	7.67	7.59
19	7.17	7.61	7.56	7.64	7.58	7.57	7.57	7.60	7.56
20	7.24	7.67	7.64	7.69	7.65	7.61	7.62	7.65	7.62
21	7.22	7.62	7.62	7.64	7.58	7.48	7.50	7.58	7.51
24a	2.21	2.69	2.73	2.53	2.95	2.54	2.77	2.26	2.89
24b	1.94	2.45	2.53	2.21	2.77	2.42	2.75	2.07	2.75
MAE		0.26	0.29	0.35	0.49	0.30	0.42	0.27	0.30
RMSE		0.31	0.36	0.43	0.57	0.34	0.50	0.35	0.39

Table S5: Predicted ^{13}C NMR Data of Compound 1a in DMSO-*d*$_6$. For positions, refer to Scheme S1A. For stereocenter schematics, refer to Scheme S2.

Position	**1a**	6*R*, 8*R*, 11*R*	6*R*, 8*R*, 11*S*	6*R*, 8*S*, 11*R*	6*R*, 8*S*, 11*S*	6*S*, 8*R*, 11*R*	6*S*, 8*R*, 11*S*	6*S*, 8*S*, 11*R*	6*S*, 8*S*, 11*S*
1	171.4	169.4	169.7	168.8	169.1	169.9	171.3	169.2	170.1
2	49.8	52.1	53.2	54.8	53.8	55.3	53.2	53.1	52.3
3	22.9	29.3	32.4	24.6	27.4	24.9	23.2	29.6	24.1
4	34.0	35.6	36.6	36.0	43.0	36.9	38.3	39.1	33.8
5	205.1	207.3	206.0	211.6	217.8	205.8	209.6	207.3	209.4
6	57.5	59.9	59.2	55.1	53.2	56.4	56.6	46.4	57.7
7	172.4	165.4	166.0	165.9	166.1	168.3	164.6	165.8	168.4
8	51.8	51.3	52.6	51.8	50.7	52.7	50.6	52.6	51.6
9	31.0	34.6	31.1	24.6	30.0	27.5	27.6	31.4	35.6
10	32.1	32.6	38.4	35.1	34.6	31.2	34.5	33.0	35.8
11	54.7	56.3	58.2	57.0	56.7	56.2	57.8	59.3	57.3
12	167.5	165.8	164.9	164.4	167.1	163.8	166.8	167.3	163.9
13	53.2	56.1	57.8	56.8	61.2	60.9	60.6	57.9	56.6
14	171.4	166.9	167.7	166.3	166.4	168.4	166.7	167.2	166.4
15	37.6	36.9	34.9	38.1	33.3	36.4	32.8	35.5	36.3
16	137.3	136.0	137.0	136.3	138.5	135.9	138.4	136.8	136.9
17	129.2	125.7	126.1	125.9	125.4	125.3	125.5	125.3	125.8
18	128.1	125.2	125.0	125.2	125.3	125.3	125.2	125.4	125.0
19	126.3	123.2	123.0	123.3	122.8	123.5	122.8	123.2	123.0
20	128.1	125.2	125.2	125.4	125.2	125.4	125.1	125.3	125.0
21	129.2	125.9	124.9	125.5	125.8	125.2	125.8	125.8	125.5
23	204.6	211.9	211.7	205.0	207.7	205.4	207.1	204.3	219.9
24	39.9	42.1	44.2	39.8	40.8	38.6	40.4	38.2	43.5
MAE		2.88	3.43	2.95	3.86	2.61	3.03	3.19	3.22
RMSE		3.42	4.08	3.51	4.79	3.11	3.63	4.06	4.37

Table S6: NMR Data of Compound 1b in DMSO-*d*$_6$. For positions, refer to Scheme S1B.

Position	δ_H (*J* in Hz)	δ_C (C type)	Position	δ_H (*J* in Hz)	δ_C (C type)
1		171.8, C	16		137.1, C
2	4.31 ddd 12.3, 8.4, 3.5	49.4, CH	17/21	7.21 d 7.0	128.9, CH
3	2.17 m	23.2, CH_2	18/20	7.24 m	127.9, CH
	1.61 tt 14.1, 3.5		19	7.18 t 7.0	126.1, CH
4	2.53 m	33.7, CH_2	22	3.60 s	51.7, CH_3
	2.37 dt 18.5, 3.5		23		204.8, C
5		n.d.	24	2.21 m	40.0, CH_2
6	3.07 dd 9.7, 2.2	57.1, CH		1.97 dt 17.6, 7.3	
7		n.d.	25	1.31 m	22.8, CH_2
8	3.49 m	51.5, CH	26	1.23 m	28.6, CH_2
9	1.88 m	30.7, CH_2	27	1.23 m	28.6, CH_2
10	2.24 m	30.1, CH_2	28	1.23 m	28.6, CH_2
	1.29 m		29	1.23 m	28.6, CH_2
11	3.59 m	54.4, CH	30	1.24 m	31.1, CH_2
12		n.d.	31	1.27 m	22.2, CH_2
13	4.78 m	52.5, CH	32	0.85 t 7.0	13.8, CH_3
14		171.2, C	2-NH-	8.79 d 8.4	
15	2.98 dd 13.7, 6.8	37.4, CH_2	7-NH-	8.28 s	
	2.79 dd 13.7, 8.6		12-NH-	9.00 d 9.7	

Table S7: Predicted ^{1}H NMR Data of Compound 1b in DMSO-*d*$_6$. For positions, refer to Scheme S1B. For stereocenter schematics, refer to Scheme S2.

Position	**1b**	6*R*, 8*R*, 11*R*	6*R*, 8*R*, 11*S*	6*R*, 8*S*, 11*R*	6*R*, 8*S*, 11*S*	6*S*, 8*R*, 11*R*	6*S*, 8*R*, 11*S*	6*S*, 8*S*, 11*R*	6*S*, 8*S*, 11*S*
2	4.31	4.32	4.41	4.36	5.18	4.00	4.04	4.54	4.44
3a	2.17	2.42	2.27	2.46	2.71	2.33	2.32	2.22	2.54
3b	1.61	1.59	1.56	2.01	2.17	1.83	1.72	1.73	2.25
4a	2.53	2.67	2.91	3.12	2.91	3.08	3.57	2.41	3.01
4b	2.37	2.62	2.35	3.07	2.19	2.89	2.84	2.33	2.40
6	3.07	3.01	3.05	3.05	3.52	3.17	3.26	3.60	2.78
8	3.49	3.75	4.19	3.47	4.40	3.94	4.07	3.55	4.06
9a	1.88	2.05	2.39	2.94	2.33	2.11	2.36	2.36	2.01
9b	1.88	2.03	1.97	2.16	2.06	1.50	2.32	1.94	1.97
10a	2.24	1.85	2.09	2.69	2.23	2.57	2.49	2.11	2.27
10b	1.29	1.80	1.88	0.74	2.11	1.82	1.73	2.10	1.88
11	3.59	3.77	3.52	3.26	3.04	3.39	3.25	3.05	3.52
13	4.78	4.62	4.49	4.53	3.86	4.71	3.74	4.53	4.64
15a	2.98	3.18	3.39	3.33	3.56	3.13	3.36	3.37	3.30
15b	2.79	2.89	2.95	2.88	3.30	2.46	3.40	2.96	2.86
17	7.21	7.58	7.53	7.66	7.60	7.57	7.55	7.64	7.47
18	7.24	7.66	7.61	7.71	7.67	7.60	7.65	7.67	7.59
19	7.18	7.61	7.56	7.64	7.58	7.57	7.57	7.60	7.56
20	7.24	7.67	7.64	7.69	7.65	7.61	7.62	7.65	7.62
21	7.21	7.62	7.62	7.64	7.58	7.48	7.50	7.58	7.51
24a	2.21	2.69	2.73	2.53	2.95	2.54	2.77	2.26	2.89
24b	1.97	2.45	2.53	2.21	2.77	2.42	2.75	2.07	2.75
MAE		0.25	0.29	0.36	0.50	0.31	0.43	0.27	0.31
RMSE		0.31	0.36	0.44	0.57	0.35	0.51	0.35	0.39

Table S8: NMR Data of Compound 2a/b in DMSO-d_6. For positions, refer to Scheme S1C.

	**2a**		**2b**	
Position	δ_H (*J* in Hz)	δ_C (C type)	δ_H (*J* in Hz)	δ_C (C type)
1		171.8, C		172.1, C
2	4.22 m	51.3, CH	4.26 m	51.2, CH
3	1.89 m; 1.83 m	24.6, CH_2	2.00 m; 1.76 m	24.6, CH_2
4	2.91 m	38.2, CH_2	2.91 m	38.1, CH_2
	2.60 m		2.64 dt 18.9, 6.8	
5		205.6, C		205.7, C
6	3.65 m	54.3, CH	3.64 m	54.9, CH
7		171.6, C		171.9, C
8	2.47 m	30.5, CH_2	2.16 dt 12.8, 8.8	30.4, CH_2
	1.61 ddd 12.8, 9.2, 5.1		1.85 m	
9	3.61 m	47.6, CH	3.56 m	47.1, CH
10	1.08 d 5.7	22.0, CH_3	1.09 d 5.7	22.0, CH_3
11	3.26 d 15.0; 3.22 d 15.0	50.4, CH_2	3.26 d 14.6; 3.21 d 14.6	50.4, CH_2
12		165.9, C		165.9, C
13	4.57 ddd 8.4, 3.5, 3.3	53.7, CH	4.57 ddd 8.4, 3.5, 3.3	53.7, CH
14		171.3, C		171.3, C
15	3.03 dd 14.1, 3.5	37.5, CH_2	3.03 dd 14.1, 3.5	37.5, CH_2
	2.74 dd 14.0, 3.3		2.73 dd 14.0, 3.3	
16		137.7, C		137.7, C
17/21	7.24 m	129.2, CH	7.24 m	129.2, CH
18/20	7.25 m	128.0, CH	7.25 m	128.0, CH
19	7.18 m	126.3, CH	7.18 m	126.3, CH
22	3.61 s	51.9, CH_3	3.61 s	51.9, CH_3
23		204.9, C		204.9, C
24	2.27 m	41.5, CH_2	2.27 m	41.5, CH_2
25	1.33 m	22.4, CH_2	1.33 m	22.4, CH_2
26	1.13 m	28.4, CH_2	1.13 m	28.4, CH_2
27	1.24 m	28.7, CH_2	1.24 m	28.7, CH_2
28	1.24 m	28.9, CH_2	1.24 m	28.9, CH_2
29	1.24 m	28.9, CH_2	1.24 m	28.9, CH_2
30	1.23 m	31.3, CH_2	1.23 m	31.3, CH_2
31	1.27 m	22.1, CH_2	1.27 m	22.1, CH_2
32	0.86 t 7.1	14.0, CH_3	0.86 t 7.1	14.0, CH_3
2-NH-	8.51 d 7.0		8.51 d 7.1	
7-NH-	7.99 s		7.98 s	
12-NH-	8.41 d 8.4		8.40 d 8.4	

Table S9: NMR Data of Compound 3a/b in DMSO-d_6. For positions, refer to Scheme S1D.

	3a		3b	
Position	δ_H (J in Hz)	δ_C (C type)	δ_H (J in Hz)	δ_C (C type)
1		172.1, C		172.1, C
2	4.22 m	51.3, CH	4.26 m	51.2, CH
3	1.90 m; 1.82 m	24.6, CH_2	2.00 m; 1.76 m	25.0, CH_2
4	2.93 m	38.4, CH_2	2.93 m	38.3, CH_2
	2.58 ddd 14.5, 9.2, 5.5		2.64 dt 18.9, 7.5	
5		205.8, C		205.5, C
6	3.62 m	53.9, CH	3.61 m	54.2, CH
7		172.2, C		171.9, C
8	3.49 m	53.5, CH	3.48 m	53.5, CH
9	2.40 m; 1.84 m	25.3, CH_2	2.05 dd 10.3, 5.9	24.6, CH_2
10	3.32 m	64.3, CH_2	3.29 m	64.7, CH_2
11	3.27 d 15.0	50.4, CH_2	3.27 d 15.0	50.4, CH_2
	3.22 d 15.0		3.21 d 15.0	
12		165.9, C		165.9, C
13	4.58 dt 9.0, 4.4	53.6, CH	4.58 dt 9.0, 4.4	53.6, CH
14		171.2, C		171.3, C
15	3.03 dd 13.8, 3.1	37.6, CH_2	3.03 dd 13.7, 4.4	37.6, CH_2
	3.73 dd 13.8, 3.6		2.74 dd 13.7, 3.8	
16		137.7, C		137.7, C
17/21	7.21 m	129.2, CH	7.21 m	129.2, CH
18/20	7.25 m	128.0, CH	7.25 m	128.0, CH
19	7.18 m	126.3, CH	7.18 m	126.3, CH
22	3.61 s	51.9, CH_3	3.61 s	51.9, CH_3
23		204.9, C		204.9, C
24	2.26 t 7.4	41.5, CH_2	2.27 t 7.4	41.5, CH_2
25	1.33 m	22.8, CH_2	1.33 m	22.8, CH_2
26	1.12 m	28.4, CH_2	1.12 m	28.4, CH_2
27	1.23 m	28.7, CH_2	1.23 m	28.7, CH_2
28	1.23 m	28.9, CH_2	1.23 m	28.9, CH_2
29	1.23 m	28.9, CH_2	1.23 m	28.9, CH_2
30	1.23 m	31.3, CH_2	1.23 m	31.3, CH_2
31	1.27 m	22.1, CH_2	1.27 m	22.1, CH_2
32	0.86 t 7.0	14.0, CH_3	0.86 t 7.0	14.0, CH_3
2-NH-	8.48 d 7.5		8.49 d 7.5	
7-NH-	7.95 s		7.95 s	
12-NH-	8.36 d 9.0		8.36 d 9.0	

Supplementary Schemes

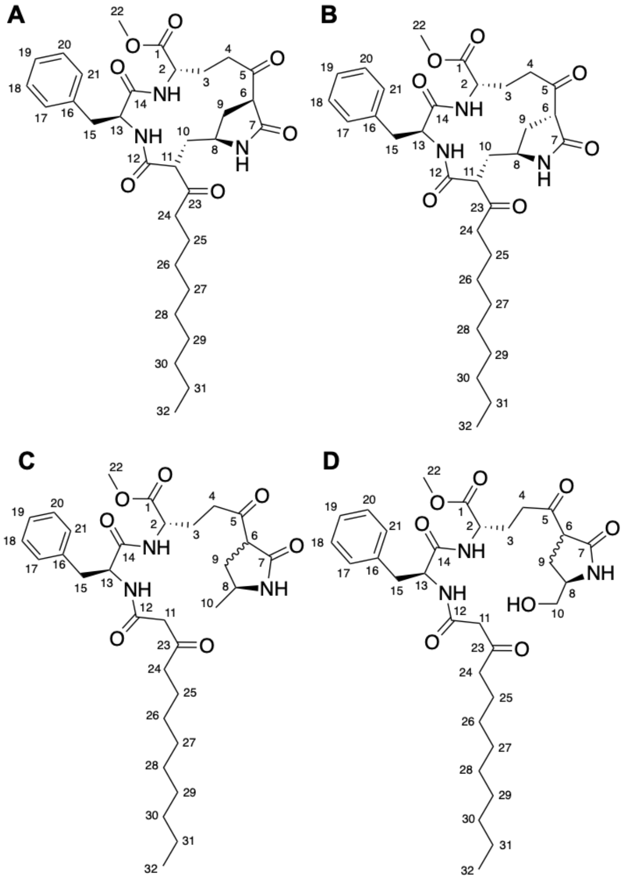

Scheme S1: Compounds 1a (A), 1b (B), 2a/b (C), and 3a/b (D) with carbons numbered. Numbers correspond to Tables S3-S9.

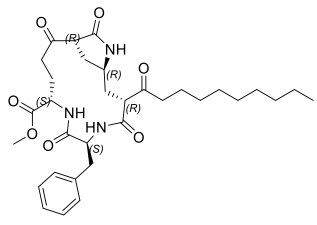

6*R*, 8*R*, 11*R*

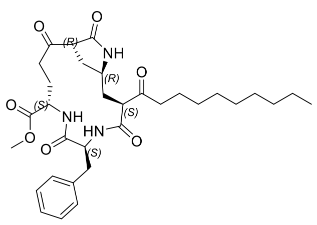

6*R*, 8*R*, 11*S*

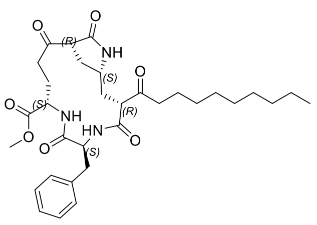

6*R*, 8*S*, 11*R*

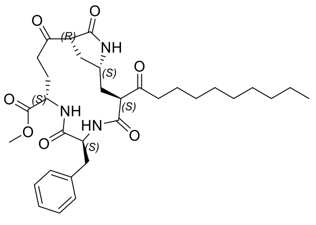

6*R*, 8*S*, 11*S*

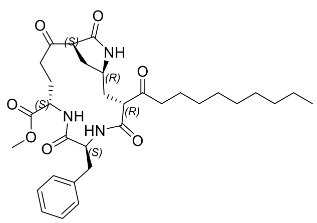

6*S*, 8*R*, 11*R*

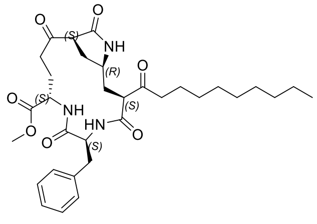

6*S*, 8*R*, 11*S*

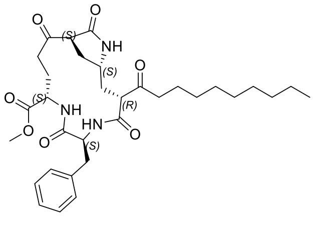

6*S*, 8*S*, 11*R*

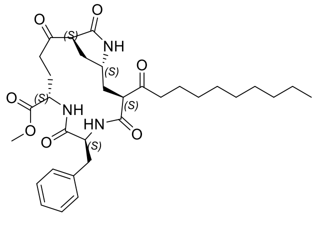

6*S*, 8*S*, 11*S*

Scheme S2: Possible stereochemistry combinations for Compounds 1a/1b. Refer to Tables S4, S5, and S7 for predicted ^{1}H and ^{13}C NMR data for Compounds 1a/1b.

Supplementary Figures

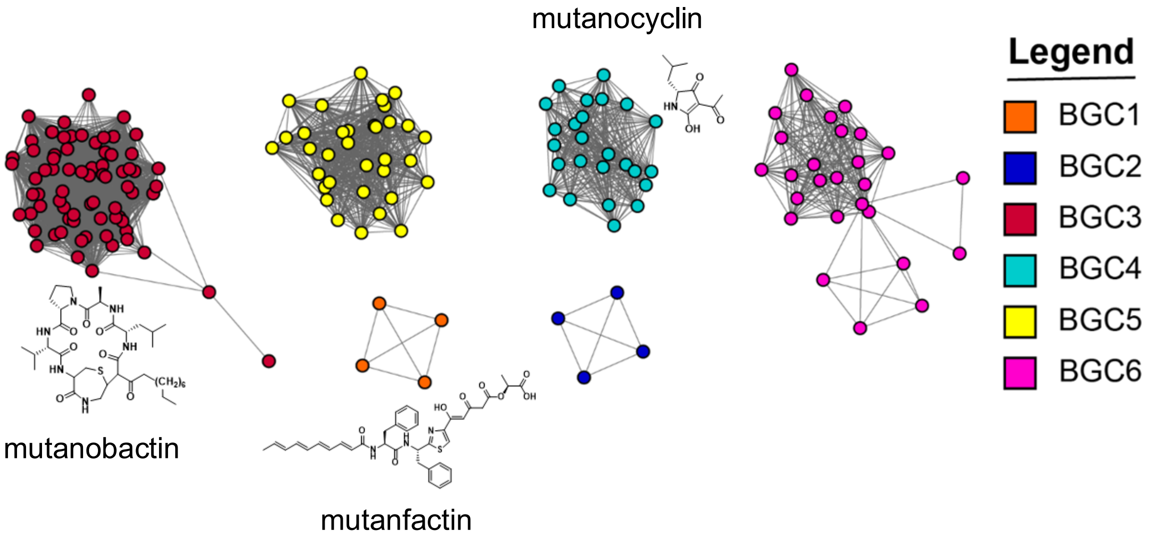

Figure S1: Hybrid NRPS/PKS gene clusters in *S. mutans.* Gene cluster families of six hybrid NRPS/PKS gene clusters were constructed by BiG-SCAPE[3]. Each node represents an NRP/PK producing BGC identified from one *S. mutans* strain's genome. Only genomes published on NCBI before 2021 (165 genomes) are included in this analysis. Each edge is scaled to the squared similarity between each BGC homolog. BGC1, BGC3, and BGC4 produce known products – mutanofactin, mutanobactin, and reutericyclin/mutanocyclin, respectively. BGC2, *mcg* (BGC5), and BGC6 are not associated with a known small molecule product.

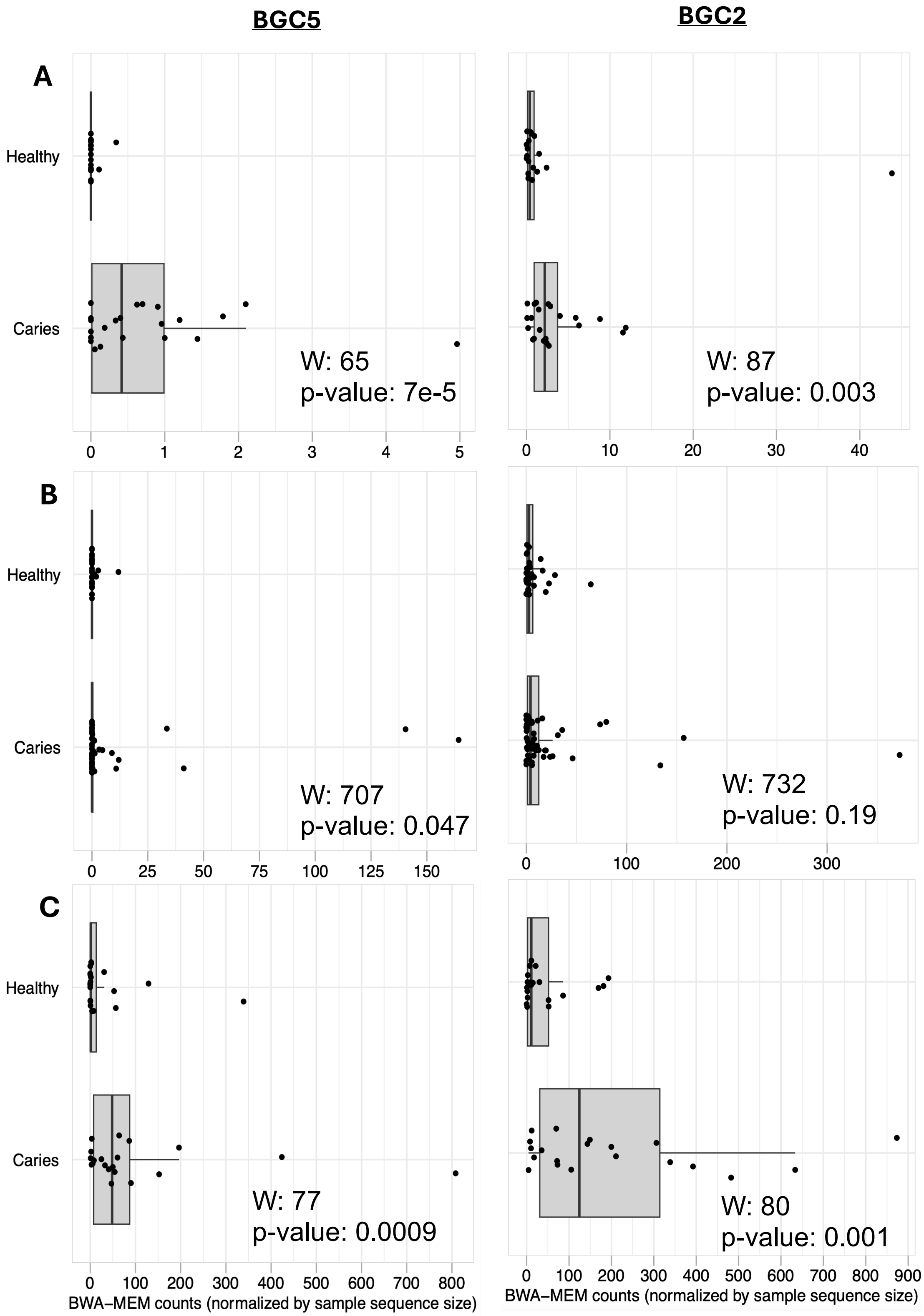
BGC5
BGC2
A
B
C
Healthy
Caries
W: 65
p-value: 7e-5
W: 87
p-value: 0.003
W: 707
p-value: 0.047
W: 732
p-value: 0.19
W: 77
p-value: 0.0009
W: 80
p-value: 0.001
0 1 2 3 4 5
0 10 20 30 40
0 25 50 75 100 125 150
0 100 200 300
0 100 200 300 400 500 600 700 800
0 100 200 300 400 500 600 700 800 900
BWA–MEM counts (normalized by sample sequence size)
BWA–MEM counts (normalized by sample sequence size)

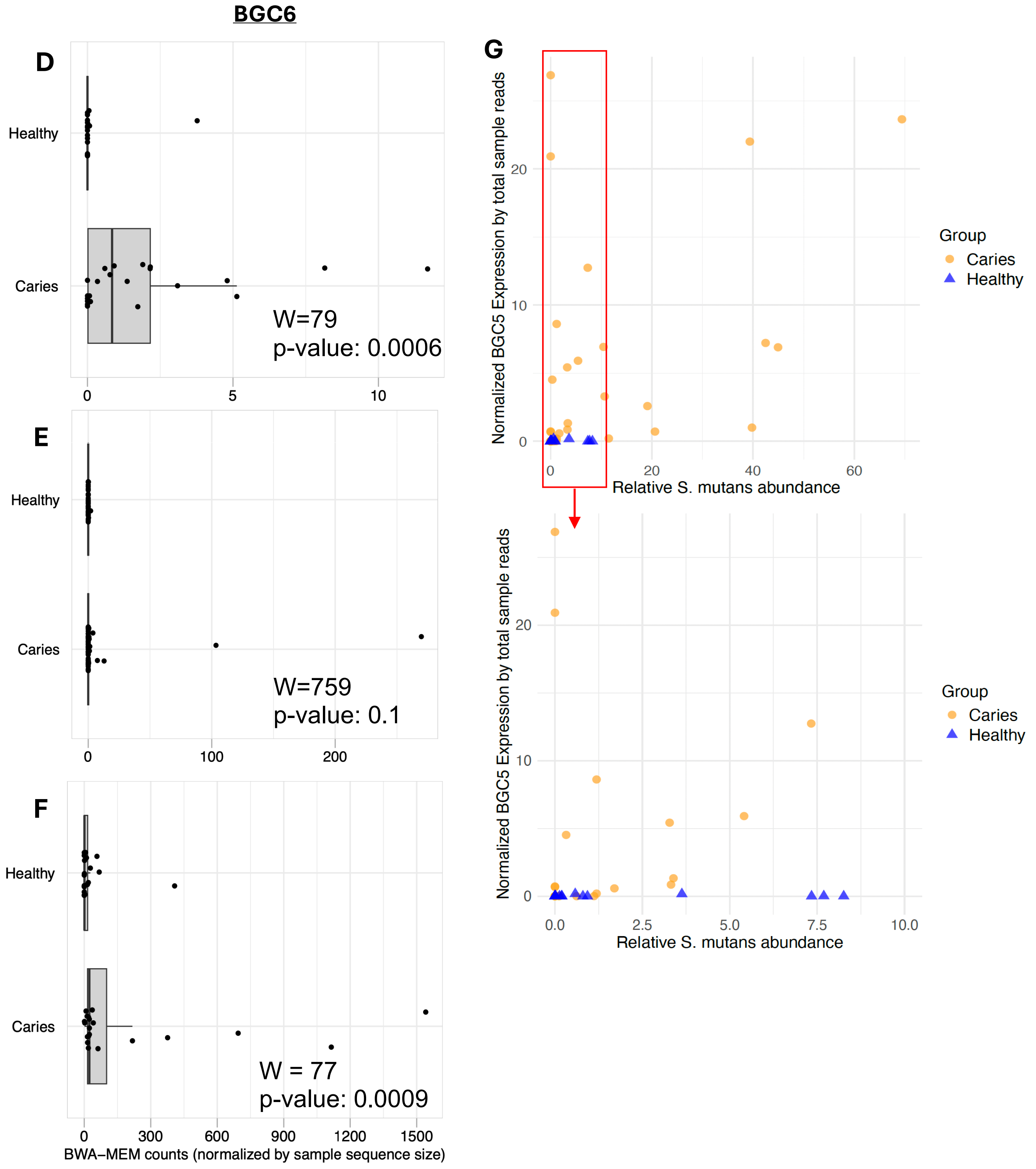

Figure S2: Metagenomic analysis of three hybrid NRP/PK BGCs in three independently sourced datasets. (A-F) Individual datapoints represent the number of BWA-MEM alignments found for the query BGC in each metagenomic sample, normalized by the total number of reads

in the respective metagenomic sample. Only BWA-MEM alignments with scores >50 were included. (A higher prevalence of the BGC corresponds to a higher number of BWA-MEM alignment counts). BGC5 represents *mcg*. Note that x-axis values differ for each dataset to facilitate visual comparison of distributions. The three independently sourced datasets include: (A,D) 19 healthy and 25 caries-harboring samples from preschoolers aged 3–5 years, (B,E) 31 healthy and 57 caries-harboring twins aged 10–11 years, and (C,F) 20 healthy and 20 caries-harboring samples from children aged 13–14 years. p-values were calculated using a two-sided Wilcoxon rank-sum test with a continuity adjustment of 0.5. (The Wilcoxon rank-sum test is a nonparametric method that does not assume normality of the data.) The test statistic (W) represents the sum of ranks for each group. (G) Scatter plot comparing *Streptococcus mutans* abundance (calculated via taxonomic analysis by Mann *et al.*) with expression of *mcg* normalized by the total number of reads in each metatranscriptomic sample. Metatranscriptomic samples were sourced from supragingival plaque of 31 healthy and 27 caries-harboring dentures from adults. Each point represents a metagenomic sample, with *S. mutans* abundance on the x-axis and *mcg* expression on the y-axis. Healthy samples are represented as blue triangles and caries samples as orange circles. The bottom scatter plot is zoomed in for <10 relative *S. mutans* abundance for better distribution visualization. Sample ERR13158684 (PRJEB60355) was excluded from analysis due to mcg counts being over 40-fold higher than the mean BGC count across all samples, indicating it was a statistical outlier.

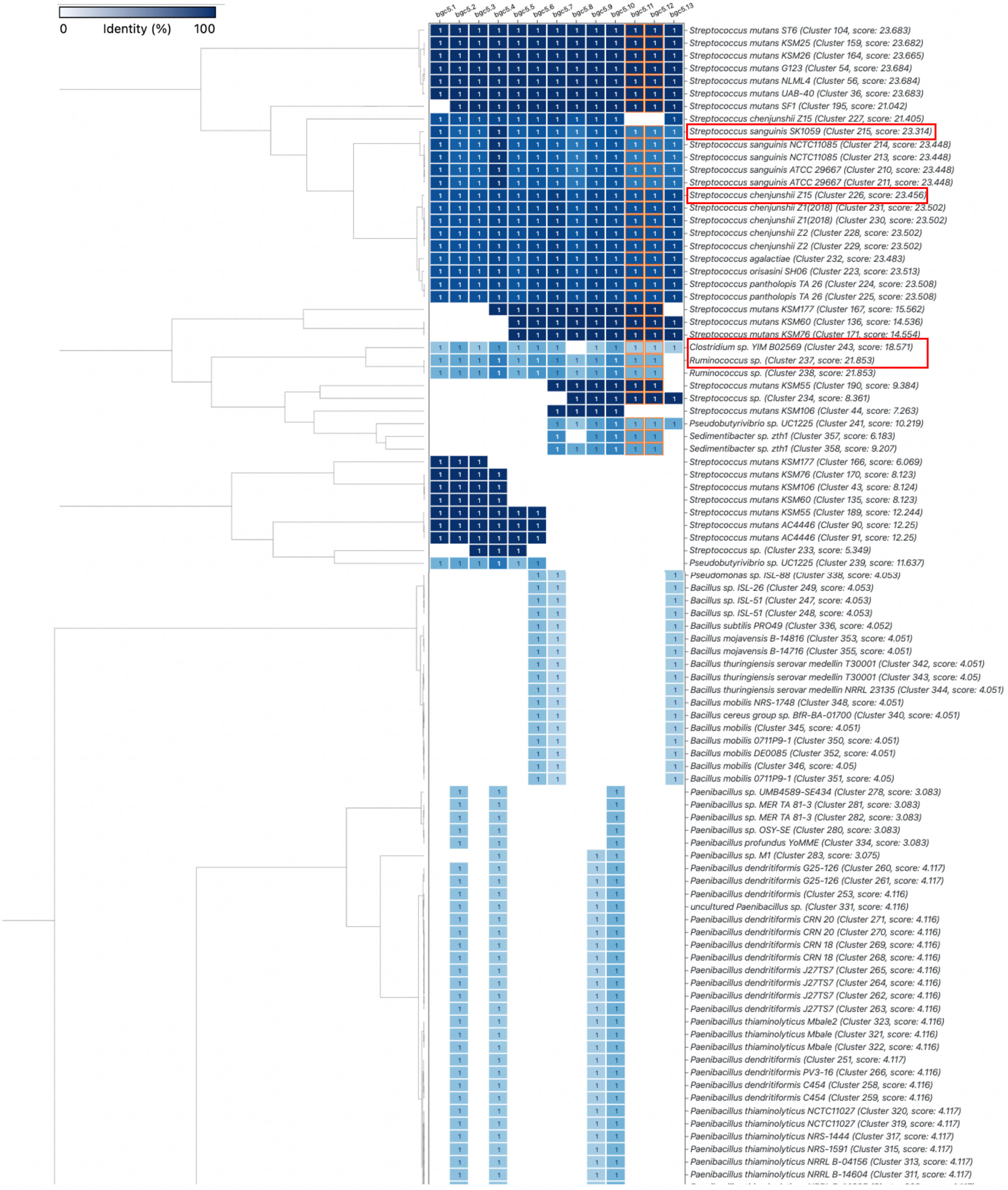

0
Identity (%)
100
bgc5.1
bgc5.2
bgc5.3
bgc5.4
bgc5.5
bgc5.6
bgc5.7
bgc5.8
bgc5.9
bgc5.10
bgc5.11
bgc5.12
bgc5.13
Streptococcus mutans ST6 (Cluster 104, score: 23.683)
Streptococcus mutans KSM25 (Cluster 159, score: 23.682)
Streptococcus mutans KSM26 (Cluster 164, score: 23.665)
Streptococcus mutans G123 (Cluster 54, score: 23.684)
Streptococcus mutans NLML4 (Cluster 56, score: 23.684)
Streptococcus mutans UAB-40 (Cluster 36, score: 23.683)
Streptococcus mutans SF1 (Cluster 195, score: 21.042)
Streptococcus chenjunshii Z15 (Cluster 227, score: 21.405)
Streptococcus sanguinis SK1059 (Cluster 215, score: 23.314)
Streptococcus sanguinis NCTC11085 (Cluster 214, score: 23.448)
Streptococcus sanguinis NCTC11085 (Cluster 213, score: 23.448)
Streptococcus sanguinis ATCC 29667 (Cluster 210, score: 23.448)
Streptococcus sanguinis ATCC 29667 (Cluster 211, score: 23.448)
Streptococcus chenjunshii Z15 (Cluster 226, score: 23.456)
Streptococcus chenjunshii Z1(2018) (Cluster 231, score: 23.502)
Streptococcus chenjunshii Z1(2018) (Cluster 230, score: 23.502)
Streptococcus chenjunshii Z2 (Cluster 228, score: 23.502)
Streptococcus chenjunshii Z2 (Cluster 229, score: 23.502)
Streptococcus agalactiae (Cluster 232, score: 23.483)
Streptococcus orisasini SH06 (Cluster 223, score: 23.513)
Streptococcus pantholopis TA 26 (Cluster 224, score: 23.508)
Streptococcus pantholopis TA 26 (Cluster 225, score: 23.508)
Streptococcus mutans KSM177 (Cluster 167, score: 15.562)
Streptococcus mutans KSM60 (Cluster 136, score: 14.536)
Streptococcus mutans KSM76 (Cluster 171, score: 14.554)
Clostridium sp. YIM B02569 (Cluster 243, score: 18.571)
Ruminococcus sp. (Cluster 237, score: 21.853)
Ruminococcus sp. (Cluster 238, score: 21.853)
Streptococcus mutans KSM55 (Cluster 190, score: 9.384)
Streptococcus sp. (Cluster 234, score: 8.361)
Streptococcus mutans KSM106 (Cluster 44, score: 7.263)
Pseudobutyrivibrio sp. UC1225 (Cluster 241, score: 10.219)
Sedimentibacter sp. zth1 (Cluster 357, score: 6.183)
Sedimentibacter sp. zth1 (Cluster 358, score: 9.207)
Streptococcus mutans KSM177 (Cluster 166, score: 6.069)
Streptococcus mutans KSM76 (Cluster 170, score: 8.123)
Streptococcus mutans KSM106 (Cluster 43, score: 8.124)
Streptococcus mutans KSM60 (Cluster 135, score: 8.123)
Streptococcus mutans KSM55 (Cluster 189, score: 12.244)
Streptococcus mutans AC4446 (Cluster 90, score: 12.25)
Streptococcus mutans AC4446 (Cluster 91, score: 12.25)
Streptococcus sp. (Cluster 233, score: 5.349)
Pseudobutyrivibrio sp. UC1225 (Cluster 239, score: 11.637)
Pseudomonas sp. ISL-88 (Cluster 338, score: 4.053)
Bacillus sp. ISL-26 (Cluster 249, score: 4.053)
Bacillus sp. ISL-51 (Cluster 247, score: 4.053)
Bacillus sp. ISL-51 (Cluster 248, score: 4.053)
Bacillus subtilis PRO49 (Cluster 336, score: 4.052)
Bacillus mojavensis B-14816 (Cluster 353, score: 4.051)
Bacillus mojavensis B-14716 (Cluster 355, score: 4.051)
Bacillus thuringiensis serovar medellin T30001 (Cluster 342, score: 4.051)
Bacillus thuringiensis serovar medellin T30001 (Cluster 343, score: 4.05)
Bacillus thuringiensis serovar medellin NRRL 23135 (Cluster 344, score: 4.051)
Bacillus mobilis NRS-1748 (Cluster 348, score: 4.051)
Bacillus cereus group sp. BfR-BA-01700 (Cluster 340, score: 4.051)
Bacillus mobilis (Cluster 345, score: 4.051)
Bacillus mobilis 0711P9-1 (Cluster 350, score: 4.051)
Bacillus mobilis DE0085 (Cluster 352, score: 4.051)
Bacillus mobilis (Cluster 346, score: 4.05)
Bacillus mobilis 0711P9-1 (Cluster 351, score: 4.05)
Paenibacillus sp. UMB4589-SE434 (Cluster 278, score: 3.083)
Paenibacillus sp. MER TA 81-3 (Cluster 281, score: 3.083)
Paenibacillus sp. MER TA 81-3 (Cluster 282, score: 3.083)
Paenibacillus sp. OSY-SE (Cluster 280, score: 3.083)
Paenibacillus profundus YoMME (Cluster 334, score: 3.083)
Paenibacillus sp. M1 (Cluster 283, score: 3.075)
Paenibacillus dendritiformis G25-126 (Cluster 260, score: 4.117)
Paenibacillus dendritiformis G25-126 (Cluster 261, score: 4.117)
Paenibacillus dendritiformis (Cluster 253, score: 4.116)
uncultured Paenibacillus sp. (Cluster 331, score: 4.116)
Paenibacillus dendritiformis CRN 20 (Cluster 271, score: 4.116)
Paenibacillus dendritiformis CRN 20 (Cluster 270, score: 4.116)
Paenibacillus dendritiformis CRN 18 (Cluster 269, score: 4.116)
Paenibacillus dendritiformis CRN 18 (Cluster 268, score: 4.116)
Paenibacillus dendritiformis J27TS7 (Cluster 265, score: 4.116)
Paenibacillus dendritiformis J27TS7 (Cluster 264, score: 4.116)
Paenibacillus dendritiformis J27TS7 (Cluster 262, score: 4.116)
Paenibacillus dendritiformis J27TS7 (Cluster 263, score: 4.116)
Paenibacillus thiaminolyticus Mbale2 (Cluster 323, score: 4.116)
Paenibacillus thiaminolyticus Mbale (Cluster 321, score: 4.116)
Paenibacillus thiaminolyticus Mbale (Cluster 322, score: 4.116)
Paenibacillus dendritiformis (Cluster 251, score: 4.117)
Paenibacillus dendritiformis PV3-16 (Cluster 266, score: 4.116)
Paenibacillus dendritiformis C454 (Cluster 258, score: 4.116)
Paenibacillus dendritiformis C454 (Cluster 259, score: 4.116)
Paenibacillus thiaminolyticus NCTC11027 (Cluster 320, score: 4.117)
Paenibacillus thiaminolyticus NCTC11027 (Cluster 319, score: 4.117)
Paenibacillus thiaminolyticus NRS-1444 (Cluster 317, score: 4.117)
Paenibacillus thiaminolyticus NRS-1591 (Cluster 315, score: 4.117)
Paenibacillus thiaminolyticus NRRL B-04156 (Cluster 313, score: 4.117)
Paenibacillus thiaminolyticus NRRL B-14604 (Cluster 311, score: 4.117)

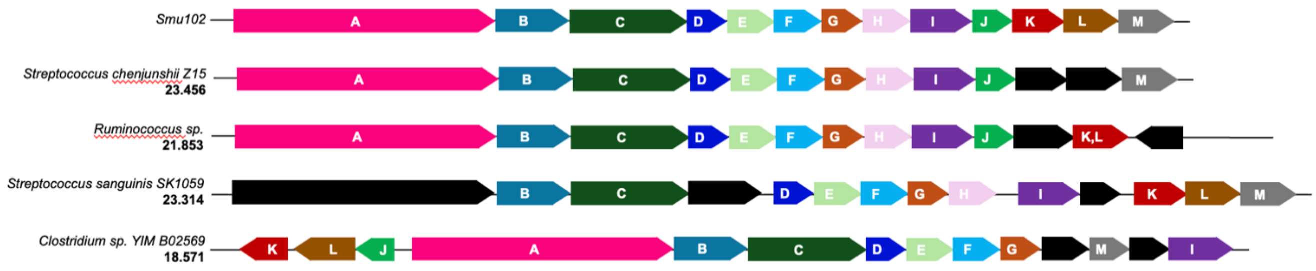

Figure S3: BGCs homologous to *mcg*. The phylogenetic tree is an excerpt of *mcg* homologs found via genome mining with cblaster. Genes BGC5.1-BGC5.13 are genes *mcgA-M* respectively. Strain name and cblaster scores are given for each homolog, and the color scale represents percent gene identity compared to *mcg*. The strains boxed in red were selected to display their extracted *mcg* homologs in the bottom figure, where they are compared to native *mcg* in *Smu102.* Cblaster scores are calculated based on the total amount of hits against the query and a synteny score from query sequence order. Homologs of *mcg* were searched for via cblaster in the NCBI NR database with a 20,000 bp maximum gap size between clusters, a maximum e-value of 0.1, a minimum gene identity of 30%, and a minimum gene coverage of 50%.

(A)

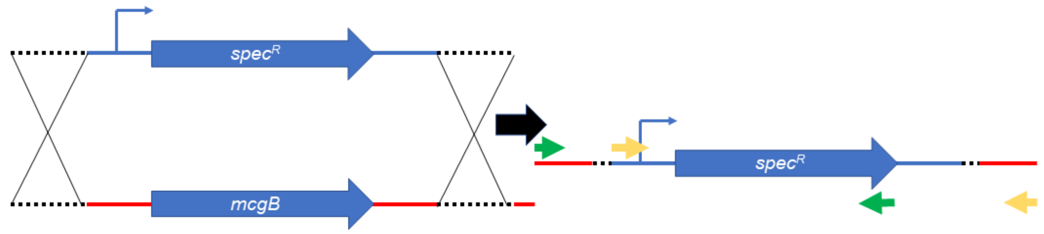

Genotype	Target	Expected size
Δ*mcgB*	*gyrB*	1700
Δ*mcgB*	UHR-spec^R	1800
Δ*mcgB*	spec^R-DHR	1800
wild type	*gyrB*	1700
wild type	UHR-spec^R	--
wild type	spec^R-DHR	--

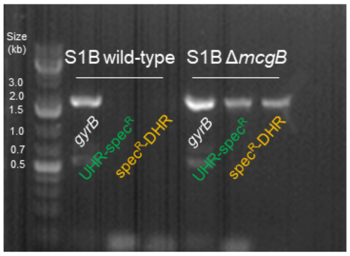

(B)

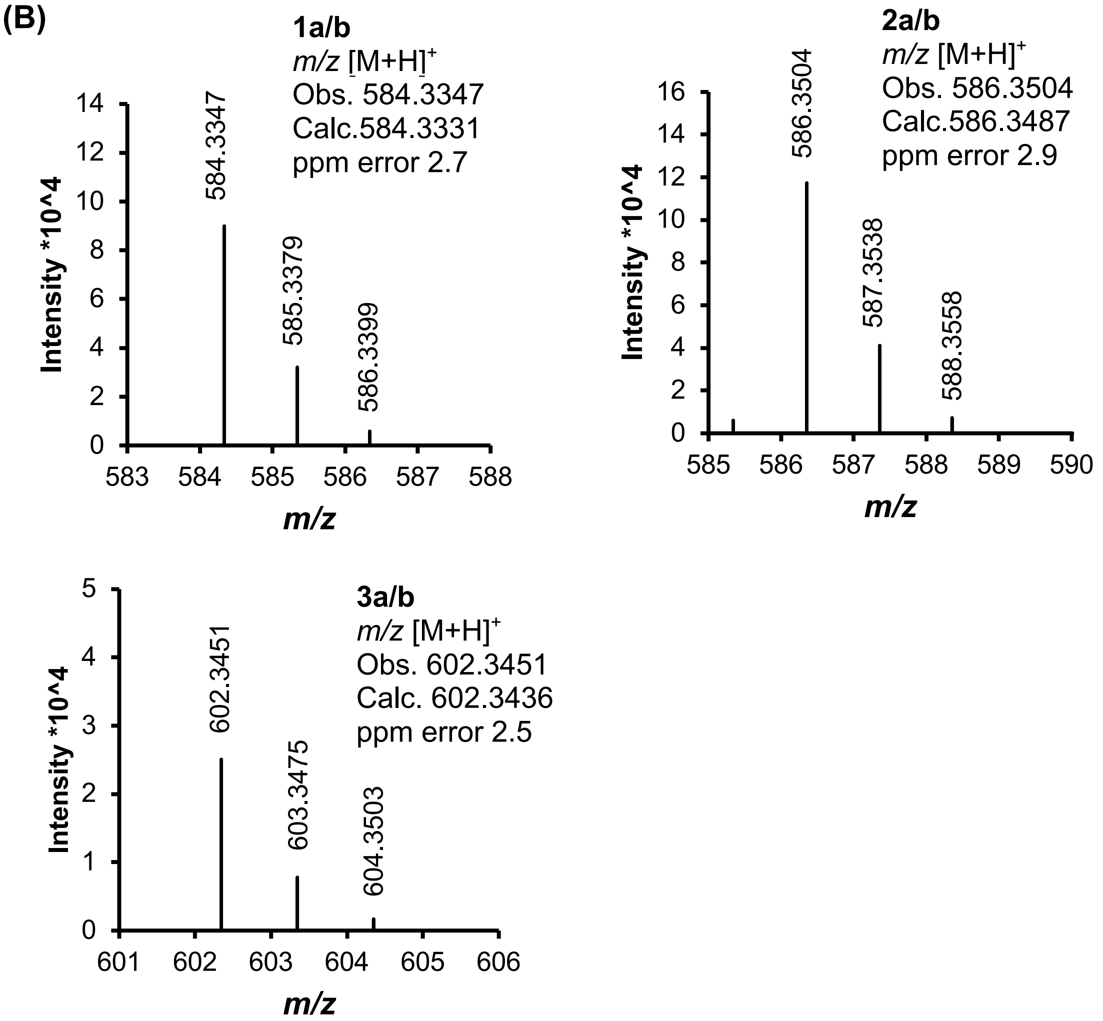

(C)

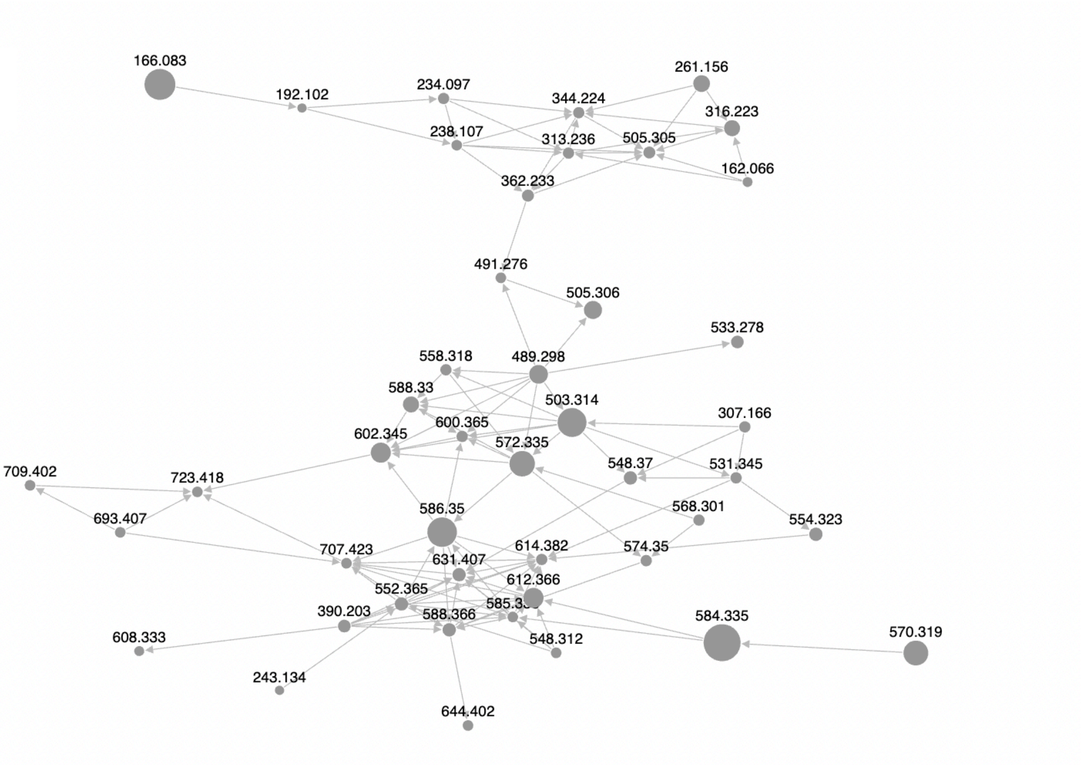

Figure S4: Metabolite identification from *mcg*. (A) Δ*mcgB* generation and verification from representative strain Smu102. As pictured, the DNA was delivered as a linear fragment via double crossover homologous recombination. The table displays the expected band sizes for the colony PCR products of Smu102 and *ΔmcgB*, which are displayed on the agarose gel. *GyrB* was chosen as the positive control housekeeping gene from *S. mutans*. (B) HRMS of compounds **1a/b**, **2a/b**, and **3a/b** with expected and observed *m/z* $[M+H]^+$. (C) GNPS[4] MS/MS molecular network on *mcg* family of metabolites. Grey circles represent parent molecule *m/z*'s and are sized proportional to their relative abundance. Edges were filtered to have a cosine score above 0.4 and more than 4 matched peaks. The data was filtered by removing all MS/MS fragment ions within +/- 17 Da of the precursor *m/z*. MS/MS spectra were window-filtered by choosing only the top 6 fragment ions in the +/- 50Da window throughout the spectrum. The precursor ion mass tolerance was set to .05 Da and a MS/MS fragment ion tolerance of 0.2 Da. The spectra in the network were then searched against GNPS' spectral libraries. All matches kept between network spectra and library spectra were required to have a score above 0.7 and at least 6 matched peaks.

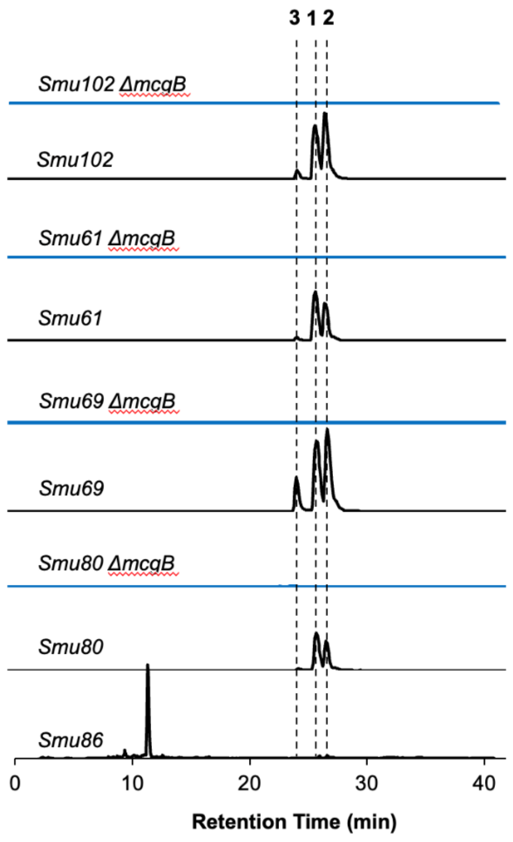

Figure S5: Consistent mutanoclumpin production across *mcg*$^{+}$ strains. *Mcg*$^{+}$ strains *Smu102, Smu61, Smu69,* and *Smu80,* their respective *ΔmcgB* knockouts, and *mcg*$^{-}$ strain *Smu86* were extracted for mutanoclumpin production. Extracted EICs from HR-LCMS for compounds **1**, **2**, and **3** are shown.

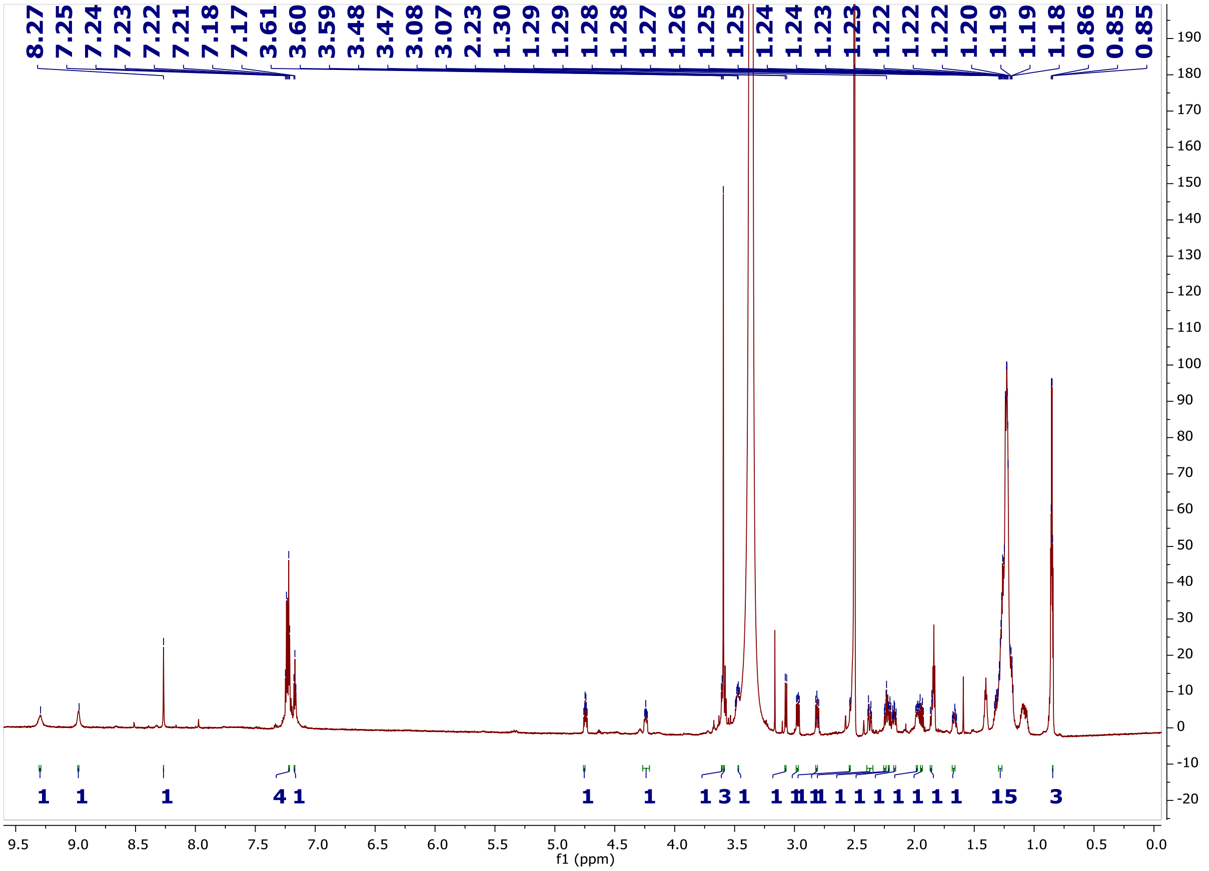

Figure S6a: ^{1}H NMR spectrum of Compound **1a**, DMSO-d_6 at 900MHz.

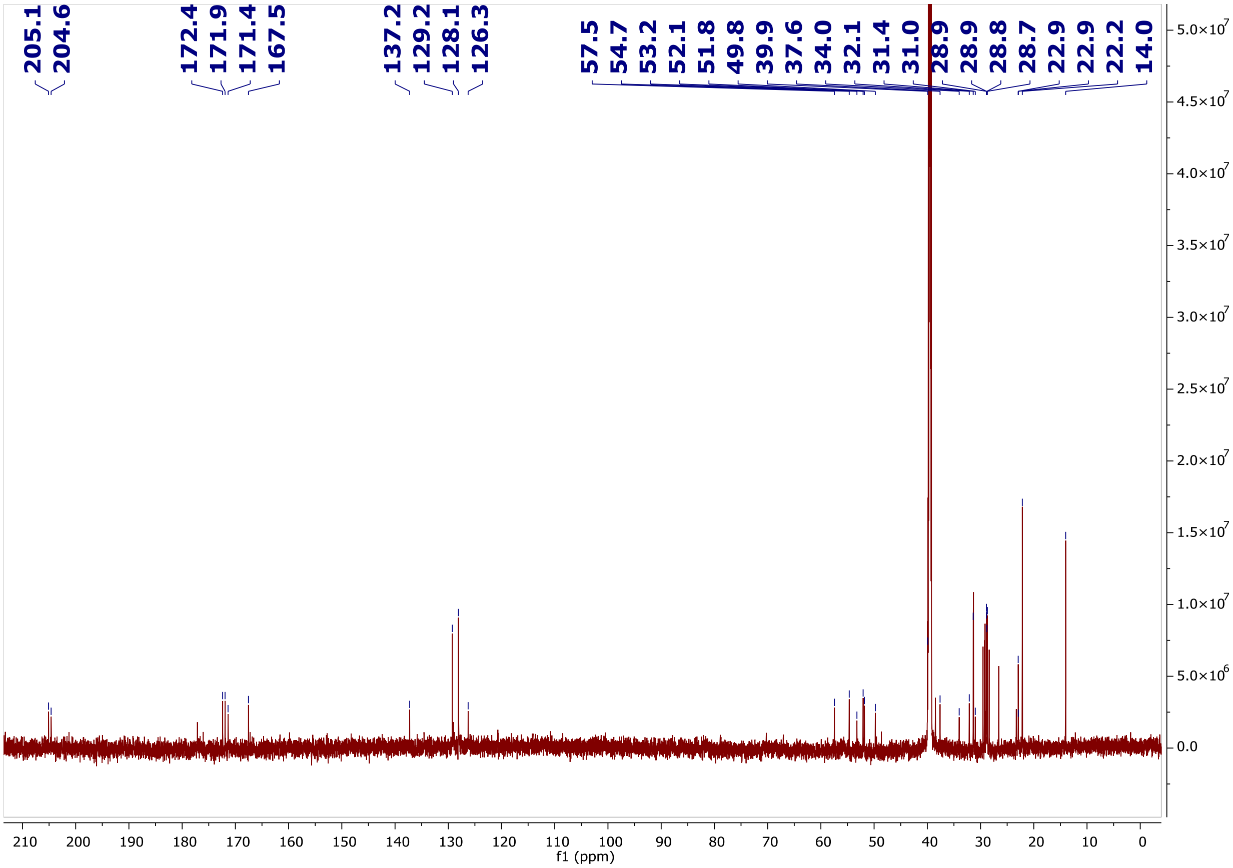

Figure S6b. ^{13}C NMR spectrum of Compound **1a**, DMSO-d$_6$ at 225MHz.

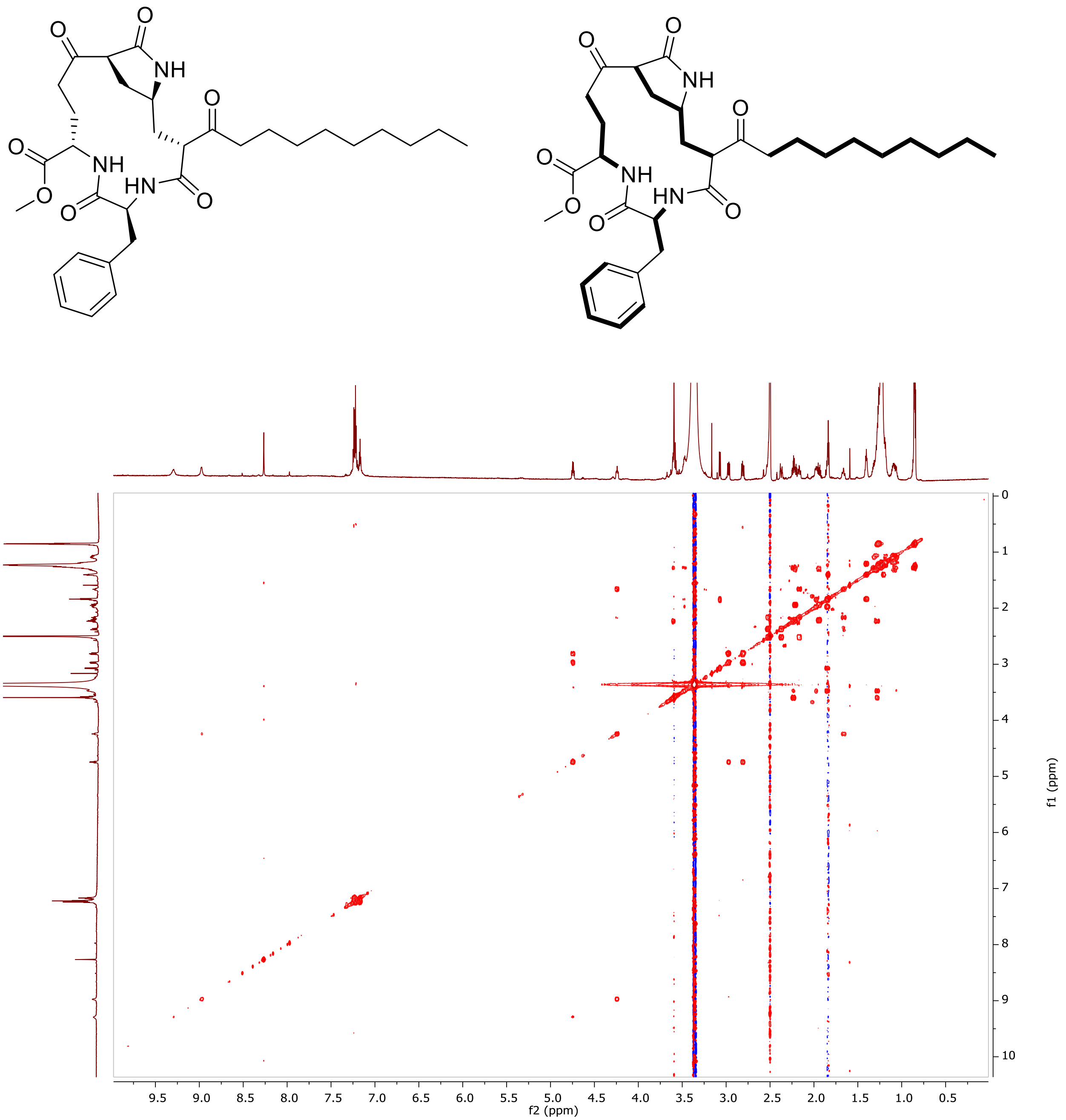

Figure S6c: ^{1}H-^{1}H COSY spectrum of Compound **1a**, DMSO-d_6 at 900MHz. Observed key COSY relations are shown above.

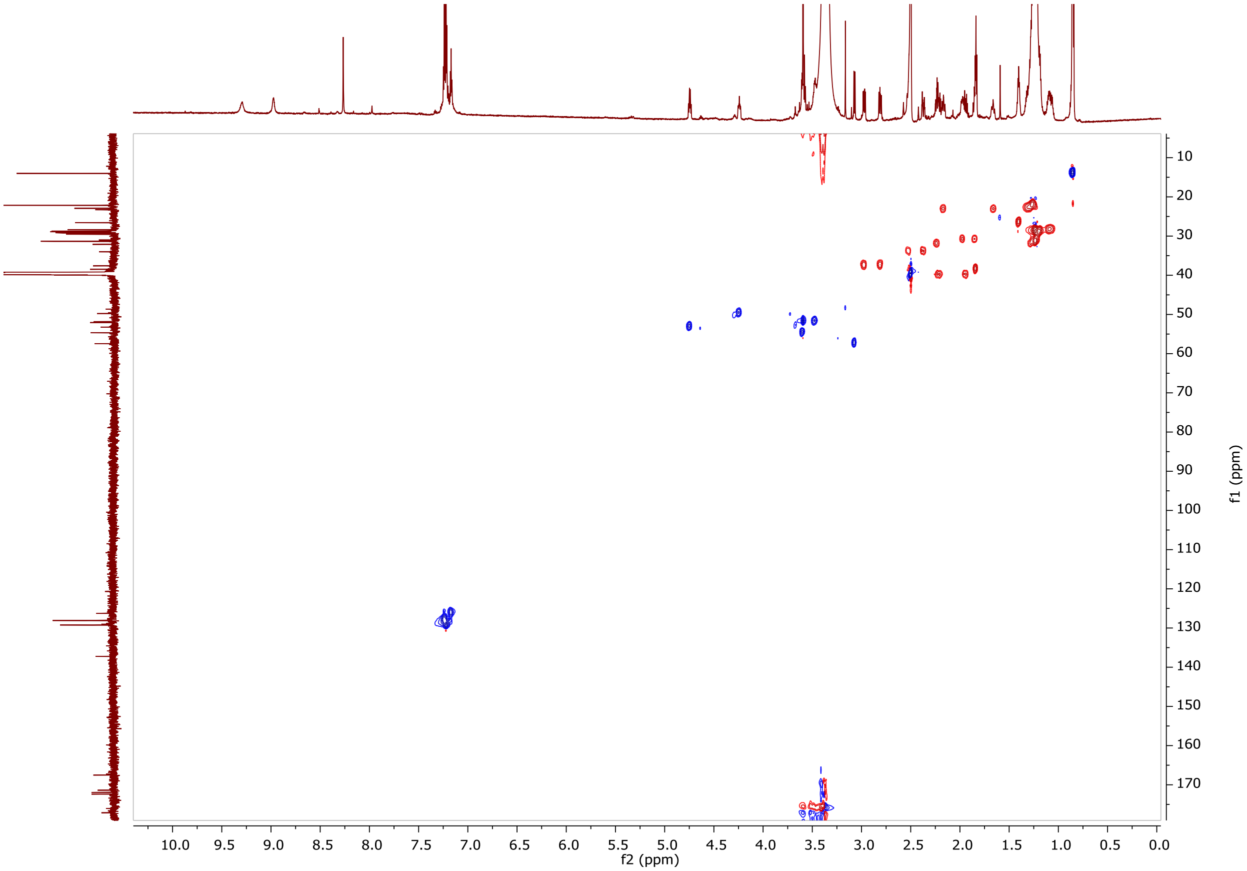

Figure S6d: ^{1}H-^{13}C HSQC spectrum of Compound **1a**, DMSO-d_6 at 900MHz.

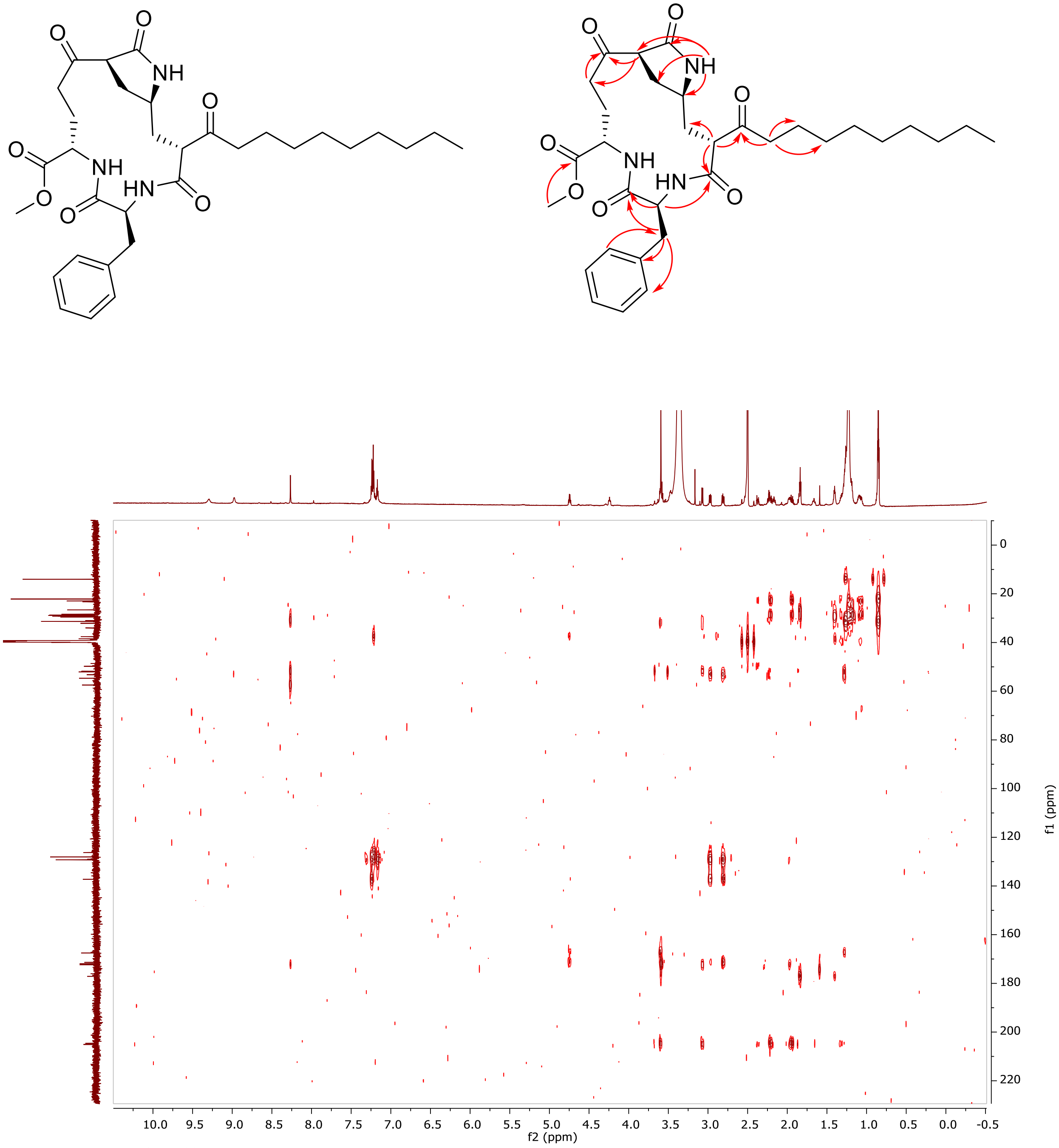

Figure S6e: ^{1}H-^{13}C HMBC spectrum of Compound **1a**, DMSO-d_6 at 900MHz. Observed key HMBC correlations are highlighted above.

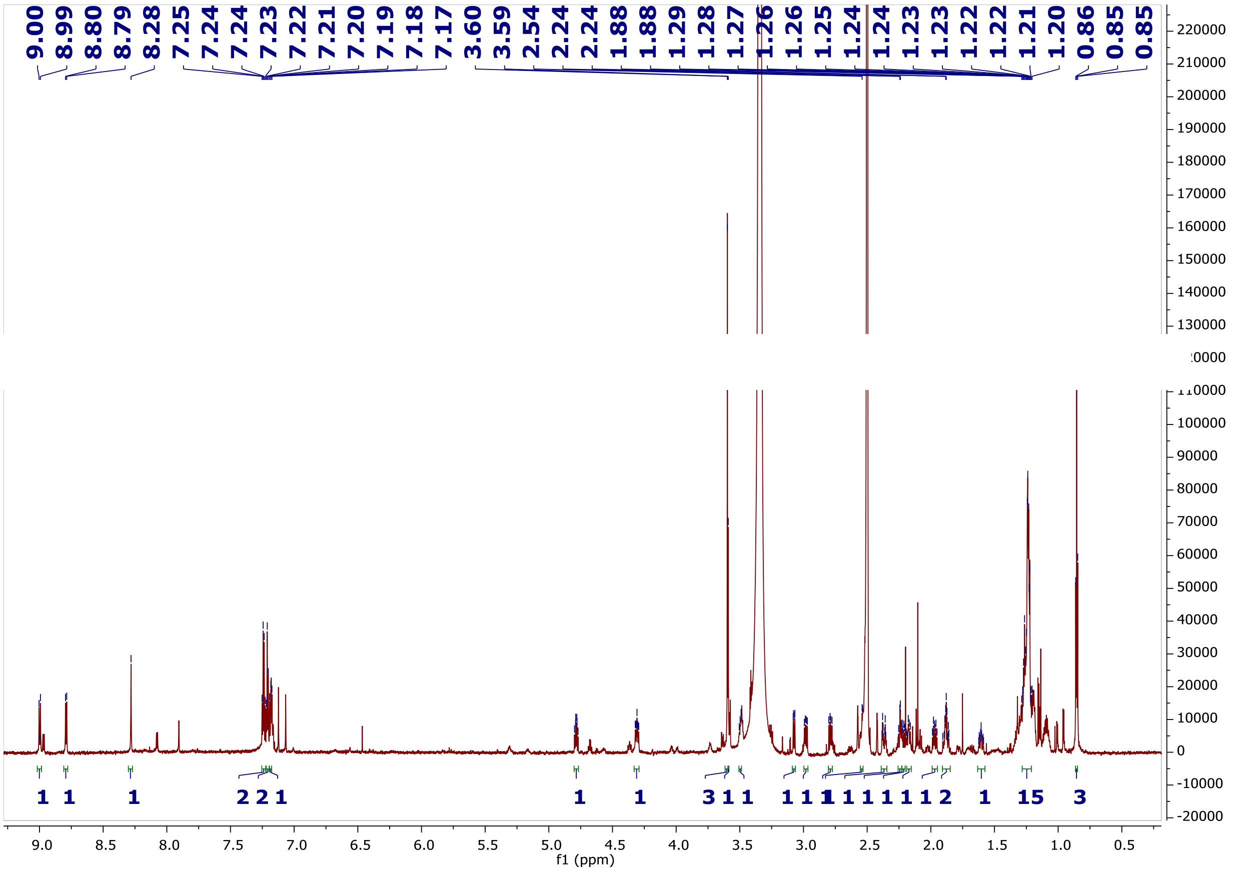

Figure S7a: ^{1}H NMR spectrum of Compound **1b**, DMSO-d_6 at 900MHz.

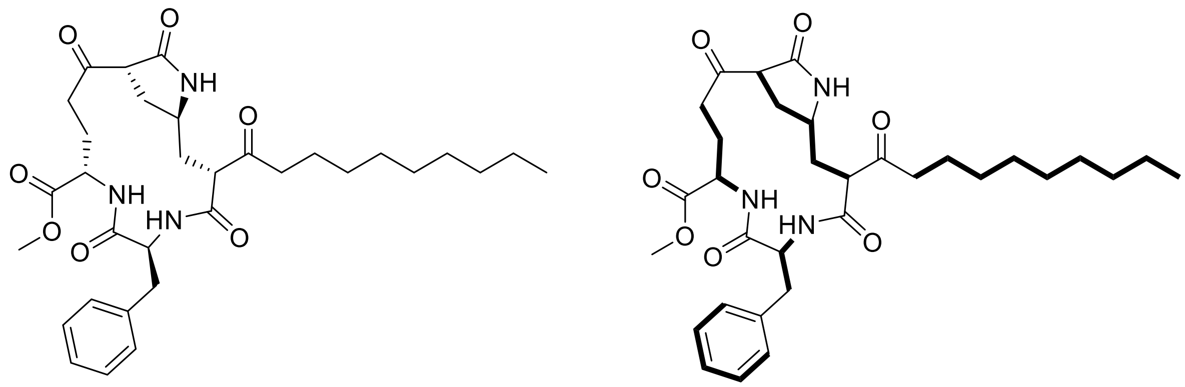

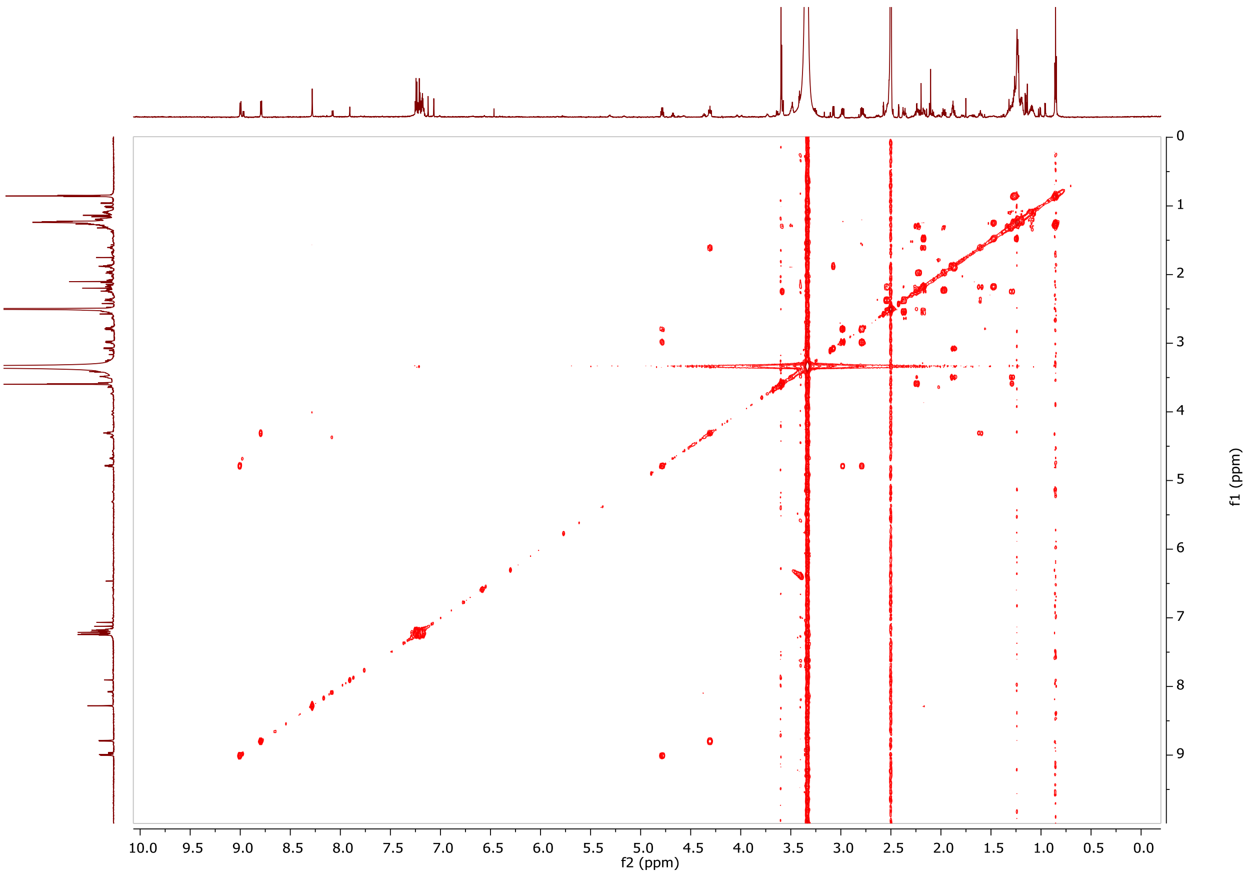

Figure S7b: ^{1}H-^{1}H COSY spectrum of Compound **1b**, DMSO-d_6 at 900MHz. Observed key COSY correlations are shown above.

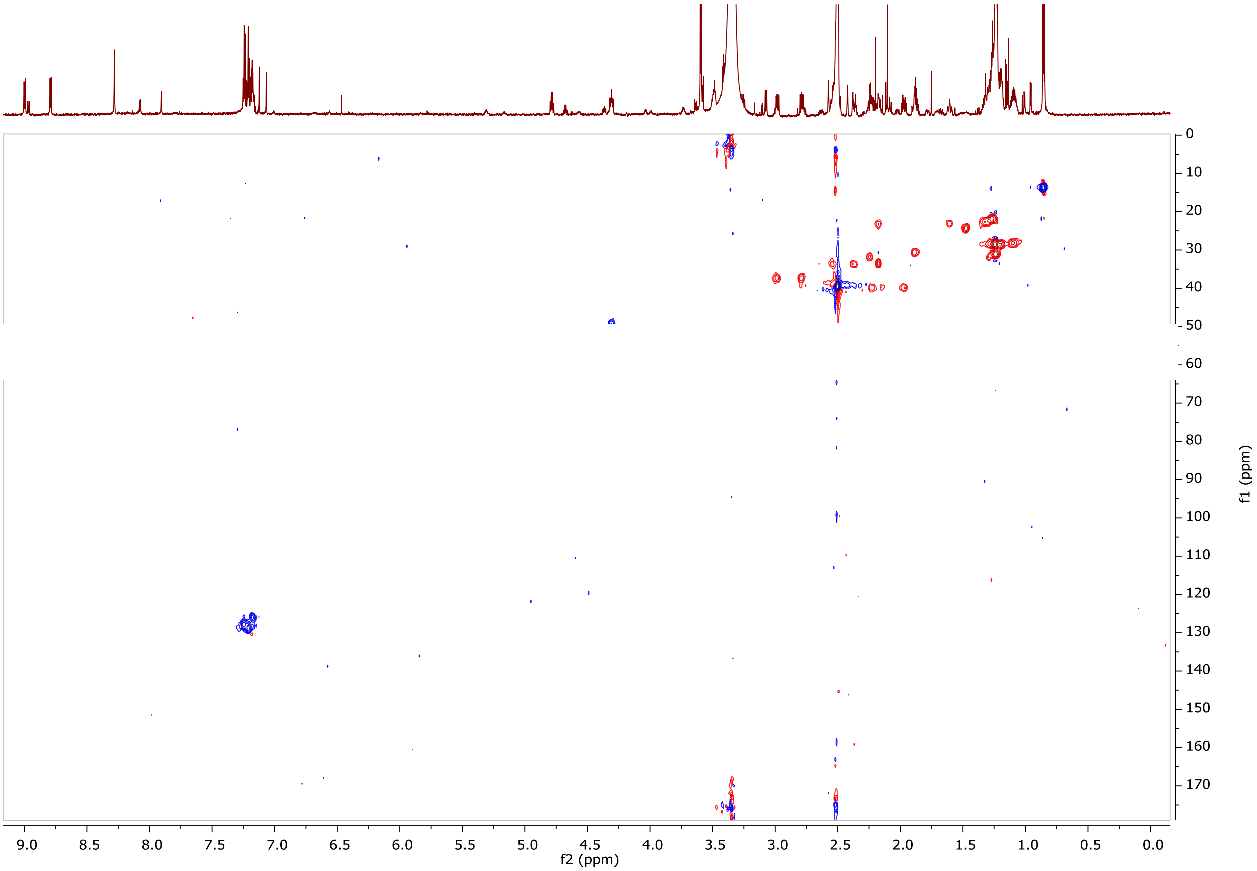

Figure S7c: ^{1}H-^{13}C HSQC spectrum of Compound **1b**, DMSO-d_6 at 900MHz.

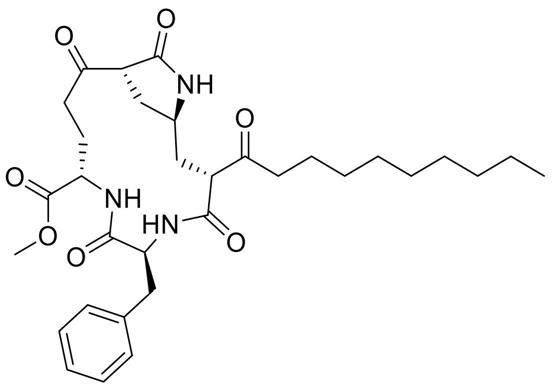

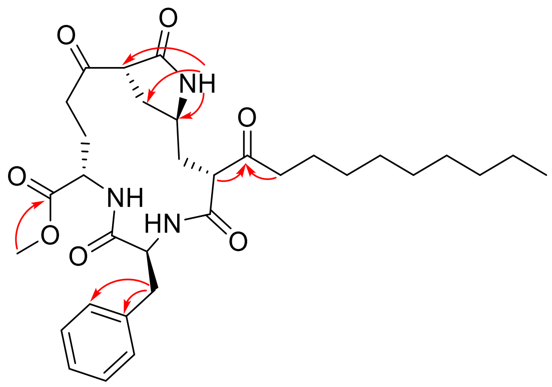

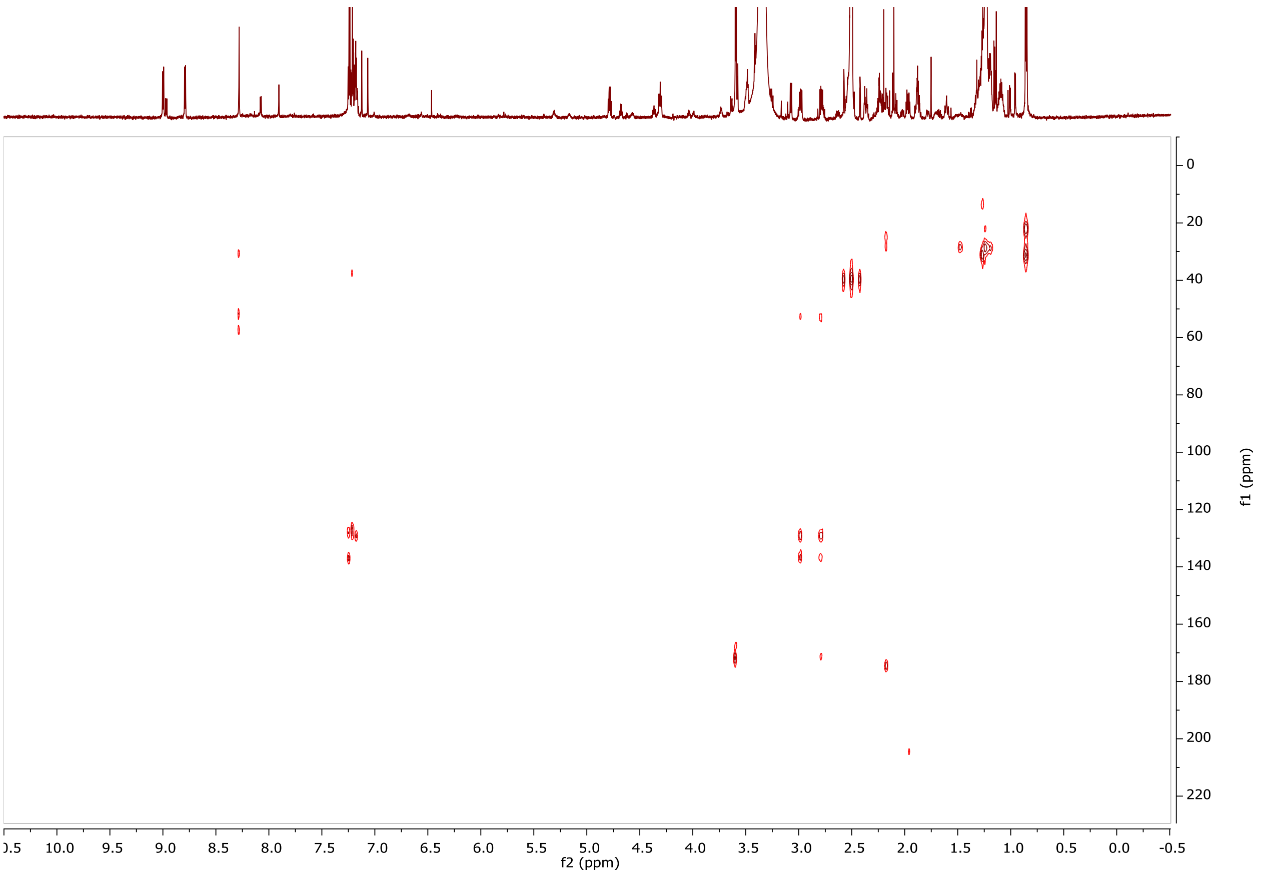

Figure S7d: ^{1}H-^{13}C HMBC spectrum of Compound **1b**, DMSO-d_6 at 900MHz. Observed key HMBC correlations are shown above.

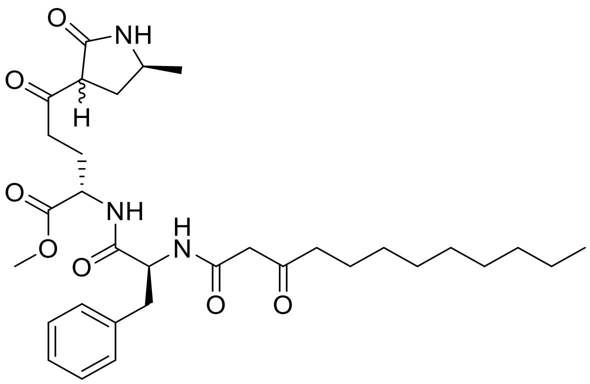

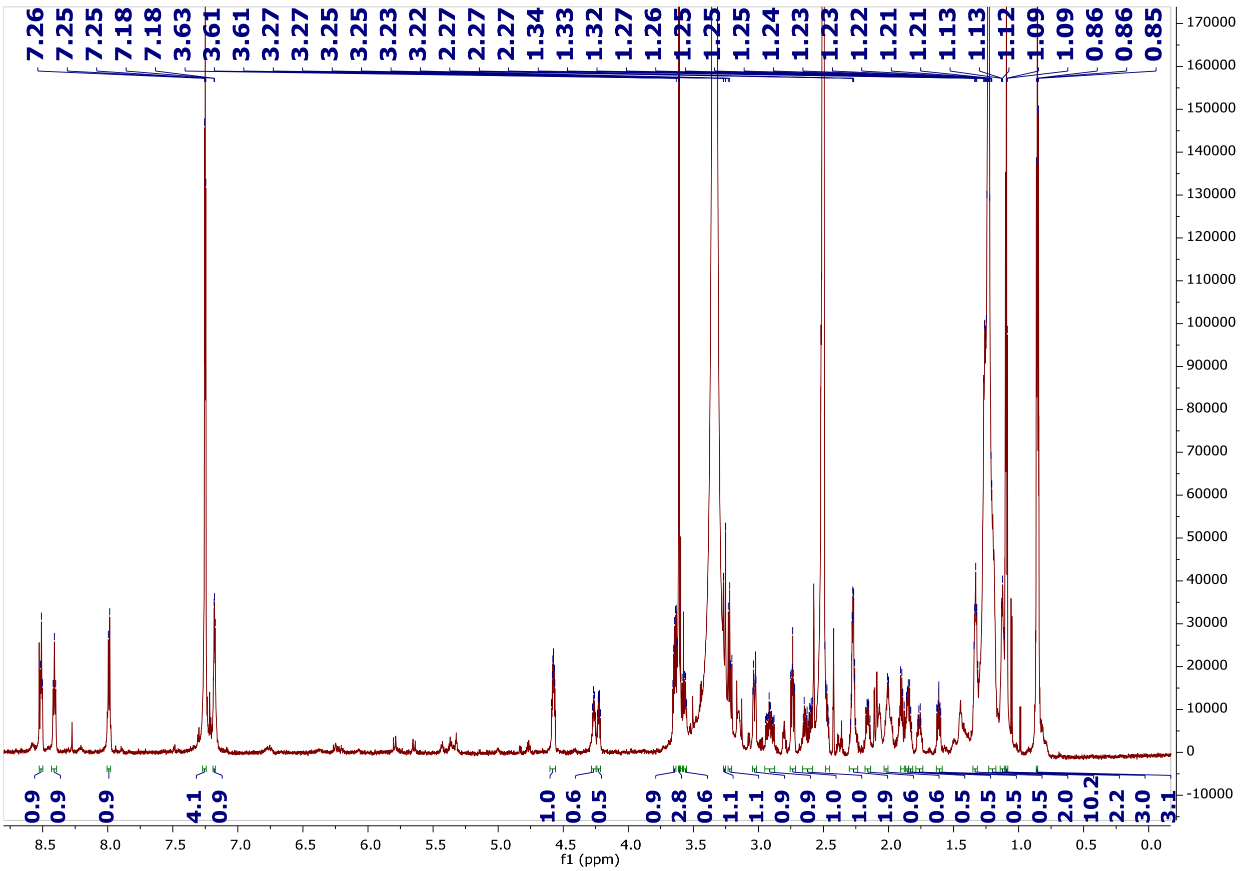

Figure S8a: ^{1}H NMR spectrum of Compound **2a/b**, DMSO-d_6 at 900MHz.

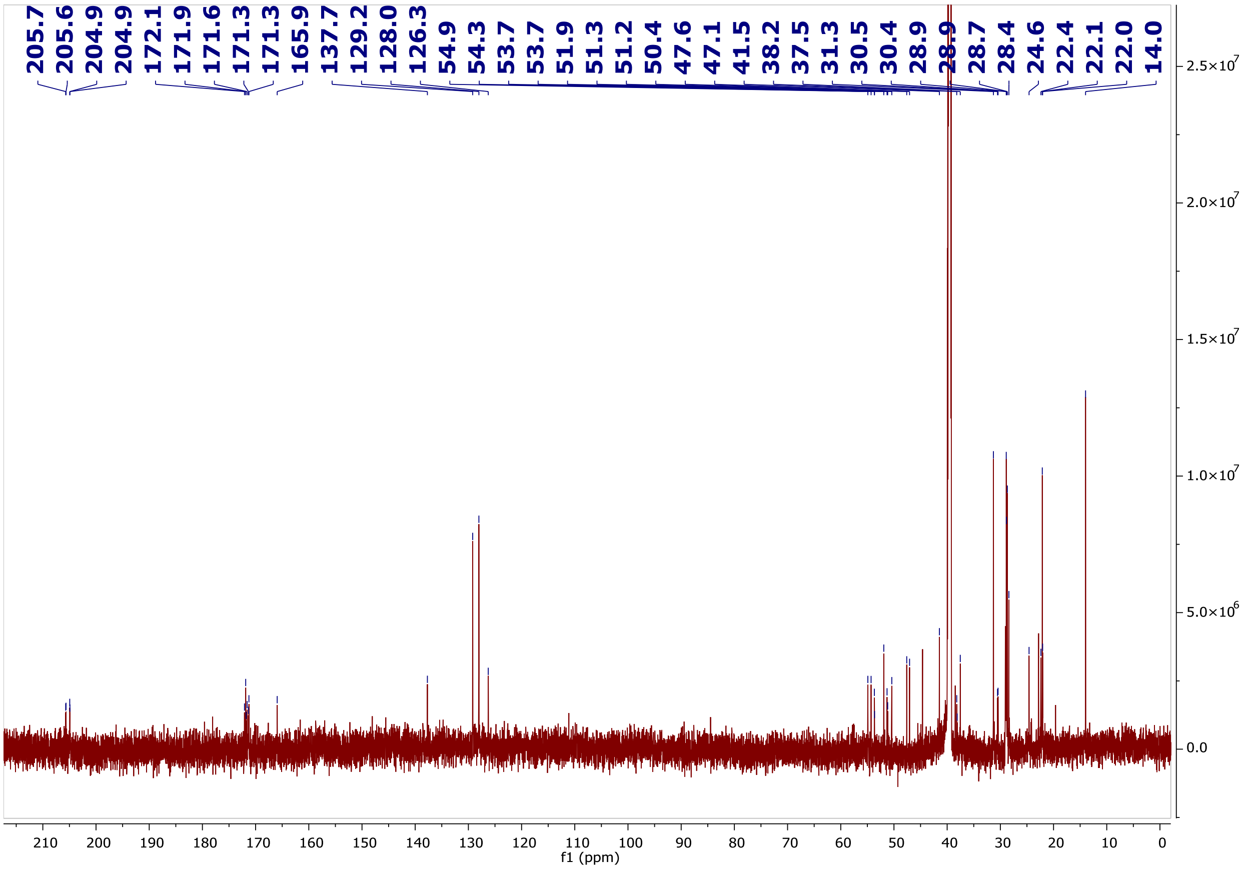

Figure S8b: ^{13}C NMR spectrum of Compound **2a/b**, DMSO-d_6 at 225MHz.

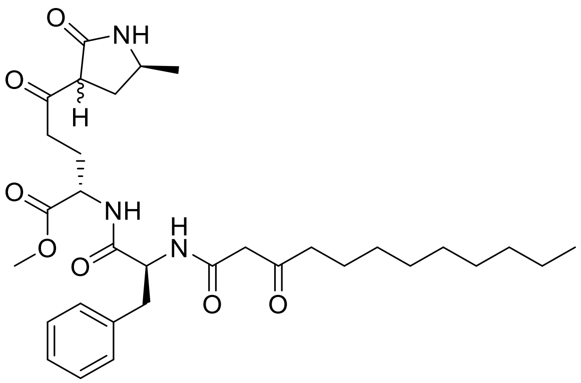

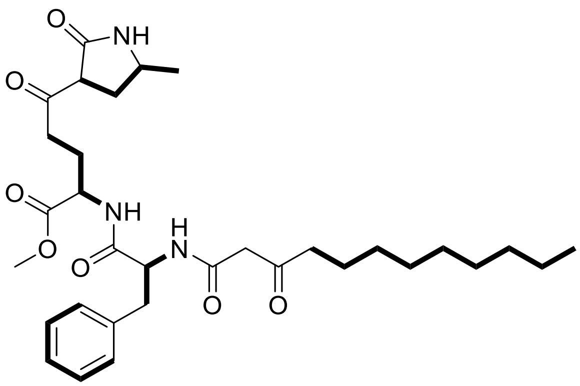

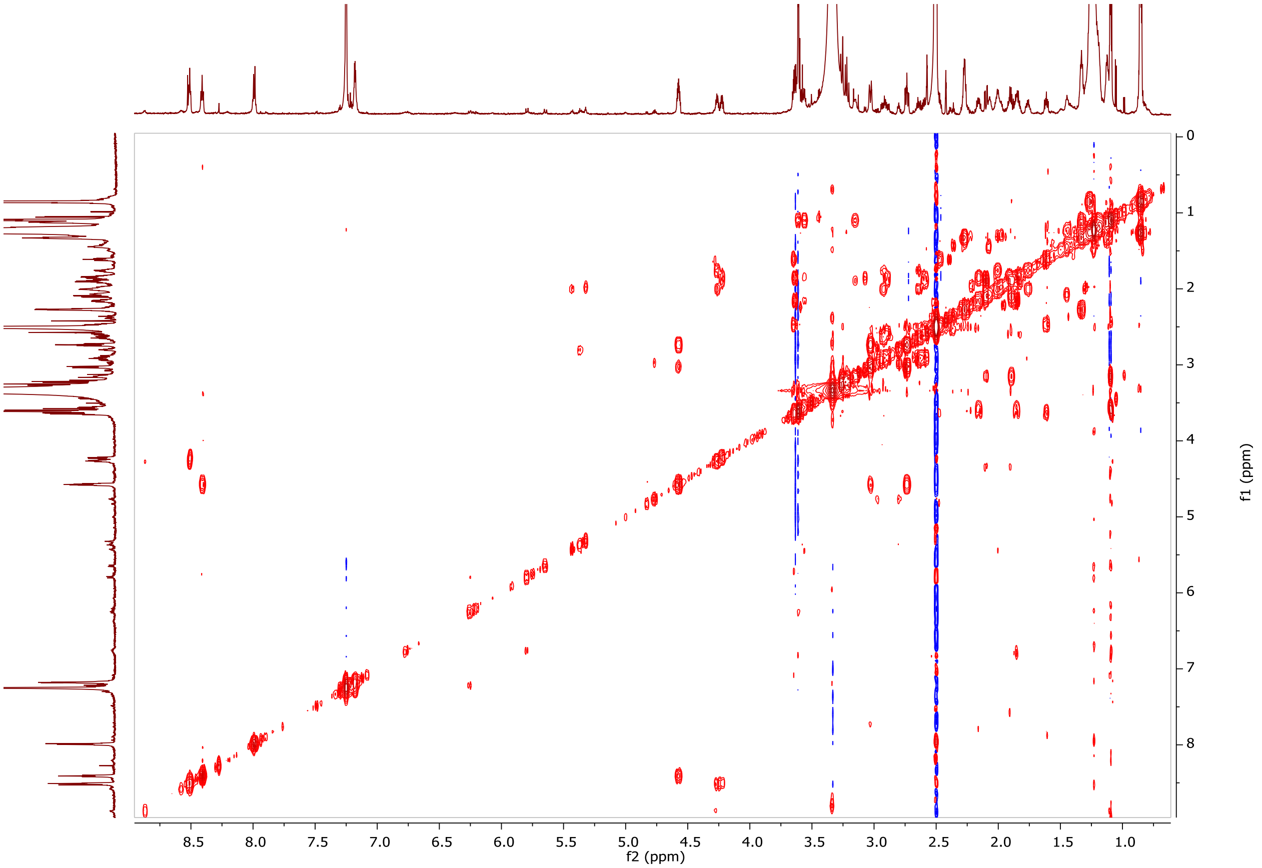

Figure S8c: ^{1}H-^{1}H COSY spectrum of Compound **2a/b**, DMSO-d$_6$ at 900MHz. Observed key COSY correlations are shown above.

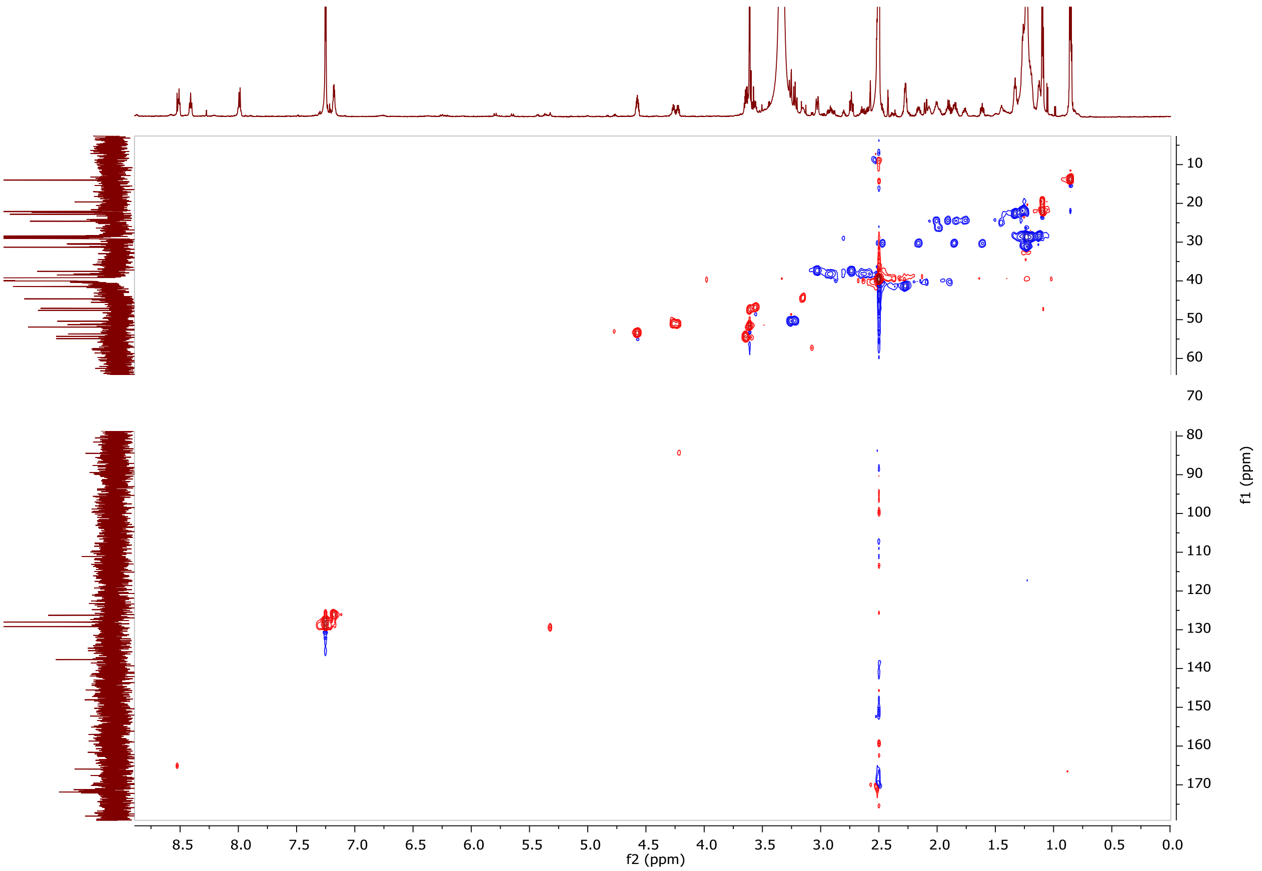

Figure S8d: ^{1}H-^{13}C HSQC spectrum of Compound **2a/b**, DMSO-d_6 at 900MHz.

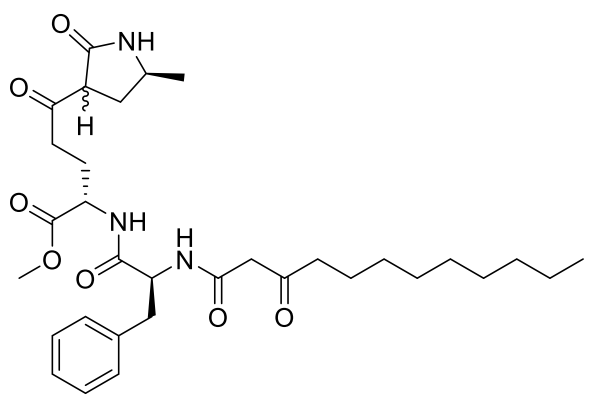

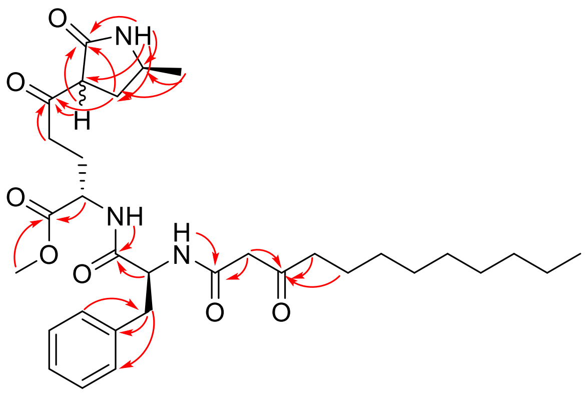

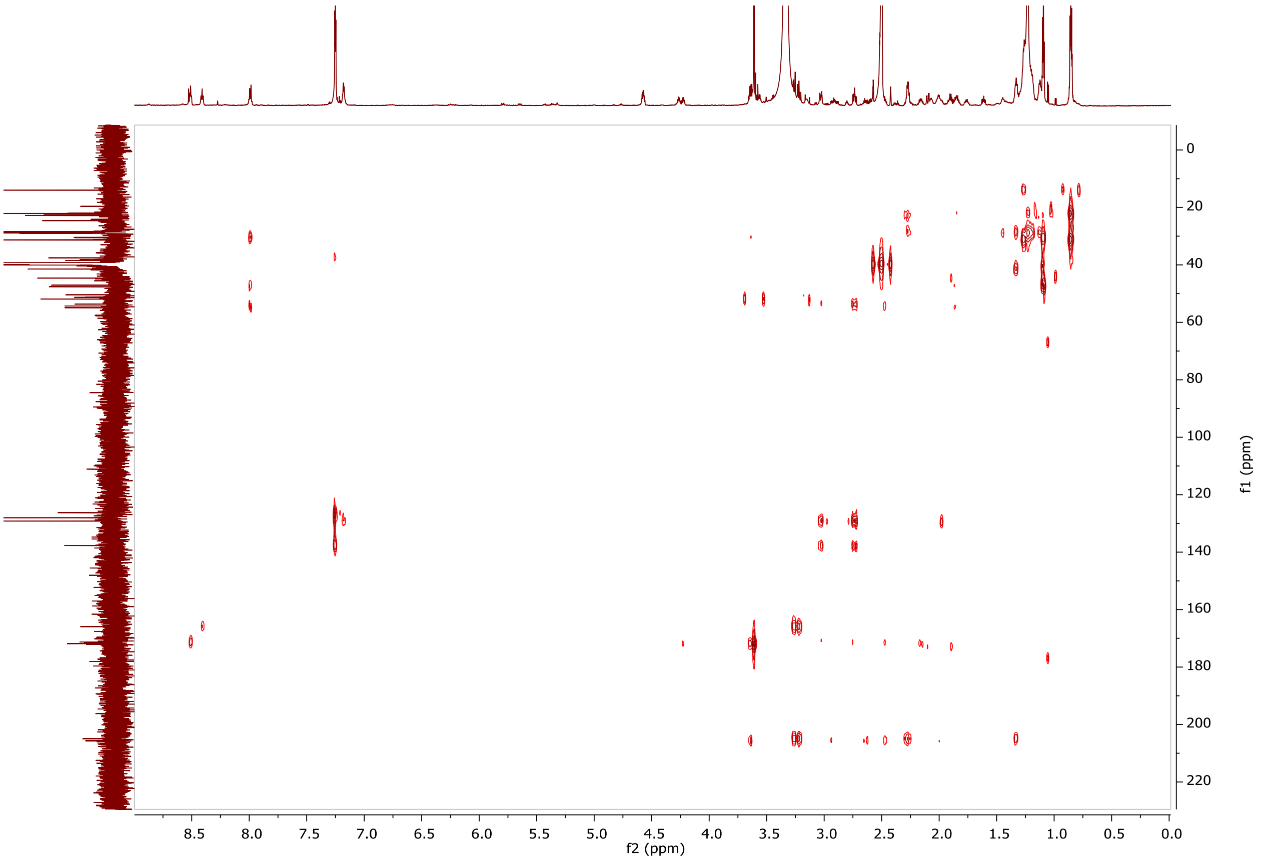

Figure S8e. ^{1}H-^{13}C HMBC spectrum of Compound **2a/b**, DMSO-d_6 at 900MHz. Observed key HMBC correlations are shown above.

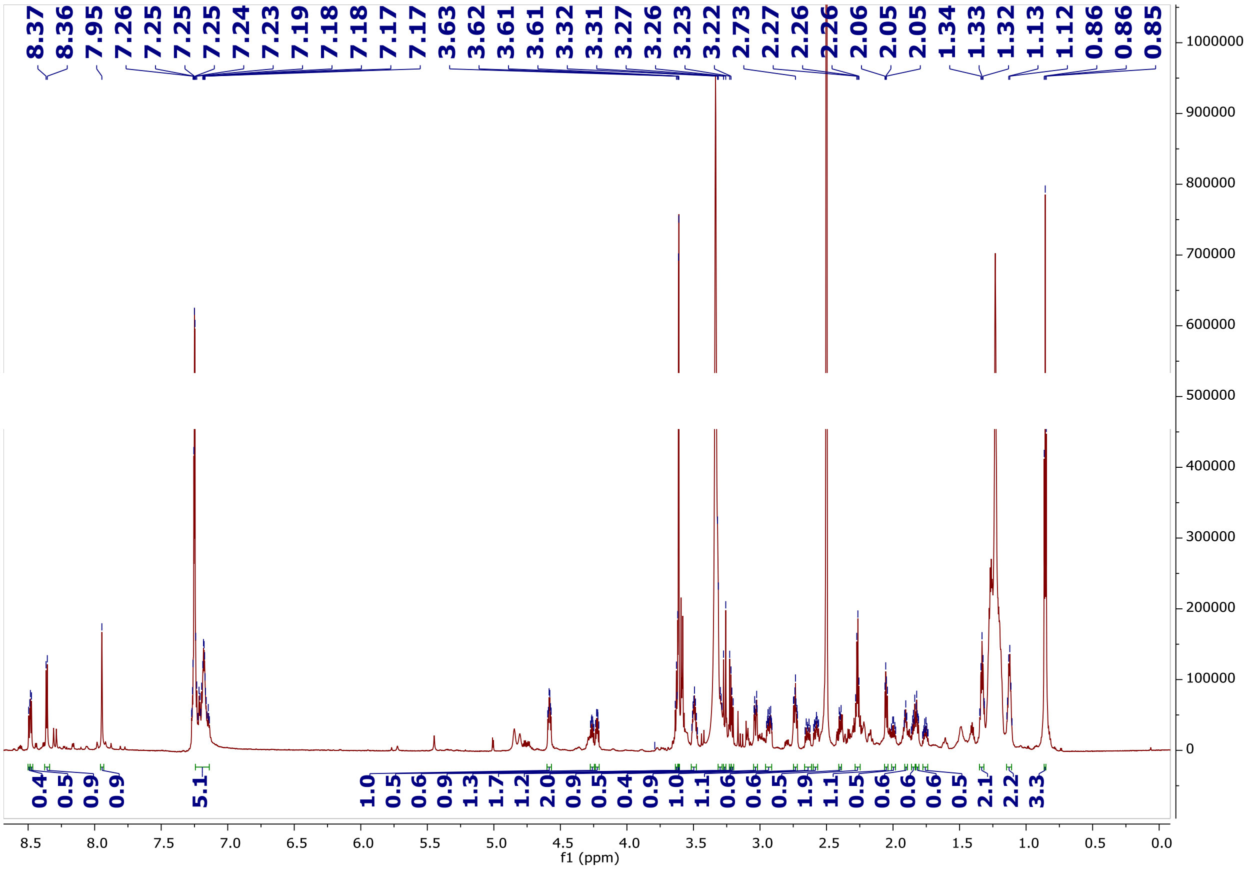

Figure S9a: ^{1}H NMR spectrum of Compound **3a/b**, DMSO-d_6 at 900MHz.

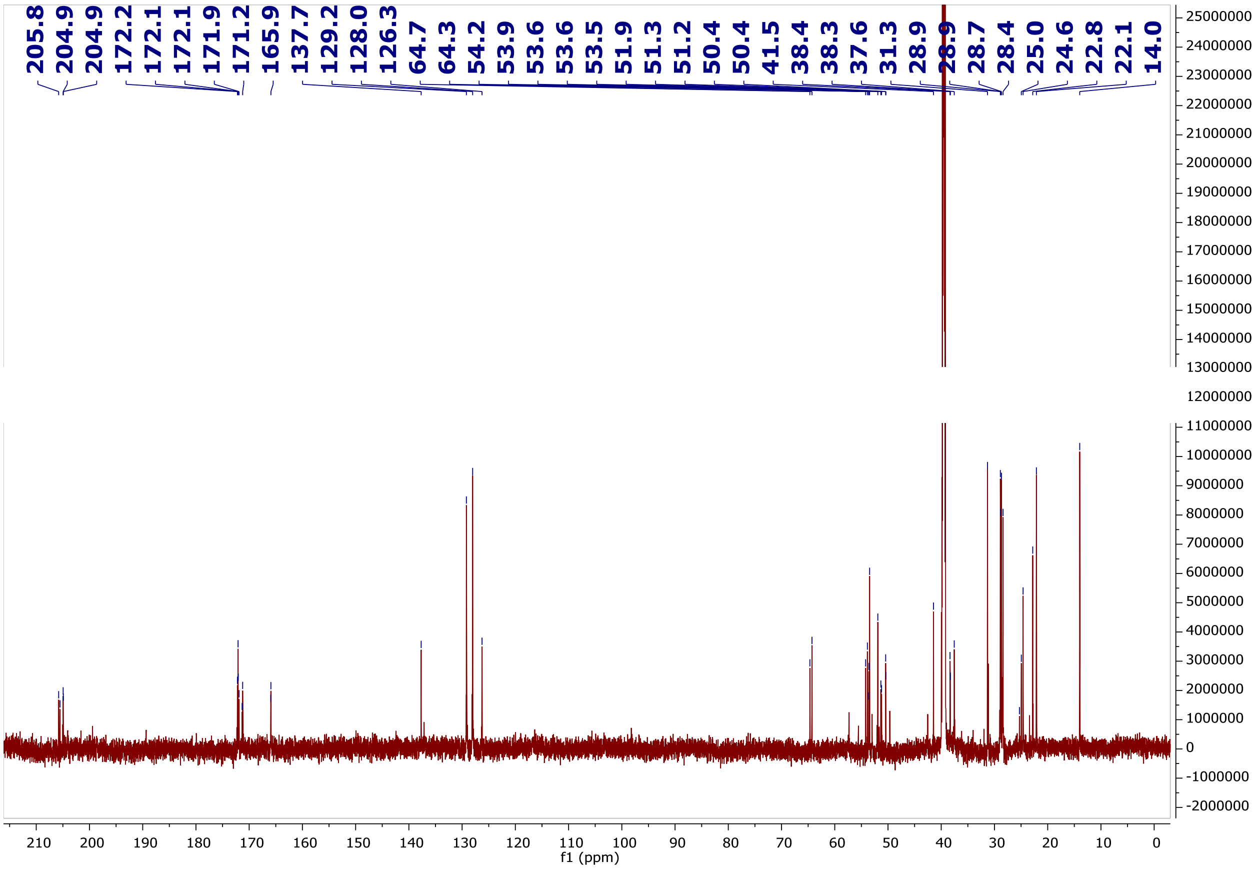

Figure S9b: ^{13}C NMR spectrum of Compound **3a/b**, DMSO-d_6 at 225MHz.

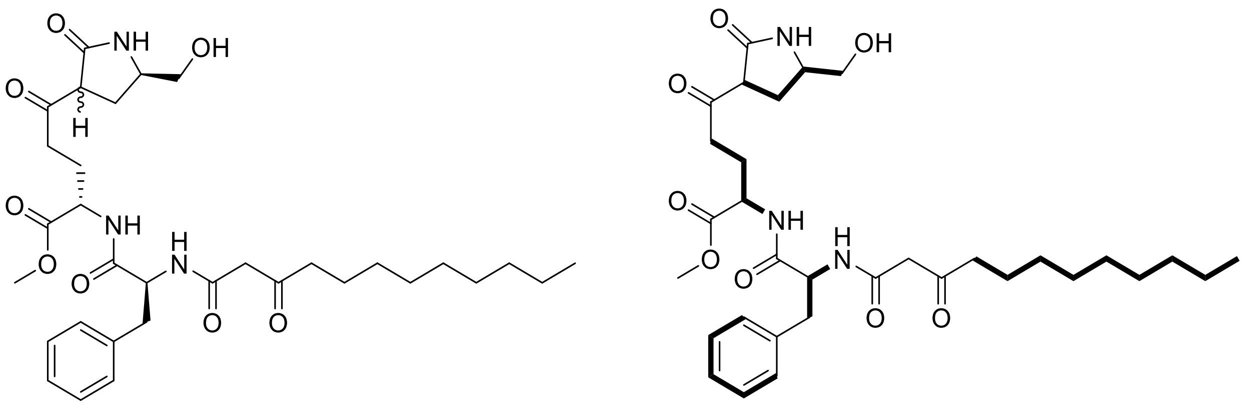

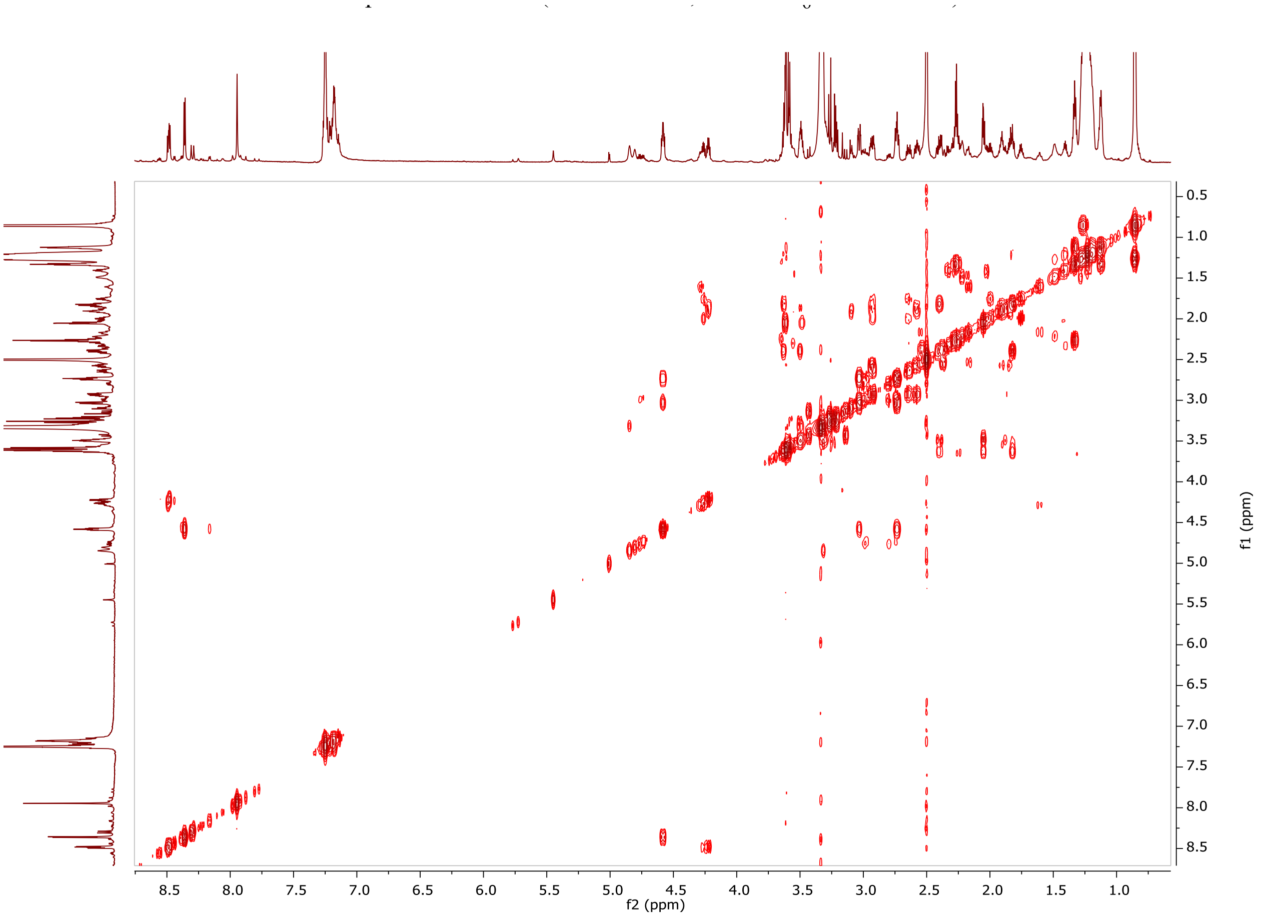

Figure S9c: ^{1}H-^{1}H COSY spectrum of Compound **3a/b**, DMSO-d_6 at 900MHz. Observed key COSY correlations are shown above.

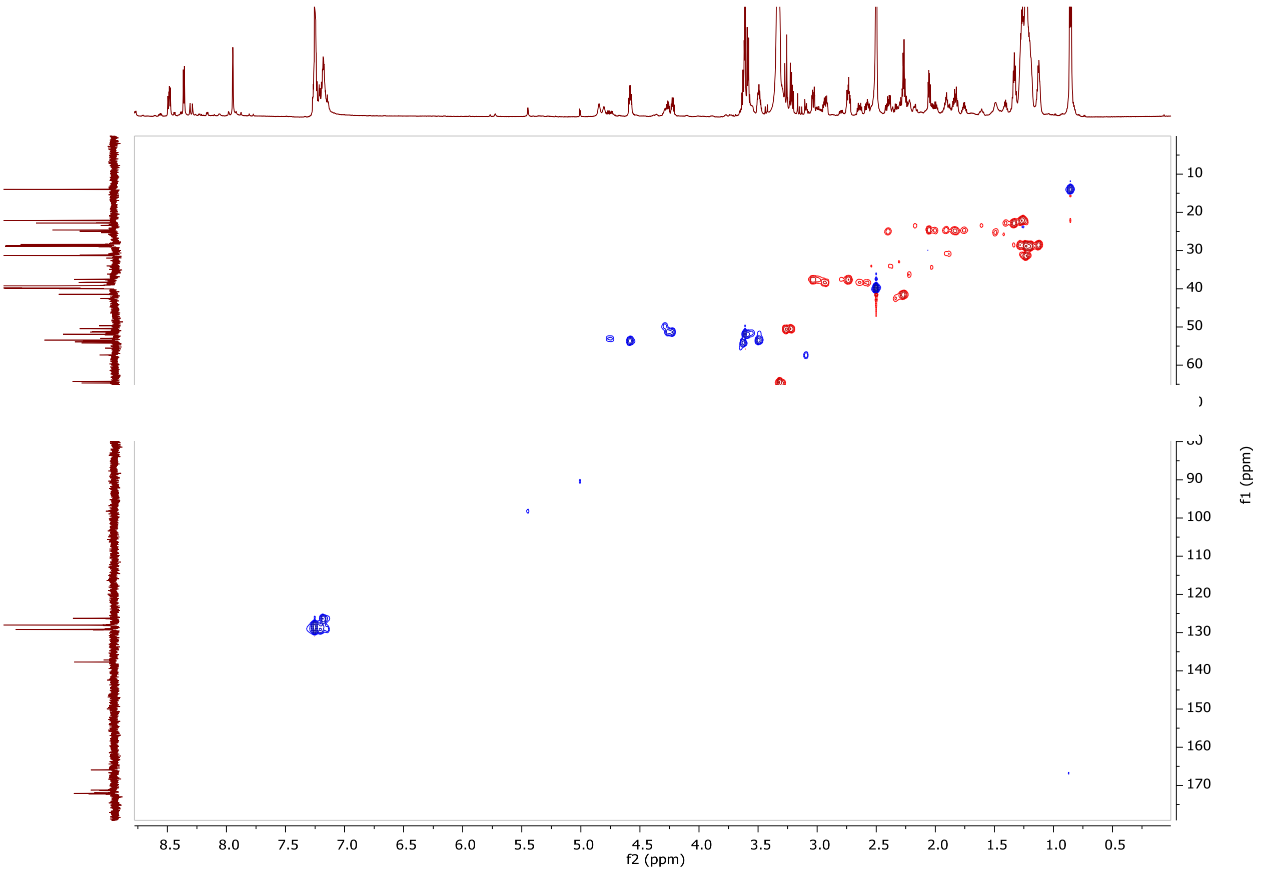

Figure S9d: ^{1}H-^{13}C HSQC spectrum of Compound **3a/b**, DMSO-d_6 at 900MHz.

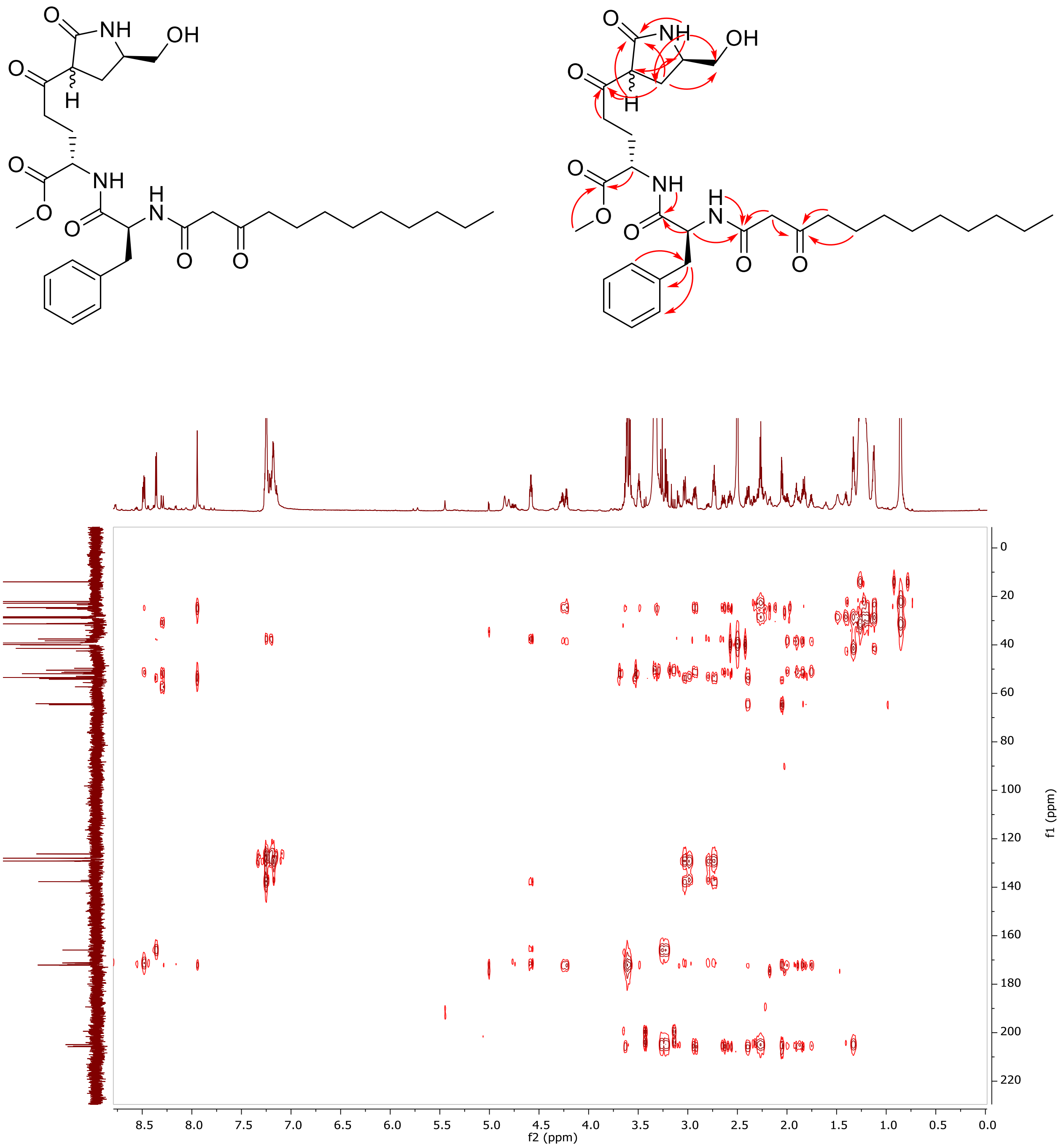

Figure S9e: ^{1}H-^{13}C HMBC spectrum of Compound **3a/b**, DMSO-d$_6$ at 900MHz. Observed key HMBC correlations are shown above.

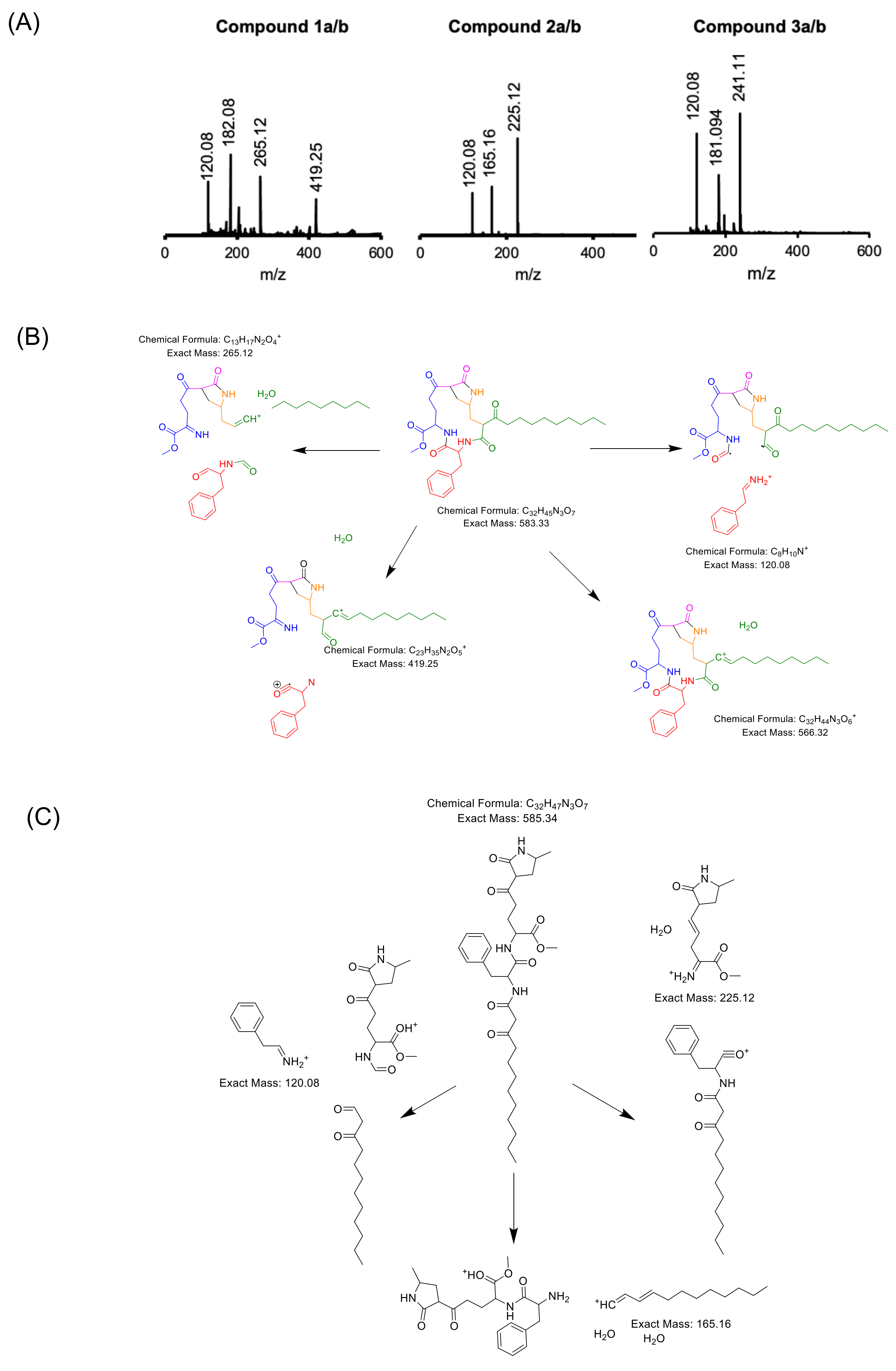
(A)
Compound 1a/b
120.08
182.08
265.12
419.25
Compound 2a/b
120.08
165.16
225.12
Compound 3a/b
120.08
181.094
241.11
0
200
400
600
m/z
(B)
Chemical Formula: C13H17N2O4+
Exact Mass: 265.12
H2O
Chemical Formula: C32H45N3O7
Exact Mass: 583.33
Chemical Formula: C8H10N+
Exact Mass: 120.08
Chemical Formula: C23H35N2O5+
Exact Mass: 419.25
Chemical Formula: C32H44N3O6+
Exact Mass: 566.32
(C)
Chemical Formula: C32H47N3O7
Exact Mass: 585.34
Exact Mass: 225.12
Exact Mass: 120.08
Exact Mass: 165.16
H2O

(D)

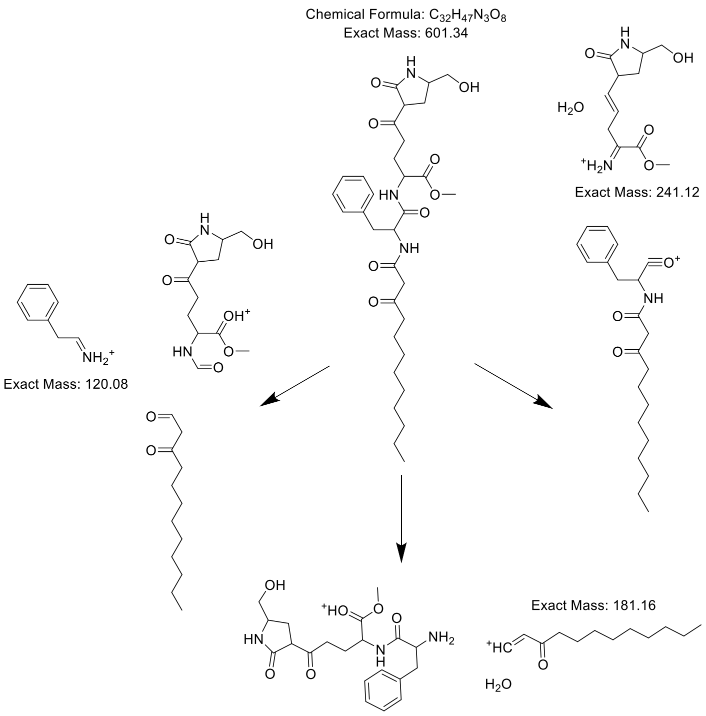

Figure S10: Predicted MS2 fragmentation pattern for mutanoclumpin molecules. A) MS/MS of compounds **1a/b**, **2a/b**, and **3a/b** with collision energies of 45.0eV at their respective retention times: 26.885min, 27.336min, and 24.609min, respectively. For B) MC-584 (**1**), C) MC-586 (**2**), and D) MC-602 (**3**), MS2 peaks were assigned utilizing CFM-ID to assist with fragmentation predictions. B) For **1**, the (*m/z* 419.25) peak was identified as a y and b amide bond break around the phenylalanine and a loss of water from the fatty acid chain. This water loss is also observed in the (*m/z* 566.32) fragment. The (*m/z* 419.25) peak is useful because the mass loss (-165.1), can only occur following a macrocyclization. Another noted peak included the a and y amide bond breaks around the phenylalanine resulting an (*m/z* 120.08) fragment. This is a common phenylalanine peptide fragment. Lastly, (*m/z* 265.12) is expected to be an a and b amide bond break around the phenylalanine as well as a loss of water. C) For **2**, the key (*m/z* 120.08) metabolite remains. There is an (*m/z* 165.16) fragment that is expected to be a phenylalanine b amide fragmentation with two water losses. Otherwise, the fragmentation resembles the MC-602 linear characteristic fragment, containing an (*m/z* 241.12) fragment with a C-OH group removed (*m/z* 225.12). D) For **3**, the key (*m/z* 120.08) metabolite found in the macrocyclized molecule reasonably appears as a linear fragment. This key fragment can serve to provide some confidence in late assembly line masses determined to be related to *mcg*, such as compound **4**. There is a fragment, (*m/z* 181.16), which occurs with a phenylalanine b amide fragmentation and a water loss. Lastly there is an (*m/z* 241.12) mass which occurs after a phenylalanine y fragmentation and a water loss. The (*m/z* 241.12) mass is of relevance because it is only expected to occur in linear molecules.

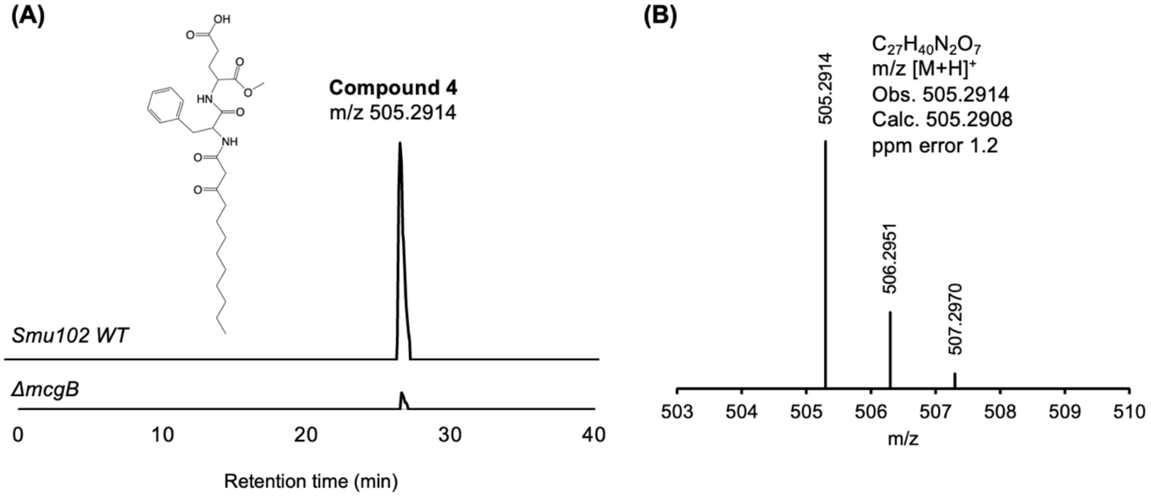

Figure S11: Identification of shunt metabolite 4. A) EICs showing production of compound **4** (*m/z* 505.2914 $[M+H]^+$) in Smu102 and Δ*mcgB*. B) HRMS of **4**.

Figure S12-(A) Table:

Gene name	Locus tag	Proposed function	Closest homolog	%ID/%S	Necessary for biosynthesis of 1?	Evidence
mcgR	SMU102_RS08310	ComR-like transcriptional regulator	XRE family transcriptional regulator (*S. orisasini*)	89/95	No	Bioinformatic
mcgA	SMU102_RS08305	Hybrid NRPS/PKS	Hybrid NRPS/PKS (*S. orisasini*)	88/94	Yes	Bioinformatic
mcgB	SMU102_RS08300	PKS	Type I PKS (*S. orisasini*)	88/94	Yes	Knockout
mcgC	SMU102_RS08295	Hybrid NRPS/PKS	NRPS (*S. orisasini*)	88/94	Yes	Bioinformatic
mcgD	SMU102_RS08290	SAM-dependent methyltransferase	SAM-dependent methyltransferase (*S. orisasini*)	92/96	Yes	Knockout
mcgE	SMU102_RS08285	Transporter	Macrolide transporter (*S. chenjunshii*)	89/95	No	Bioinformatic
mcgF	SMU102_RS08280	Transporter	ABC transporter ATP-binding protein (*S. orisasini*)	93/95	No	Bioinformatic
mcgG	SMU102_RS08275	Transporter	ABC transporter permease (*S. pantholopis*)	94/95	No	Bioinformatic
mcgH	SMU102_RS08270	Transporter	ABC transporter permease (*S. pantholopis*)	89/96	No	Bioinformatic
mcgI	SMU102_RS08265	Fatty acyl-AMP ligase	AMP-binding protein (*S. pantholopis*)	93/96	Yes	Bioinformatic
mcgJ	SMU102_RS08260	Transporter	ABC transporter ATP-binding protein (*S. orisasini*)	92/97	No	Bioinformatic
mcgK	SMU102_RS08255	Transporter	ABC transporter permease (*S. orisasini*)	88/93	No	Bioinformatic
mcgL	SMU102_RS08250	4'-PPTase	4'-phosphopantetheinyltransferase (*S. pantholopis*)	85/93	No	Bioinformatic
mcgM	SMU102_RS08245	Type II thioesterase	Thioesterase domain-containg protein (*S. orisasini*)	94/97	No	Knockout
mcgN	SMU102_RS00810	*S*-adenosylmethionine synthetase	Methionine adenosyltransferase (*S. troglodytae*)	98/98	No	Knockout
mcgO	SMU102_RS00270	SDR family oxidoreductase	SDR family oxidoreductase (*S. orisasini*)	84/92	No	Knockout
mcgP	SMU102_RS10945	Pseudogene	Cysteine hydrolase (*S. troglodytae*)	100/100	No	Bioinformatic
1341	SMU102_01341	Unknown	YdcF family protein (*S. troglodytae*)	91/93	No	Knockout
1346	SMU102_01346	Metallo-hydrolase	MBL fold metallo-hydrolase (*S. troglodytae*)	94/96	No	Knockout
1351	SMU102_01351	Deaminase	tRNA adenosine deaminase TadA (*S. troglodytae*)	97/97	No	Knockout
1356	SMU102_01356	Unknown	Hypothetical protein	-	No	Knockout
ffs	-	4.5S RNA	-	-	No	Knockout

(B)

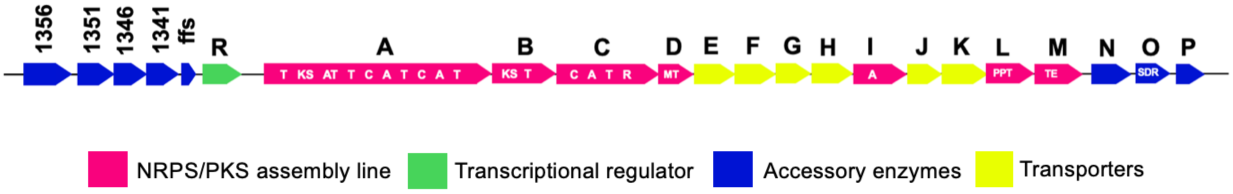

(C)

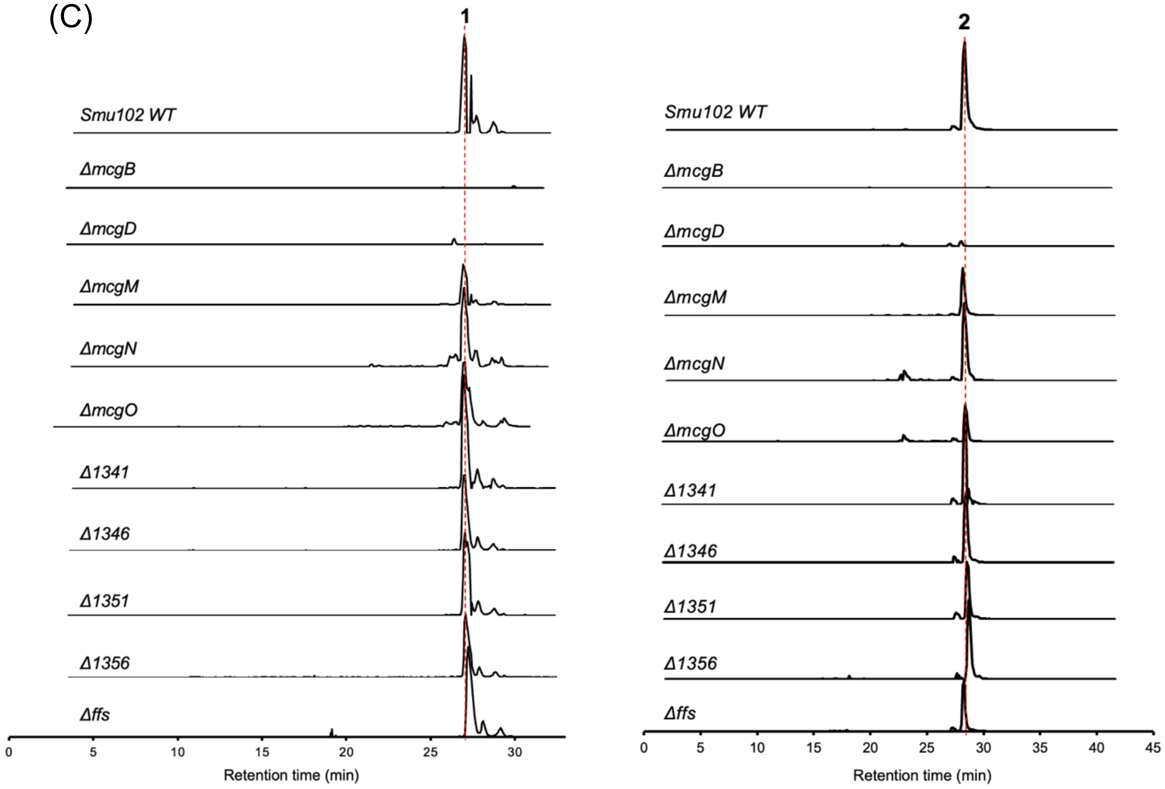

Figure S12: Production of 1 and 2 in Smu102 mutants. A) Table of gene functions encoded in *mcg.* Locus tags and names are given to *mcg* genes in *S. mutans* Smu102. Each gene includes its proposed function, closest homolog with percent identity and similarity (%ID/%S), and evidence for necessity in biosynthesis of **1**. B) Organization of the mutanoclumpin (*mcg*) biosynthetic gene cluster in Smu102, including upstream conserved region. Genes encoding NRPS or PKS are shown in pink, accessory enzymes are shown in blue, the transcriptional regulator is shown in green, and transporters are shown in yellow. A, adenylation; C, condensation; KS, ketosynthase; AT, acyltransferase; T, thiolation sequence of acyl- or peptidyl-carrier protein; R, reductase; MT, methyltransferase; PPT, phosphopantetheinyltransferase; TE, thioesterase; SDR, short chain dehydrogenase/reductase. C) EICs of HRMS for compounds **1** and **2** are shown for Smu102 mutants. EICs show relative counts versus retention time.

(A)

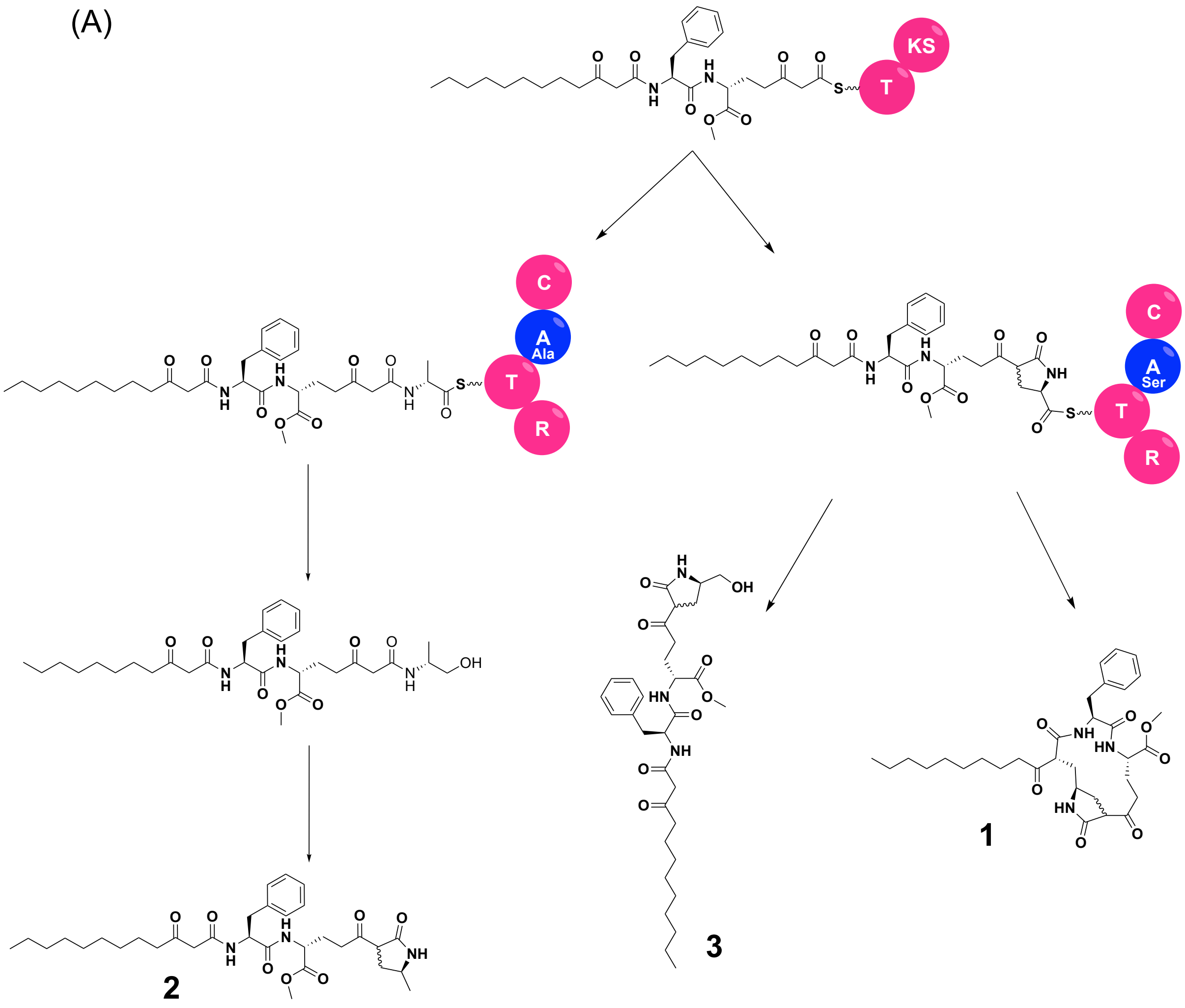
KS
T
C
A
Ala
T
R
C
A
Ser
T
R
1
2
3

(B)

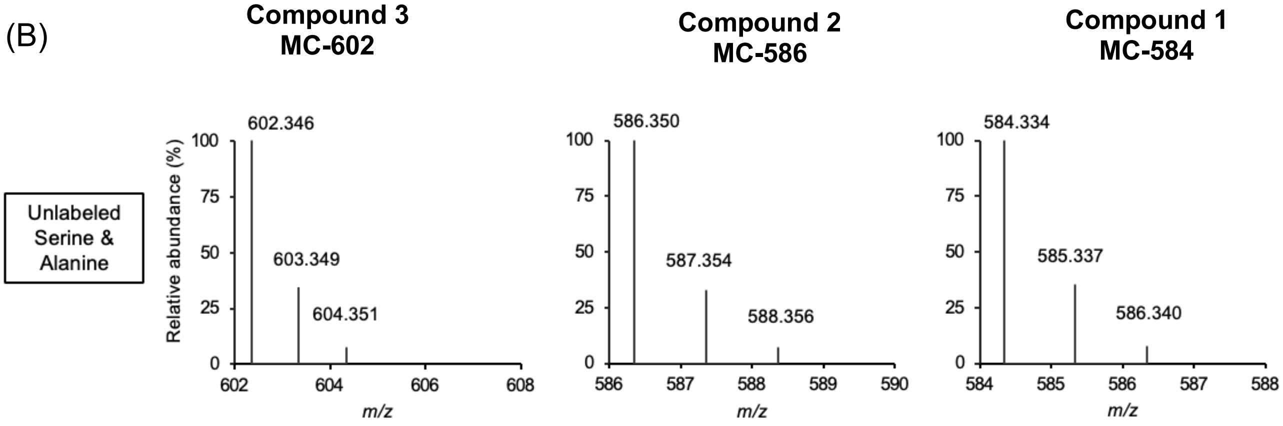
Compound 3
MC-602
Compound 2
MC-586
Compound 1
MC-584
Unlabeled Serine & Alanine
Relative abundance (%)
602.346
603.349
604.351
586.350
587.354
588.356
584.334
585.337
586.340
m/z

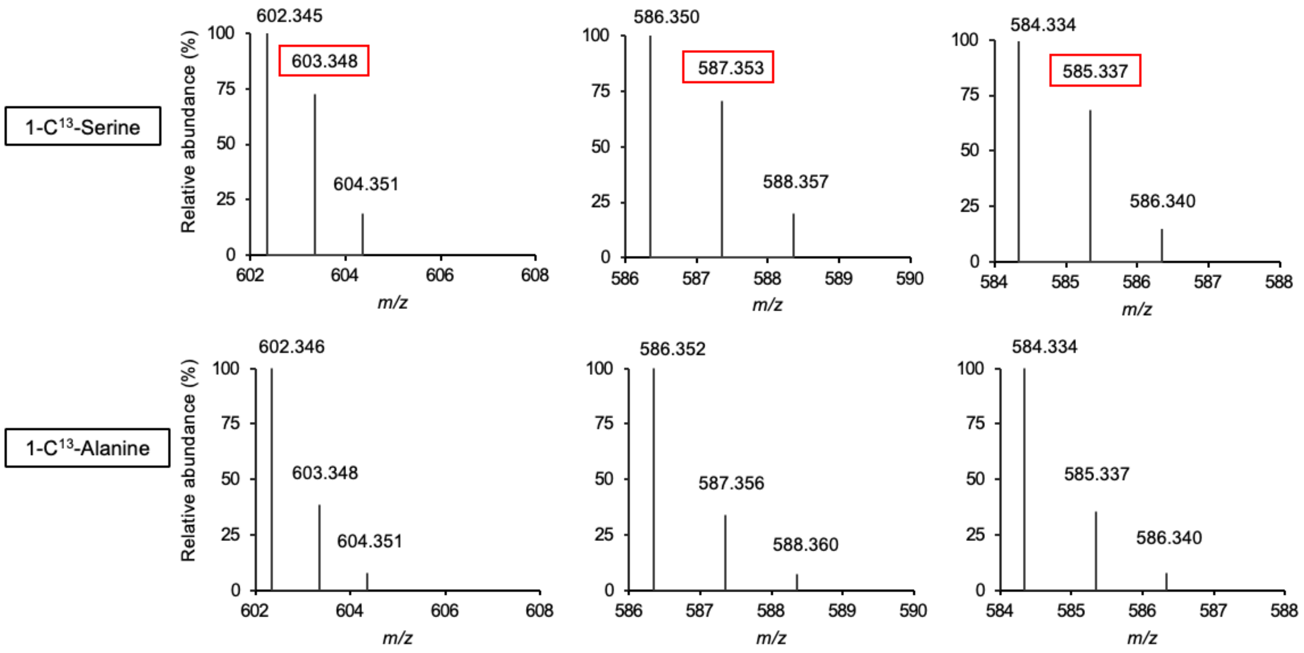

Figure S13: Hypothesized alanine and serine incorporation for divergent biosynthetic pathway. A) shows the predicted divergent biosynthetic scheme at the A domain in McgC to produce noncyclic **2** and macrocyclic **1** via serine or alanine incorporation. B) HRMS of compounds **3**, **2**, and **1**, respectively, under different labeled isotope feedings. The isotope labeled feedings include a control with no labeled serine or alanine, fed 1-^{13}C-Ser, and fed 1-^{13}C-Ala, respectively.

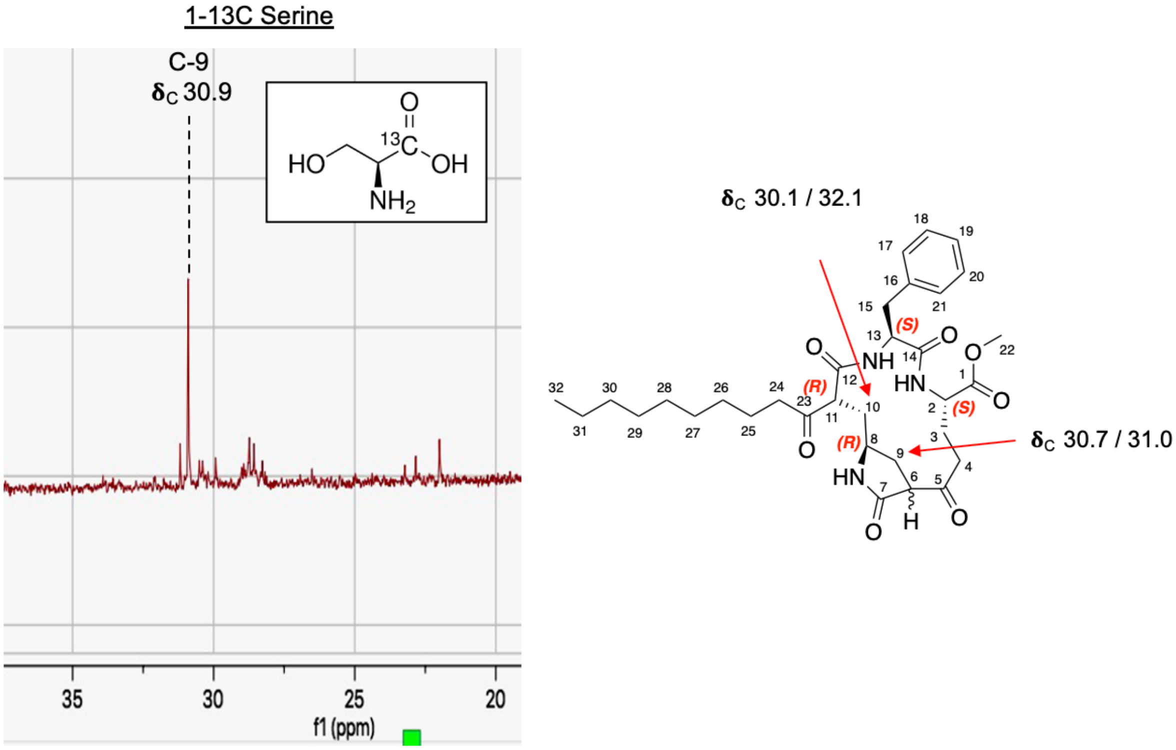

Figure S14: Enriched C-9 of 1 from 1-^{13}C-Ser feeding. ^{13}C NMR spectra shown with labeled carbon chemical shifts for C-9 and C-10 on **1**. ^{13}C NMR spectra was generated with ~0.7mg purified racemic **1a/b** from a culture enriched with 1-^{13}C-Ser. Chemical shifts on the structure are labeled 1b / 1a.

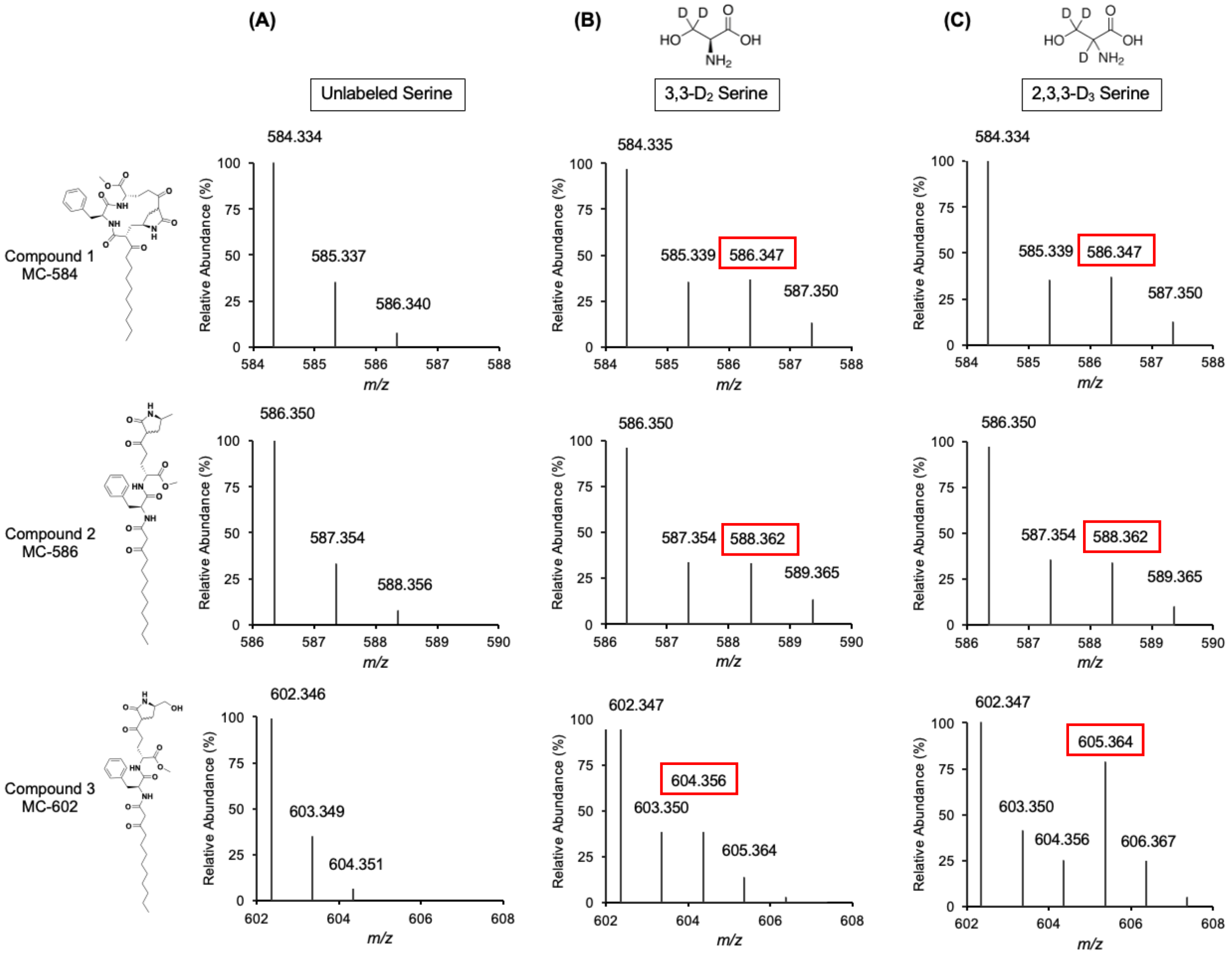

Figure S15: 3,3-D$_2$ and 2,3,3-D$_3$ serine feeding. HRMS data show compounds **1**, **2**, and **3** after addition of A) no isotope labeled serine, B) 3,3-D$_2$ serine, and C) 2,3,3-D$_3$ serine. For each labeled isotope, the enriched *m/z* $[M+H]^+$ is highlighted in a red box.

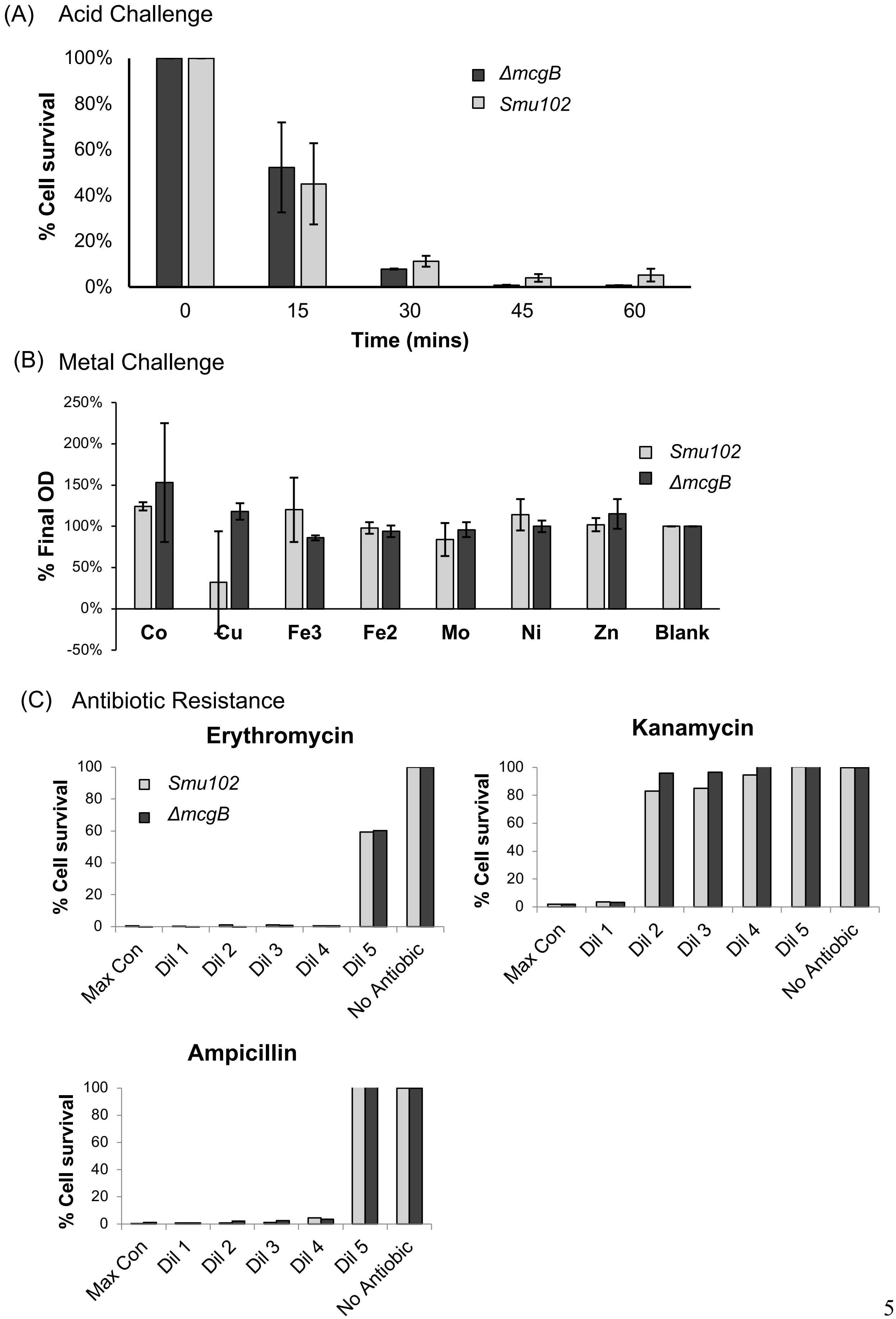
(A) Acid Challenge
100%
80%
60%
40%
20%
0%
% Cell survival
ΔmcgB
Smu102
0
15
30
45
60
Time (mins)
(B) Metal Challenge
250%
200%
150%
100%
50%
0%
-50%
% Final OD
Smu102
ΔmcgB
Co
Cu
Fe3
Fe2
Mo
Ni
Zn
Blank
(C) Antibiotic Resistance
Erythromycin
Kanamycin
Ampicillin
% Cell survival
Smu102
ΔmcgB
Max Con
Dil 1
Dil 2
Dil 3
Dil 4
Dil 5
No Antiobic

Figure S16: Bioactivity assays with Smu102 and *ΔmcgB*. (A) Acid challenge assay with *Smu102* and *ΔmcgB*. *S. mutans* cells were exposed to a low pH of 2.5 and incubated for one hour. Their survival at 15min time increments were determined via OD600 measurements. (B) Metal challenge assay with Smu102 and *ΔmcgB*. Metals were supplemented at 100µM to examine changes in overnight growth. Cell growth was measured with OD600. Percent final OD was calculated by comparing the final OD600 to the control sample with no metal added (blank). (C) Antibiotic resistance assay with Smu102 and *ΔmcgB*. Erythromycin, kanamycin, and ampicillin were dosed at their working concentrations of 50µg/mL, 100µg/mL, and 50µg/mL, respectively, as a maximum concentration control. The antibiotics were then serially diluted 4x five times. Cell growth was measured after 16 hours via OD600 and compared to growth under no antibiotics. Percent cell survival was determined via comparison to growth under no antibiotics.

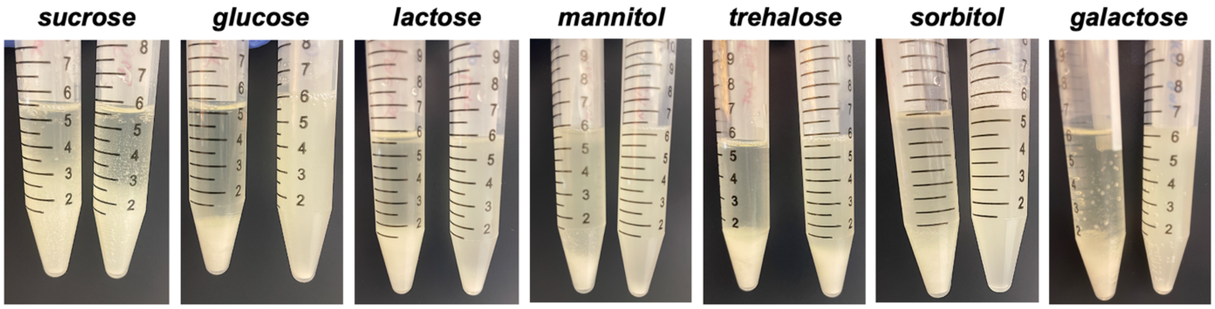

Figure S17: Planktonic growth of Smu102 and *ΔmcgB* with different carbon sources. For each carbon source, Smu102 is on the left and *ΔmcgB* is on the right. All cultures were grown in CDM base media and spiked with 1M of their respective carbon sources.

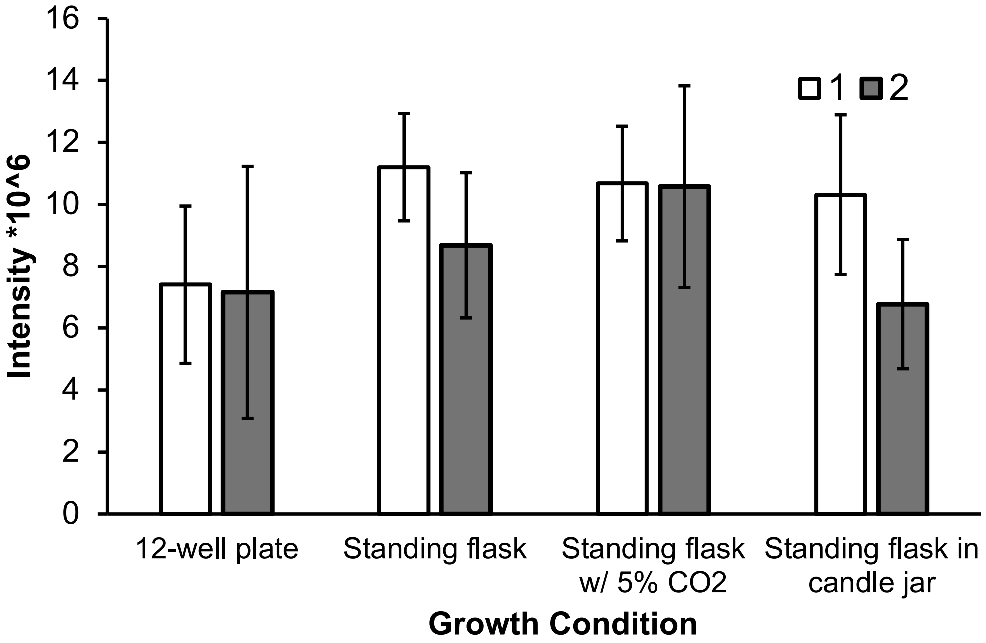

Figure S18: Production of 1 and 2 in different culture conditions. Production of **1** and **2** were measured via LC-HRMS. Error bars represent standard deviations of three biological replicates. All standing flasks were 5mL and grown at 37°C. The 12-well plate had 3mL of cultures.

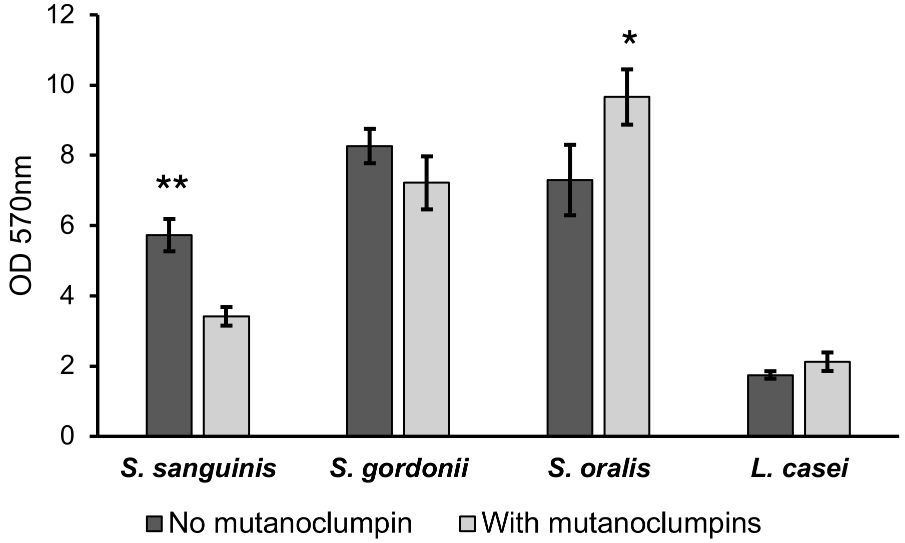

Figure S19: Effect of 1 and 2 on representative oral commensals. Compounds **1** and **2** were both added to a final concentration of 8μM, and cultures were inoculated at 5% v/v during early exponential phase. *Streptococcus sanguinis* SK36*, Streptococcus gordonii* DL-1*, Streptococcus oralis* SK139*,* and *Lactobacillus casei* ATCC4646 were grown in their respective biofilm formation medias: Biofilm media, BHI + 1% sucrose, TSB, and MRS media, respectively. Error bars represent standard deviations of three biological replicates. All 12-well plates were grown at 37°C for 24 hours. Biofilm biomass was calculated via crystal violet assay at OD570. A two-tailed t-test assuming equal variances was performed on each of the conditions. * represents a p-value <0.05 and ** represents a p-value <0.005.

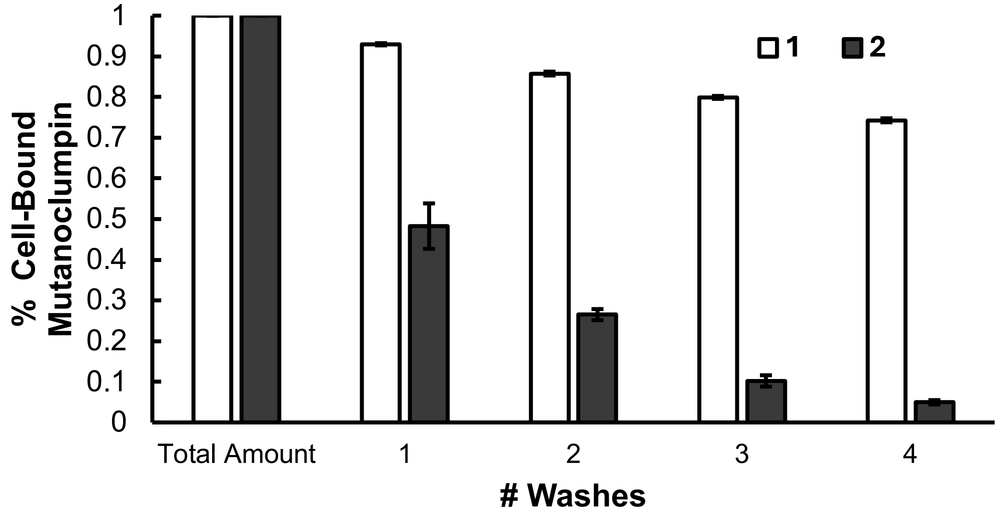

Figure S20: Relative strength of binding of compounds 1 and 2. Percent cell-bound **1** or **2** with *ΔmcgB* after 4 vigorous washing cycles. Compound was allowed to bind to cells for 10 minutes after the compound was initially added. Compound was added at 2μg/mL.

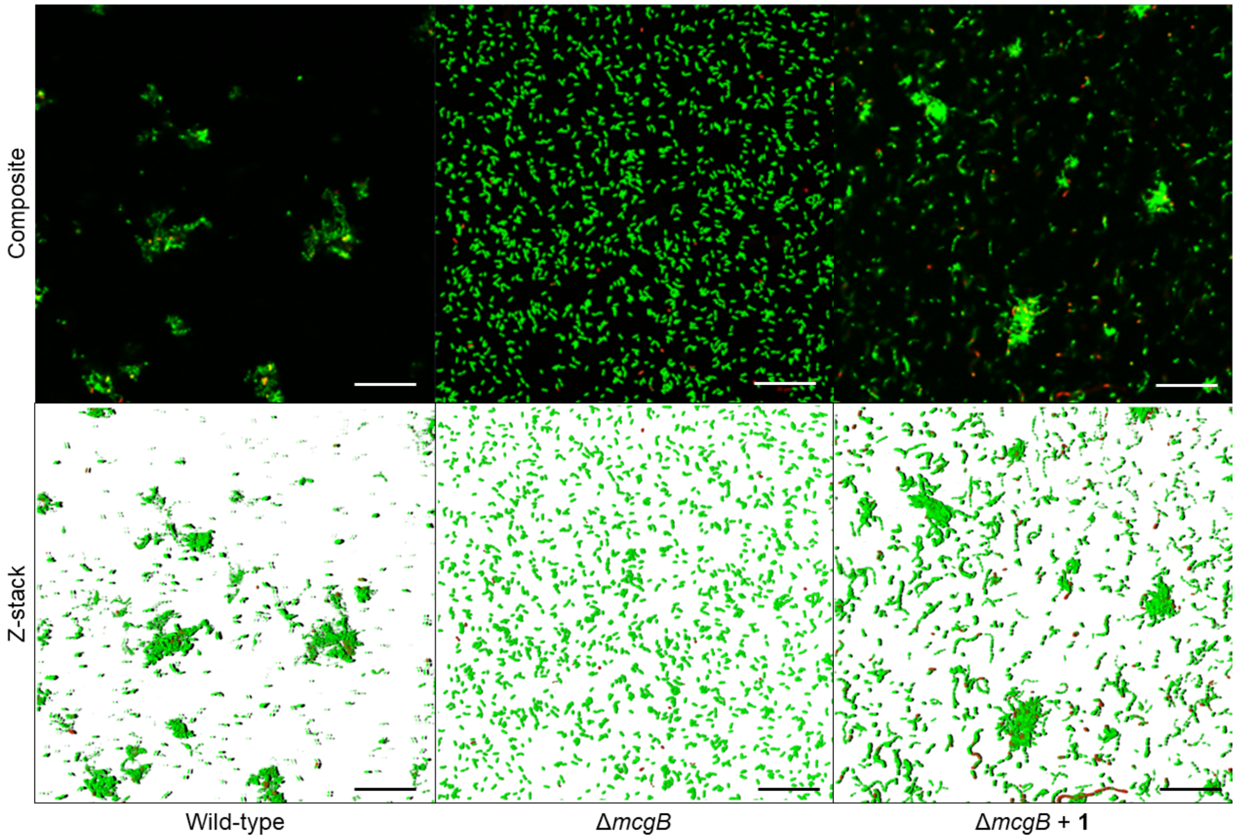

Figure S21: Confocal scanning laser microscope images of Smu102 (left), Δ*mcgB* (center), and Δ*mcgB* + **1** under 63x objective. Top: 2-dimensional composite images from red (propidium iodide) and green (Syto9) channels; bottom: 3-dimensional reconstructions from Z-stack images with the same field of view as above. 20 µm scale bar included in bottom right of each image. Clump height estimates were calculated via z-stack imaging calculations.

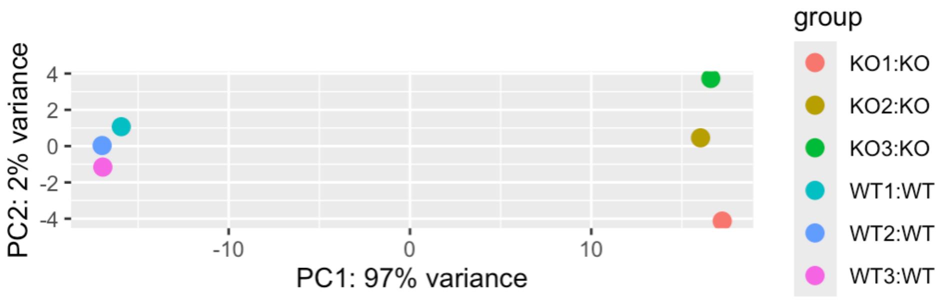

Figure S22: Principal Component Analysis (PCA) of RNA-seq data. The PCA plot[5] shows the distribution of RNA-Seq samples based on their gene expression profiles. Each point on the graph represents a single sample. The x-axis (PC1) and y-axis (PC2) represent the first and second principal components, respectively, which capture the highest variance in the dataset. Samples within the same experimental condition cluster together, indicating similarity in their gene expression profiles.

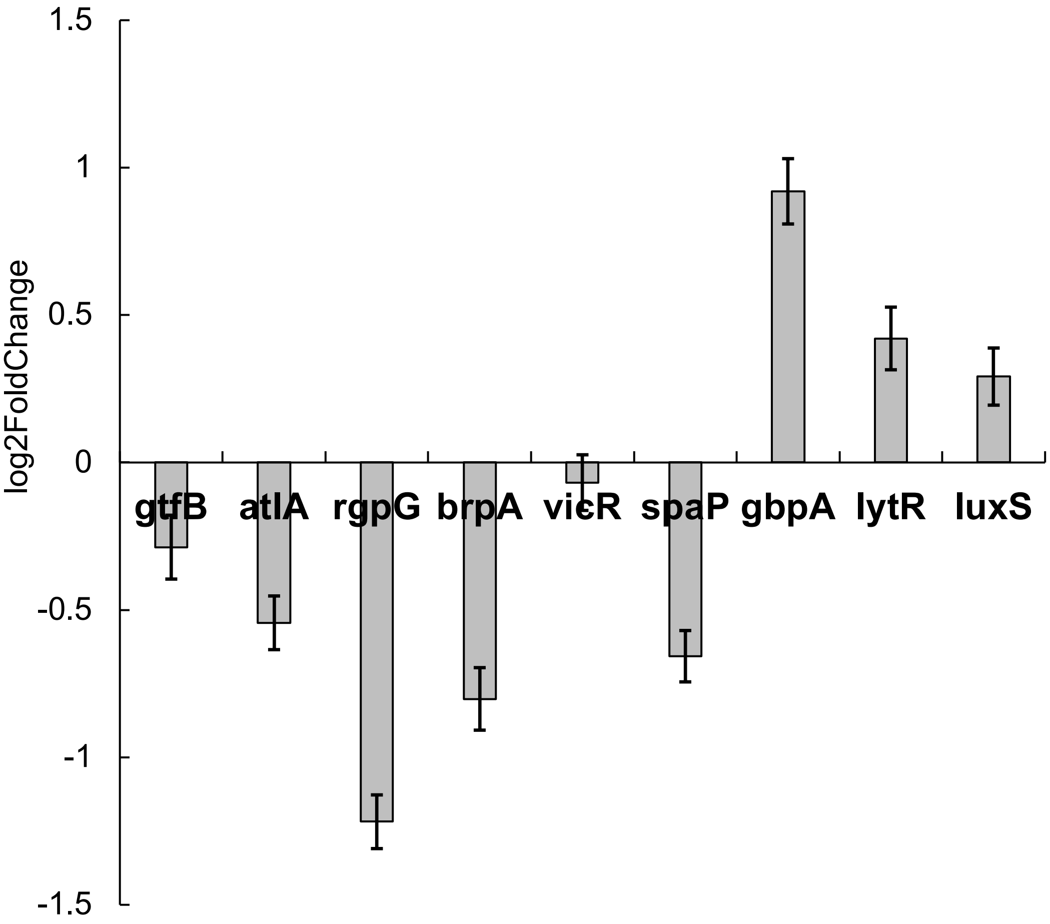

Figure S23: RNA-seq log2 fold change of common biofilm associated genes in *ΔmcgB*. *ΔmcgB* gene expression compared to Smu102 grown in CDMB with glucose. Log2 fold changes were calculated with DESeq2[6]. The selected genes include sucrose-dependent biofilm formation factors *gtfB* and *gbpA*, and sucrose-independent biofilm factors *spaP*, *atlA*, *rgpG*, and *lytR*. Master regulators *brpA*, *vicR*, and *luxS* were also included. Genes were considered significantly upregulated or downregulated if they had a log2 fold change >|2| and a p-value <0.05. None of the major biofilm associated genes were significantly upregulated or downregulated in *ΔmcgB.* Error bars represent standard error estimates of the log2 fold changes calculated by DESeq2 based on three biologically independently samples.

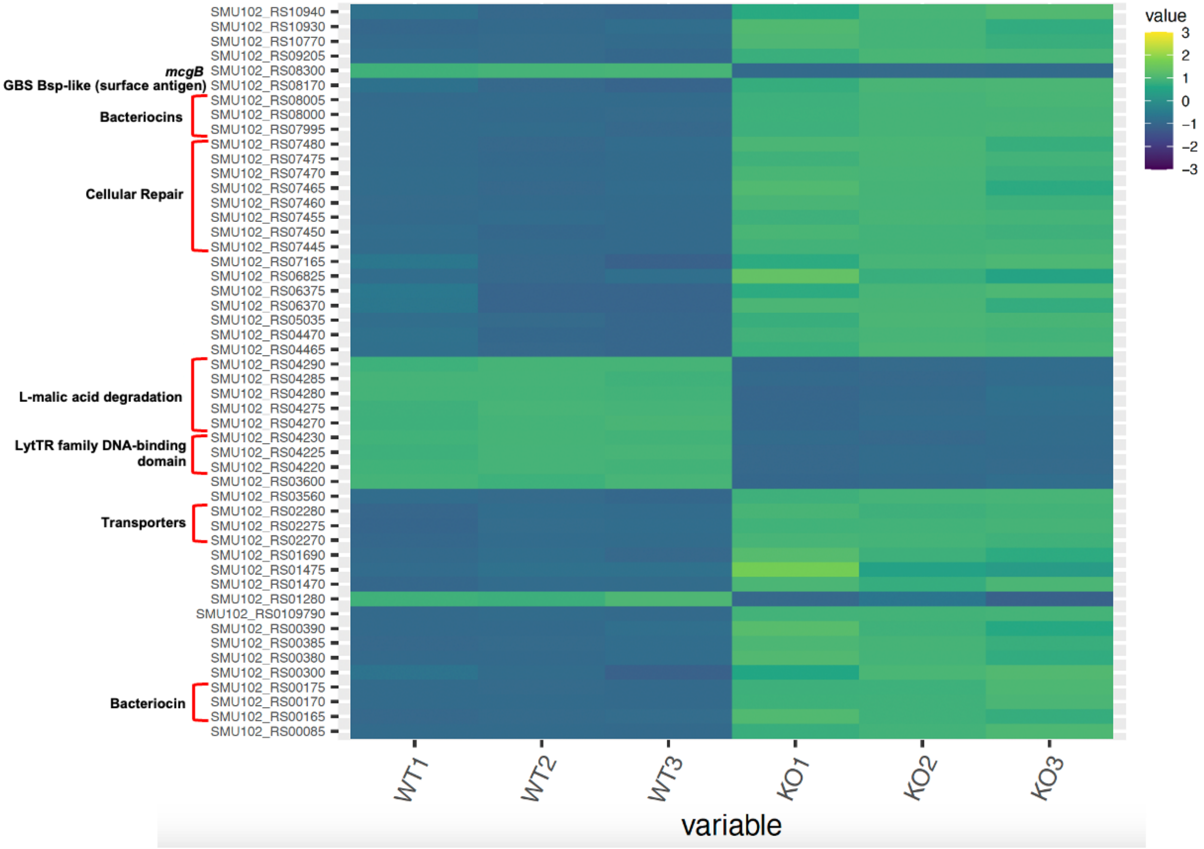

Figure S24: Heat map of most significant RNA-seq results comparing Smu102 and *ΔmcgB*. The heat map displays the z-score normalized gene expression levels of the most significantly different genes from three WT (Smu102) and three KO (*ΔmcgB*) samples. Each row corresponds to a gene, and each column corresponds to a sample. Where known, proposed functions of clusters of genes are given on the left. The color intensity represents the normalized z-scores of gene expression. A greater difference in z-scores within one gene signifies a larger difference in relative expression level.

Contig gap	Expected frag (bp)
7/74	336
74/93	414

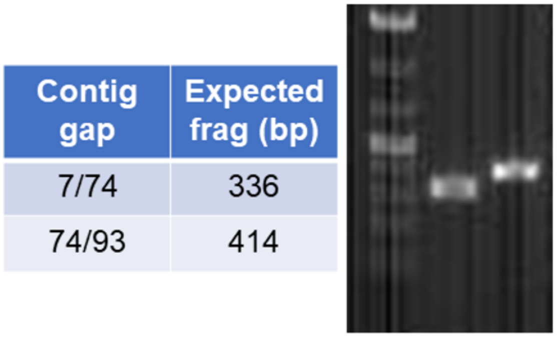

Figure S25: Contig gaps in *mcg* resolved for the Smu102 genome. Primer pairs were designed to amplify the overlapping regions on Smu102's contigs 7 and 74 and 74 and 93, according to the expected continuity of the *mcg* genes on *S. mutans* LAR01. The Invitrogen's 1kb plus DNA ladder was used for the DNA gel. Bands in the right panel match the expected sizes of amplified bands from primer pairs, calculated on the right.

Supplementary Notes

Note S1: Compound 1a/b NMR structure elucidation.

Structural Elucidation of Compound **1**. Compound **1** was isolated as an amorphous white solid. It had the molecular formula $C_{32}H_{45}N_3O_7$ based on a proton adduct ion at *m/z* 584.3347 $[M+H]^+$ and a sodium adduct ion at *m/z* 606.3152 $[M+Na]^+$ in its positive ion HRESIMS spectrum. Its ^1H NMR, COSY, and HSQC spectra displayed signals for one phenylalanine residue δ_H 9.29, -CO-NH-12; δ_H 4.74, H-13; δ_H 2.97, H-15a; δ_H 2.81, H-15b; δ_H 7.17 – 7.24, 5H, H-17/18/19/20/21), one glutamic residue δ_H 8.97, -CO-NH-2; δ_H 4.24, H-2; δ_H 2.17, H-3a; δ_H 1.66, H-3b; δ_H 2.54, H-4a; δ_H 2.37, H-4b), one methoxy group δ_H 3.59, H_3-22), one long chain moiety (δ_H 2.21, H-24a; δ_H 1.94, H-24b; δ_H 1.23 – 1.29, H_2-25/26/27/28/29/30/31; δ_H 0.86, H_3-32), one amide group δ_H 8.27, -CO-NH-7), two methylene groups δ_H 1.98, H-9a; δ_H 1.84, H-9b; δ_H 2.24, H-10a; δ_H 1.28, H-10b), and three methine groups δ_H 3.07, H-6; δ_H 3.48, H-8; δ_H 3.59, H-11). Furthermore, the ^{13}C NMR spectrum showed signals for two ketones δ_C 205.1, C-5; δ_C 204.6, C-23) in compound **1**. The presence of 2-pyrrolidone unit was indicated by their COSY correlations between H-6 and H-9a/b, and H-9a/b and H-8, as well as ^2J-HMBC correlations between -CO-NH-7 and C-7 (δ_C 172.4)/C-8 (δ_C 51.8) and ^3J-HMBC correlations between -CO-NH-7 and C-6 (δ_C 57.5)/C-9 (δ_C 31.0). Meanwhile, the macrocyclic skeleton was verified by their COSY correlations between H-8 and H-10a/b, and H-10a/b and H-11, besides ^2J-HMBC correlations between H-4/6 and C-5, H-11 and C-12 (δ_C 167.5), and H-13 and C-14 (δ_C 171.4) and ^3J-HMBC correlations between H-6 and C-4 (δ_C 34.0), H-13 and C-12, and H-15 and C-14. The side chain 1-decanone was connected to C-11 through ^2J-HMBC correlations between H-11/24 and C-23. Additionally, the methoxy group was linked to the glutamic residue, based on ^3J-HMBC correlation between H_3-22 and C-1 (δ_C 171.9). Finally, the absolute configurations of C-2 and C-13 are both assigned as *S* due to these two residues are from the natural amino acids, and the remaining chiral centers are assigned as 6*S*, 8*R*, 11*R* in compound **1a** and 6*R*, 8*R*, 11*R* in compound **1b** via comparison between the experimental and computational NMR data (**Table S4-8**).

Note S2: Compound 2a/b NMR structure elucidation

Structural Elucidation of compound **2**. Compound **2** was also isolated as an amorphous yellow solid with two isomers and had the molecular formula $C_{32}H_{47}N_3O_7$ based on its positive mode HRESIMS, which showed an intense peak at *m/z* 586.3487 $[M + H]^+$. The differences of 16 Da between the molecular weights of compound **2** and compound **3** implied the structure of compound **2** might be a deoxygenated product of compound **3**. Moreover, through a side-by-side comparison of its NMR spectroscopic data with those of compound **3**, the HSQC spectrum showed that one carbon signal at δ_C 64.3 (CH_2) in compound **3** was shifted to δ_C 22.0 (CH_3) in compound **2**, which indicated the deoxygenation occurred at C-10. Consequently, the absolute configurations of compound **2a/b** were assigned as the same as compound **3a/b**.

Note S3: Compound 3a/b NMR structure elucidation

Structural Elucidation of compound **3a/b**. Compound **3** was isolated as an amorphous yellow solid with two isomers. Its positive ion HRESIMS revealed a peak for a molecular ion at *m/z* 602.3436 $[M + H]^+$, corresponding to the molecular formula $C_{32}H_{47}N_3O_8$. The differences of 18 Da between the molecular weights of compound **1** and compound **3** implied the structure of

compound **3** might be an additional product of water and compound **1**. Furthermore, a side-by-side comparison of its NMR spectroscopic data with those of compound **1** showed them to be similar except for the C-10 and C-11 positions. The largest differences were observed in the HSQC spectrum for one methine group at C-11 position (δ_H 3.59; δ_C 54.7) and one methylene group at C-10 position (δ_H 2.24, 1.28; δ_C 32.1) in compound **1** compared to the methylene (δ_H 3.27, 3.22; δ_C 50.4) and the downfield methylene **(**δ_H 3.32, 3.22; δ_C 64.3) at the same positions in compound **3**, indicating that the C-10 C-11 bond was cleavage in compound **3**. With all the locations accounted for, the hydroxyl group was connected to C-10, and this assignment was verified by ^3J HMBC from H-9 (δ_H 2.40, 1.84) to C-10 (δ_C 64.3), and -CO-NH-7 (δ_H 7.95) to C-10. Finally, their absolute configurations of compound **3a/b** were assigned as the same as compound **1a/b**.

Supplementary Methods

Transcriptional profiling of WT and Δmcg

Three biological replicates of *S. mutans* Smu102 wildtype and Δ*mcgB* were grown for 14 hours in nonselective CDMB with 1M glucose. RNA was extracted in biological triplicate with TRIzol (Thermo-Fisher, San Jose, CA) according to the manufacturer's instructions. Sample purity was assessed by DNA-dependent amplification of *ldh* using PCR. Sample cleanup was performed using a Monarch® Total RNA Miniprep Kit (New England Biolabs, Ipswich, MA, USA). Then, the mRNA was fragmented, cDNA was reverse transcribed with random hexamer primers, and the library was prepared and sequenced on Illumina Novaseq platform with the paired-end mode and read length of 150 bp. Approximately 2 Gb of raw reads were generated for each sample. The low-quality sequences were trimmed from the raw Illumina reads with Trimmomatic[7]. Quality checked sequences were then aligned to the Smu102 reference genome (NCBI Accession GCA_000339055.1) with STAR[8] and gene hits were counted with FeatureCounts[9]. DESeq2[6] analyzed the differential gene expression with default parameters. Only log2 fold-changes over 2 were considered to be a significant difference. A principal component analysis (PCA) plot was generated via DESeq2 based on normalized expression to display differentiation between Smu102 and Δ*mcgB*. Protein sequences from significant genes were queried via BLASTP[10] and NCBI's protein database[11] to search for known protein homologues.

Acid challenge assay

Acidurity was assayed as previously described[12–14] with modifications. *S. mutans* Smu102 and *ΔmcgB* were grown overnight in CDMB. To prepare log phase cells for acid challenge, cells were grown to log phase in CDMB at pH = 7.0 to OD = 0.1. The cultures were gently sonicated for 15sec to disperse the chains of cells before inoculation into acidic media. Cells were inoculated into fresh CDMB and adjusted to pH 2.8 with HCl. An aliquot of cell suspension from Smu102 and *ΔmcgB* were inoculated onto THB agar plates at 0, 15, 30, 45, and 60 minutes to show change in cell survival over time. Colonies were counted after two days of growth at 37°C. Percent cell survival was calculated by dividing the number of colonies at each time point by the number of colonies at time = 0 minutes.

Metal challenge assay

Metals were supplemented at 100 μM concentrations to examine changes in growth. Deviations in growth rates between Smu102 and *ΔmcgB* might indicate differences in metal acquisition. Percent growth change was measured by dividing the final OD_{600} of each culture by the final OD_{600} of its respective control culture (Smu102 or *ΔmcgB*) with no added metal.

Antibiotic Resistance

A variety of antibiotics for which *S. mutans* is typically susceptible to were tested. These included erythromycin, kanamycin, and ampicillin. As a control, each of these compounds were dosed at their working concentrations of 50 ug/mL, 100 ug/mL, and 50 ug/mL respectively. Then, samples underwent four serial dilutions of each of the antibiotics until growth was observed. The end point OD_{600} was recorded for each sample after 18 hours of overnight growth at 37°C.

Feeding experiments for the biosynthetic pathway study

1% (v/v) Smu102 was inoculated from an overnight culture into CDMB supplemented with 0.5 mg ml^{-1} of [1-^{13}C] Ser, [1-^{13}C] Ala, [3,3,2-D_3] Ser, or [3,3-D_2] Ser (all the reagents were purchased from Cambridge Isotope Laboratories, Inc.). Cultures were grown for 18 hours at 37 °C, standing. After incubation, each sample was treated as described in the main methods for LC–HRMS analysis.

NMR characterization

The ^{1}H, ^{13}C, ^{1}H–^{1}H COSY, ^{1}H–^{13}C HSQC and ^{1}H–^{13}C HMBC NMR spectra for **1a**, **1b**, **2**, and **3.** were acquired, respectively, on a Bruker Avance 900 NMR spectrometer (900 MHz for ^{1}H and 225 MHz for ^{13}C) equipped with a cryoprobe. For the NMR tests, both samples were dissolved in DMSO-d_6 (Cambridge Isotope Laboratories, Inc.) and loaded into 2.5-mm NMR tubes. Data were collected and reported as follows: chemical shift, integration multiplicity (s, singlet; d, doublet; t, triplet; m, multiplet) and coupling constant. Chemical shifts were reported using the DMSO-d_6 resonance as the internal standard for ^{1}H-NMR DMSO-d_6: $\delta = 2.50$ p.p.m. and ^{13}C-NMR DMSO-d_6: $\delta = 39.5$ p.p.m. NMR computational predictions were made with assistance from Gaussian16[16].

GNPS Molecular Network analysis

A molecular network was created on the GNPS website (http://gnps.ucsd.edu). The data was filtered by removing all MS/MS fragment ions within +/- 17 Da of the precursor *m/z*. MS/MS spectra were window filtered by choosing only the top 6 fragment ions in the +/- 50Da window throughout the spectrum. The precursor ion mass tolerance was set to .05 Da and a MS/MS fragment ion tolerance of 0.2 Da. A network was then created where edges were filtered to have a cosine score above 0.4 and more than 4 matched peaks. Further, edges between two nodes were kept in the network if and only if each of the nodes appeared in each other's respective top 10 most similar nodes. Finally, the maximum size of a molecular family was set to 100, and the lowest scoring edges were removed from molecular families until the molecular family size was below this threshold. The spectra in the network were then searched against GNPS' spectral libraries. The library spectra were filtered in the same manner as the input data. All matches kept between network spectra and library spectra were required to have a score above 0.7 and at least 6 matched peaks.

Confocal scanning laser microscopy

To observe cellular architecture, confocal scanning laser microscopy (CLSM) was performed. To profile autoaggregates, *S. mutans* S1B wild-type and Δ*mcgB* were in 5 mL of CDMB overnight. To profile biofilms, cultures were grown in six-well plates with sterile coverslips. Cultures were stained using a LIVE/DEAD BacLight Bacterial Viability Kit (Molecular Probes, Eugene, OR) according to the manufacturer's instructions.[15] Stained cultures were observed using a Zeiss LSM 880 Confocal microscope with a 63x objective using green (488-nm excitation, green emission) or red (543-nm excitation, red emission) channels. Three-dimensional images were reconstructed using Z-stack imaging optimized in silico using the ZEN software package.

Wash assays

Δ*McgB* was grown from a single colony for 16 hours at 37 °C and then re-inoculated in fresh CDMB to reach mid-stationary phase at OD600 ~ 1.5. Cells were then washed and resuspended 2x in sterile PBS. For the wash assay, ~2 μg ml^{-1} of compound **1** or **2** was added to cells undiluted cells. Cells were left undisturbed for 10 minutes and then centrifuged at 16,000g for 1 minute. Compound that remained in the supernatant was defined as unbound to cells. The supernatant was removed, and compound was quantified via single quadruple LC-MS. Cells then underwent four wash cycles involving a ten second vigorous vortex with sterile PBS and then immediately centrifuging to measure the amount of compound washed off. The percent of cell-bound mutanoclumpin was determined by subtracting the unbound compound quantified via LC-MS from the total compound added and dividing by the total amount of compound added.

Supplementary References

1. Cornejo, O. E. *et al.* Evolutionary and population genomics of the cavity causing bacteria Streptococcus mutans. *Mol. Biol. Evol.* **30**, 881–893 (2013).
2. Xu, P. *et al.* Genome of the opportunistic pathogen Streptococcus sanguinis. *J. Bacteriol.* **189**, 3166–3175 (2007).
3. Navarro-Muñoz, J. C. *et al.* A computational framework to explore large-scale biosynthetic diversity. *Nat. Chem. Biol.* **16**, 60 (2019).
4. Wang, M. *et al.* Sharing and community curation of mass spectrometry data with Global Natural Products Social Molecular Networking. *Nat. Biotechnol.* **34**, 828–837 (2016).
5. Lever, J., Krzywinski, M. & Altman, N. Principal component analysis. *Nat. Methods* **14**, 641–642 (2017).
6. Love, M. I., Huber, W. & Anders, S. Moderated estimation of fold change and dispersion for RNA-seq data with DESeq2. *Genome Biol.* **15**, 550 (2014).
7. Am, B., M, L. & B, U. Trimmomatic: a flexible trimmer for Illumina sequence data. *Bioinforma. Oxf. Engl.* **30**, (2014).
8. Dobin, A. *et al.* STAR: ultrafast universal RNA-seq aligner. *Bioinformatics* **29**, 15 (2012).
9. Liao, Y., Smyth, G. K. & Shi, W. featureCounts: an efficient general purpose program for assigning sequence reads to genomic features. *Bioinformatics* **30**, 923–930 (2014).
10. Altschul, S. F., Gish, W., Miller, W., Myers, E. W. & Lipman, D. J. Basic local alignment search tool. *J. Mol. Biol.* **215**, 403–410 (1990).
11. Marchler-Bauer, A. *et al.* CDD: NCBI's conserved domain database. *Nucleic Acids Res.* **43**, D222-226 (2015).
12. Li, Y. H., Hanna, M. N., Svensäter, G., Ellen, R. P. & Cvitkovitch, D. G. Cell density modulates acid adaptation in Streptococcus mutans: Implications for survival in biofilms. *J. Bacteriol.* **183**, 6875–6884 (2001).
13. Lembo, F. L., Longo, P. L., Ota-Tsuzuki, C., Rodrigues, C. R. M. D. & Mayer, M. P. A. Genotypic and phenotypic analysis of Streptococcus mutans from different oral cavity sites of caries-free and caries-active children. *Oral Microbiol. Immunol.* **22**, 313–319 (2007).
14. Wen, Z. T. & Burne, R. A. LuxS-Mediated Signaling in Streptococcus mutans is Involved in Regulation of Acid and Oxidative Stress Tolerance and Biofilm Formation. *J. Bacteriol.* **186**, 2682–2691 (2004).
15. Tawakoli, P. N., Al-Ahmad, A., Hoth-Hannig, W., Hannig, M. & Hannig, C. Comparison of different live/dead stainings for detection and quantification of adherent microorganisms in the initial oral biofilm. *Clin. Oral Investig.* **17**, 841–850 (2013).
